# Supplementary material for: Adaptive hierarchical origami-based metastructures
Source: Nat Commun. 2024 Jul 26;15:6247. doi: 10.1038/s41467-024-50497-5 (PMC11282231; doi:10.1038/s41467-024-50497-5)
Supplement: Supplementary file 1 — Supplementary Information [file 41467_2024_50497_MOESM1_ESM.pdf]

Supplementary Information

Adaptive hierarchical origami-based metastructures

Yanbin Li[1,3*], Antonio Di Lallo[1,2,3], Junxi Zhu[1,2], Yinding Chi[1], Hao Su[1,2*], Jie Yin[1*]

[1]Department of Mechanical and Aerospace Engineering, North Carolina State University, Raleigh, NC, 27606, USA.

[2]Lab of Biomechatronics and Intelligent Robotics, Joint NCSU/UNC Department of Biomedical Engineering, North Carolina State University, Raleigh, NC, 27695, USA.

[3]These authors contributed equally

[*]Corresponding authors: yli255@ncsu.edu (Y.L.), hsu4@ncsu.edu (H.S.), jyin8@ncsu.edu (J.Y.)

This file includes:

Supplementary Note 1. Comparison with existing reconfigurable systems

Supplementary Note 2. Combinatorial design of hierarchical origami-based structures

Supplementary Note 3. Definition of the four categories of the level-2 structures

Supplementary Note 4. Theoretical framework underpinning shape morphing of hierarchical origami metastructures

Supplementary Note 5. Demonstration of the flexible length of level-2 links

Supplementary Note 6. Reconfiguration DOFs of the selected level-2 structure in Fig. 3

Supplementary Note 7. Reconfiguration kinematic analysis of level-1 and level-2 structures

Supplementary Note 8. Quantifying the number of reconfiguration paths of level-2 structure

Supplementary Note 9. Inverse design of level-2 structure for imitating target shapes

Supplementary Note 10. Structural stability and loading capacity of the reconfigured shapes

Supplementary Note 11. Untethered and remotely controlled designs

Supplementary Note 12. Demonstration of the generality of the proposed hierarchical design principle

Supplementary Table 1

Supplementary Fig. 1 to Fig. 28

Captions for Supplementary Movie 1 to Movie 8

Supplementary Note 1. Comparison with existing reconfigurable systems

In comparison to previous works, our current work addresses several unsolved challenges. These challenges include:

Tradeoff between shape morphing versatility and controllable actuation: Versatile shape-morphing capability is crucial for enabling multifunctional applications in engineered structures. They can promptly adapt to fast changing external environments and diverse application scenarios by reconfiguring into on-demand shapes.

The versatility of shape morphing is directly linked to a structure's mobility, as indicated by its degrees of freedom (DOF). Theoretically, structures with a higher number of DOF tend to offer more extensive shape morphing capabilities, allowing them to assume numerous configurations. However, higher DOF can pose practical challenges for actuation. In real-world applications, each DOF requires a corresponding distributed actuator. Excessive DOF can lead to increased hardware requirements and complicate the associated control systems (software). Consequently, this complexity hinders the broad applicability of such structures. This tradeoff between theoretically feasible shape morphing versatility and practical controllability in terms of actuation remains a significant challenge, particularly in the fields of reconfigurable metastructures and robotics.

To address this challenge, numerous studies have been dedicated to overcoming the tradeoff between shape morphing versatility and controllability. However, as summarized in Supplementary Table S1 and discussed in the following section, previously designed shape morphing structures either sacrifice shape morphing versatility to achieve feasibility in control and actuation, or integrate many actuation systems, resulting in inefficient configuration changes and prolonged reconfiguration times. To date, solutions have prioritized either versatility or actuation, but fail to achieve both. Specifically, utilizing few number of easily controllable actuation DOFs for versatile shape morphing structures has yet to be achieved. This limitation significantly impedes their applications in areas such as reconfigurable metamaterials, robotics, building, and space infrastructure, where rapid, energy-efficient shape changes with simple kinematics and adaptable configurations are essential for fast changing application scenarios.

Design generality: While previous researches have primarily focused on designing reconfigurable structures with unique structural forms, our work introduces a fundamental and universal design scheme for constructing reconfigurable hierarchical origami metastructures. Our design scheme offers a broad design space, featuring a hierarchical architecture strategy that is both generalizable and applicable to the construction of similar reconfigurable hierarchical metastructures. These structures incorporate shape-morphing spatial closed-loop mechanisms, facilitating easy actuation and control while enabling rich shape morphing capabilities.

In the following sections, we compare our work with the state-of-the-art reconfigurable systems and our previous study in Ref. 36, as summarized in Supplementary Table S1.

Supplementary Note 1.1 Comparison with the state-of-the-art reconfigurable systems

In Supplementary Table S1, we provide detailed comparisons between our work and previous shape-morphing structures. We have selected the five most representative types of shape-morphing structures, which include (a) continuous structural forms, (b) rigidly rotatable kinematic mechanisms, (c) origami/kirigami structures, (d) mechanical metamaterials/metastructures, and (e) assemblies of discrete modules. To ensure fair comparisons, we consider the most critical aspects that characterize the shape-morphing capabilities of each reconfiguration structure. These aspects include reconfiguration DOFs, time, steps, and kinematics, achievable shapes, re-programmability, inverse design capability, and functionalities.

It is worth noting that the most desirable shape-morphing systems are capable of morphing into a vast number of distinct shapes efficiently, with minimal reconfiguration time, steps, and DOFs, while employing simple kinematics. They should also be programmable and even reprogrammable to morph into a higher number and more categories of shapes, with the ability to be inversely designed to achieve target geometries. Furthermore, the reconfigured shapes should be able to perform various on-demand functionalities. After thorough comparison, we find that our proposed hierarchical origami structures outperform previously designed shape-morphing structures in all the aforementioned aspects. Detailed explanations are provided below.

a. Continuous structural form-based reconfigurable systems[3, 5, 6, 36]. Essentially, these design strategies and our work represent two distinct approaches of shape-morphing structure designs. While the former methods typically aim to construct functional structures with 3D curved surfaces, featuring non-zero Gaussian curvatures through the nonlinear bending deformation of the entire body, our focus lies in constructing robotic structures/systems with volumetric shapes. Both approaches have their own specific applications and can be programmable to perform inverse design to imitate target shapes. However, significant limitations exist in the continuous structure-based system, including:

1. The quantity of achievable reconfiguration shapes is often limited to a single 3D curved shape.
2. Reconfiguration kinematics tend to be complex due to nonlinear elasticity.
3. Reconfiguration time is normally long and determined by the properties of the materials used.
4. The functionality of reconfigured shapes is often restricted to specific tasks, such as object manipulation or locomotion.

b. Kinematic mechanism based reconfigurable systems[7, 8, 10, 11, 13, 32, 42]**.** There are two types of kinematic mechanism-based reconfigurable structures: one utilizes chain linkages, while the other employs looped structural forms.

Chain linkage-based reconfigurable structures, such as Wacky Tracks fidget toys[11], typically feature a large number of reconfiguration DOFs due to the independence of hinges. Consequently, these structures face several limitations:

1. Increased reconfiguration steps are required to reshape into complex structures.
2. The complex actuation systems entail tedious reconfiguration steps.
3. They lack programmability and re-programmability, as well as the inverse design to imitate target shapes.

Previous works have also explored the design possibilities based on looped mechanisms to construct shape-morphing structures. However, constrained by unique structural topology, these structures typically exhibit only a single reconfiguration DOF, resulting in one reconfigured shape. For example, one particular reconfigurable structure based on 8R looped kinematic mechanisms is proposed. However, due to the lack of mutual interactions between each linkage, this structure can only reconfigure by flipping its structural elements inside out. Consequently, the versatility of shape morphing and functionalities are very limited. Moreover, almost all previous works[22,32,42] based on this method lack design re-programmability and are deficient in inverse designs.

c. Origami/kirigami based reconfigurable systems[15, 16, 18, 22, 26, 29, 31, 36, 38-41, 48]**.** Origami and Kirigami structures represent two prominent design strategies for creating reconfigurable structures with desired functionalities. However, most previous origami/kirigami-based reconfigurable structures[15,16,18,22] can only reconfigure into particular shapes for specific

applications[26,29,31,40,41,48] once fabricated with certain prescribed fold and/or cut patterns. Consequently, these structures face several limitations:

1. They are restricted by pre-assigned fold and/or cut patterns, resulting in only one or few reconfiguration DOF.
2. They lack the capability to be programmed and re-programmed to achieve more shapes.
3. They lack inverse design capability to imitate multiple target shapes.
4. They have limited applications due to the single or fewer reconfigured shapes.

While attempts have been made to create origami-based reconfigurable structures that can change into multiple shapes, such as those based on triangular structural elements[26], these structures often exhibit too many DOF to change into certain complex shapes. Controlling many reconfiguration DOFs requires complex hardware, such as a large number of electrical servomotors and control panels. Developing efficient reconfiguration algorithms to achieve complex shapes further complicates their practical applications. Moreover, these origami reconfigurable structures are typically applicable only to triangular-shaped structural elements and cannot be re-programmed or extended to other shaped structural elements.

d. Modular reconfigurable systems assembled by discrete modules[23-25, 27, 28]. This type of reconfigurable system involves assembling a certain number of discrete modules into desired shapes. Due to its capability of achieving almost all volumetric shapes, extensive researches have been conducted in modular reconfigurable robotics[23-25,27, 28]. In terms of reconfiguration kinematics, it can easily transit between different shapes by relocating or adding more discrete modules to target positions. However, this approach has several drawbacks:

1. Tedious reconfiguration steps and lengthy reconfiguration time are required to achieve shapes with complex geometries and transition between different configurations.
2. Optimized reconfiguration algorithms are needed to efficiently reconfigure into target shapes.
3. Exceptionally high reconfiguration DOF.
4. High cost control systems are required since each module requires independent actuation and control systems.
5. Any malfunctioning cubes (e.g., mechanical or battery issues) can abruptly terminate a reconfiguration process, as all cubes are involved during shape morphing.

Ref. 23 represents one of the most notable works using cube-shaped modules to construct modular reconfigurable robots. Each cube was individually integrated with actuation and control systems. To achieve target shapes, all the cubes are needed to continuously and repeatedly rotate to reach target positions, resulting in a longer time to shape into configurations with a large number of cube modules. Moreover, since all the cubes have to be stacked to stand stable, this design strategy could not achieve configurations with internal volumes. Similar issues exist in other modular reconfigurable systems summarized in Ref. 25, including lattice, chain, or hybrid architectures by assembling different shaped modules.

Therefore, although these modular reconfigurable robotic structures offer enhanced shape-morphing capabilities, their redundant DOFs induce reconfiguration complexity, resulting in lengthy and intricate reconfiguration steps, complex and time-consuming actuation, and complex morphing kinematics and reconfiguration paths, making it inefficient for applications.

e. Metamaterial/metastructures based reconfigurable systems[38, 39, 47, 48]. Reconfigurable systems based on mechanical metamaterials/metastructures can be classified into two categories. The first type[4, 35, 36] exhibits shape morphing similar to structures with continuous structural forms.

Even when constructed from periodic/aperiodic structural components, they can be equivalently treated as continuous thick plates and change shape over the whole body scale.

The second type features 3D bulky and volumetric structural forms[38,39,48]. These reconfigurable mechanical metamaterials/metastructures typically change shapes by twisting, shearing, and bending their building blocks. Due to their volumetric structural forms, these reconfigurable systems usually exhibit fewer degrees of freedom (DOFs) and thus a limited number of achievable shapes. Additionally, converting these architected materials into robotic structures presents new and significant challenges in terms of actuation and control. For example, the work in Ref. 39 demonstrates a reconfigurable 3D structure built from unit cells consisting of six extruded cubes. Actuated by four distributed pneumatic actuators, the unit can transform into four different configurations easily. However, scaling up the unit to a 4×4×4 periodic larger structure would require 96 distributed actuators to achieve similar three-state transformations, which makes the actuation systems rather tedious.

Advantages of our proposed hierarchical origami systems:

By incorporating closed-loop mechanisms within and across each hierarchical level to minimize the number of actuated DOFs, our proposed hierarchical origami metastructures can eliminate the need to actuate each individual DOF in previous reconfigurable origami metamaterials and robots, while achieving promising shape morphing versatility and complexity with fewer reconfiguration DOF and simple actuations. As a result, this advancement opens up possibilities for widespread applications, including the construction of highly efficient reconfigurable robots, as well as the rapid deployment of reconfigurable architecture on Earth and potentially in space, serving as multitask space robots and habitats.

In certain overlapping application scenarios, our work demonstrates advantages over the continuous structure-based strategy:

1. For load-bearing architectural applications, our approach can feasibly extend to meter-scale structures, as demonstrated in Figure 7.
2. In robotic applications, our work is multifunctional and capable of achieving multi-gait locomotion with carried load, as illustrated in Figure 5.

Supplementary Note 1.2 Comparison with our previous work in Ref. 36

In Ref. 36, we utilized a reconfigurable unit composed of eight closed-loop cubes with a special hinge connection. Directly assembling these 8-cube units into a tessellated form is challenging due to geometric frustrations and collisions among the shape-morphing units. In our previous work, we addressed this by creating a minus sign-shaped building block, merging the hinges of three different 8-cube units to avoid frustrations and collisions. We then tessellated these building blocks by further purposefully merging and modifying the connections between the building blocks. This ad-hoc and specialized design allowed us to assemble the units and building blocks into a periodic compact form. When assembled into a 5×5 array, this structure theoretically offers over 10,000 different reconfigured shapes through bifurcation. Although the structure has multiple closed-chain loops, these loops transform as independent units, with minimal coupling in their shape morphing due to the requirement for compatible deformation conditions at the interaction sections among units. This can still result in high mobility (over 10,000), necessitating complex control and actuation strategies. Therefore, despite the high potential for versatile shape morphing demonstrated by manual folding in our previous work, it poses significant challenges in terms of actuation and control for practical uses.

In contrast, in this work, our hierarchical design couples the spatial interactions of closed-chain loops within and across each hierarchical level to significantly reduce the active DOF to fewer than 3, while still preserving the versatility of shape morphing (over 1000 shapes). The reduced number of active DOF makes it practically feasible to actuate the versatile shape morphing, as demonstrated in this work. Furthermore, compared to the ad-hoc design, the hierarchical design offers several advantages:

1. Expanded design range: It allows for combinatorial connections within and across each hierarchical mechanism, which are either disabled or severely limited in our previous study.
2. Avoiding geometric frustration and collisions: This is achieved through compatible reconfigurations of differently leveled spatially looped mechanisms.
3. Versatile structural platform: This fundamental design principle establishes a platform that can be applied to various shaped building blocks, overcoming the limitations associated with specific structural elements in our previous ad-hoc design.

We have explored the underlying science of shape morphing in our proposed metastructures by developing new theoretical models. This includes four key points:

1. Rationally explaining all the obtained shapes.
2. Outlining the mathematical operations required to transform shapes.
3. Elucidating the reconfiguration kinematics that drive shape changes.
4. Introducing the inverse design principle for achieving target shapes.

Our previous study stopped at exploring the kinematics of a single unit cell composed of 8 cubes and did not explore the shape morphing of tessellated metastructures, which is the focus of the current study.

Lastly, both works utilize the concept of metastructures that derive their unprecedented properties from the architecture rather than the specific structural forms of the unit cell. We demonstrate that all the benefits and unprecedented properties are attributed to the distinct hierarchical architecture.

References		Reconfiguration DOF	Reconfiguration time	Reconfiguration steps	Kinematics	Achievable shapes	Re-programmability	Inverse Design	Functionalities
**Shape morphing structures with continuum structural forms**	3	Infinity	106s	1	Nonlinear bending	3D curved surfaces	✓	✓	Shape morphing
	5	Infinity	90s	1	Nonlinear bending	3D curved surfaces	✓	✓	Shape morphing
	6	Infinity	30-130s	1	Nonlinear bending	3D curved surfaces	✓	✓	Shape morphing, robotic running
	35	Infinity	<1s	1	Nonlinear bending	3D curved surfaces	✓	✓	Shape morphing, object manipulation
**Kinematic Mechanism based structures**	7	1	No actuation	1	Nonlinear rigid rotation	1	×	×	Shape morphing
	8	1	No actuation	1	Linear rigid rotation	1	×	×	Shape morphing
	10	1	No actuation	1	Nonlinear rigid rotation	1	×	×	Shape morphing
	11	2^N-1	No actuation	2^N-1	Linear rigid rotation	Exponentially related to unit number	✓	×	Shape morphing
	13	1	<3s	1	Nonlinear rigid rotation	4	×	×	Shape morphing
	32	1	No actuation	1	Nonlinear rigid rotation	1	×	×	Shape morphing
	42	1	No actuation	1	Linear rigid rotation	1	×	×	Shape morphing
**Origami/Kirigami based structures**	15	1	No actuation	1	Nonlinear rigid rotation	1	×	×	Architectural structure
	16	1	No actuation	1	Linear rigid rotation	1	×	×	Shape morphing
	18	1	No actuation	1	Nonlinear rigid rotation	1	×	×	Shape morphing
	22	1	No actuation	1	Nonlinear rigid rotation	1	×	×	Shape morphing
	26	>>1	Determined by the number of modules 10^1-10^2s)	Nonlinearly related with number of modules	Linear rigid rotation with complex planning algorithm	Exponentially related to unit number	✓ (By changing internal connectivity)	×	Shape morphing and object transporting
	29	1	10s	1	Nonlinear rigid rotation	1	×	×	Civil shelter
	31	1	1s	1	Linear rigid rotation	1	×	×	Surgical tool
	36	1	No actuation	Exponentially related to unit number	Linear rigid rotation	Exponentially related to unit number	✓ (By changing internal connectivity)	×	Shape morphing for architectural uses
	38	1 to 7	<10s	1	Linear rigid rotation	3	×	×	Shape morphing for architected materials
	39	1	No actuation	1	Linear rigid rotation	< 10	×	×	Shape morphing for architected materials
	40	1	<10s	1	Nonlinear rigid rotation	2	×	×	Shape morphing for driving wheel
	41	1	40s	1	Linear rigid rotation	1	×	×	Shape morphing for cargo frame
	48	1	<10s	1	Linear rigid rotation	<10	✓ (By changing internal connectivity)	×	shape morphing for functional exoskeletons
**Metamaterial and metastructure based**	38	1 to 7	No actuation	1 to 7	Linear rigid rotation	1 to 7	×	✓	metamaterial
	39	1	1 to 10s	1	Linear rigid rotation	1	×	×	metamaterial
	47	N-1	No actuation	Linearly related to achieved shapes	Linear rigid rotation	1	×	×	metamaterial
	48	1	No actuation	1	Nonlinear buckling	1	×	×	metamaterial
**Shape morphing structures assembled by discrete modules**	23	N	Determined by the number of modules (10^2s)	Exponentially related to number of modules	Repeating linear rigid rotation of all modules	3D volumetric structural profiles with infinity number	✓ (By changing internal connectivity)	✓	Shape morphing
	24	N	Determined by the number of modules (10^2s)	Exponentially related to number of modules	Repeating linear rigid rotation of all modules	Limited to certain 3D shapes(<10^2)	✓ (By changing internal connectivity)	×	Shaper morphing for locomotion robot and architectural structures
	27	N	Determined by the number of modules (10^1s)	Nonlinearly related to number of modules	Repeating linear rigid rotation of all modules	Limited to linkage structures (<10)	✓ (By changing internal connectivity)	×	Shape morphing
	28	N	Determined by the number of modules (10^2s)	Nonlinearly related to number of modules	Repeating linear rigid rotation of all modules	Limited only to 2D shapes	✓ (By changing internal connectivity)	✓	Swarming based locomotion
**This work**		**1 to 3**	**<10^2s**	**<10^1**	**Linear rigid rotation of low number of internal folds**	**Infinity due to the combinatorial design and the design generality**	✓	✓	**Shape morphing for architectural structures, locomotion, object transport, shelter; Metamaterials; Robotic structures**

Note 1: N is the number of structural components;
Note 2: Refs. 23, 24, 26, 27, 28, 36 and works summarized in [25] for fair comparison given their most related similarity with our work

Contd.

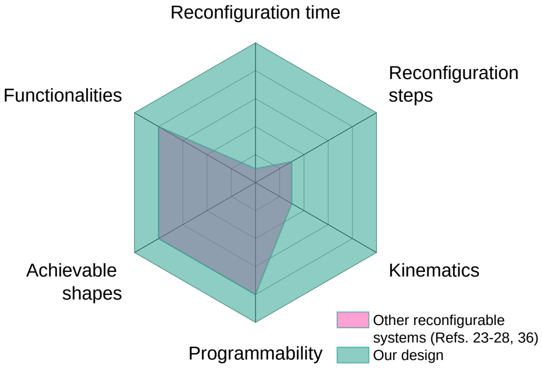

Supplementary Table 1 Comparison between this work and five most representative state-of-the-art reconfigurable systems. Radar chart: comparison of the reconfiguration performances between our proposed hierarchical origami structures with part of previous systems (mainly those assembled by discrete, some origami-based and kinematic mechanisms structures with high DOF).

Supplementary Note 2. Combinatorial design of hierarchical origami-based structures

The combinatorial designs can be achieved in several ways. First, one can manipulate the hinge connections between cubes at different leveled structures. The combinatorial edge connectivity between cubes, which determines the spatial folding patterns, provides a large design space for constructing level-1 structures. For any two neighboring cubes, there are four possible ways to connect their facing edges to rotate around the bonded edge. Theoretically, for an n-cube structure with n = 4, 6, and 8, there are 4^n possible combinatorial designs, amounting to 256, 4096, and 65536 designs, respectively. The combinatorial design details of level-1 structures can be seen in Supplementary Fig. 4a-4d. According to Supplementary Fig. 4c, level-1 structures based on 4, 6, and 8 cubes can be combinatorically designed with over millions of possibilities.

Moreover, the initial structural DOFs of level-1 structures are deterministically influenced by the hinges relative spatial positions. We particularly differentiate the combinatorically designed 4, 6, and 8-cube level-1 structures according to their different initial structural DOFs, and present this combinatorial design information in Supplementary Fig. 4d. According to the rational analysis in Supplementary Note 6, the 4, 6, and 8-cube based level-1 structures correspondingly have 2, 3, and 5 maximal initial structural DOFs. However, it is noteworthy that with the increased number of initial structural DOFs, the design possibilities of all these three level-1 structures decrease. This is because the hinges must be uniquely arranged into specific spatial patterns to ensure the occurrence of high initial DOFs.

Similar combinatorial hinge connections can also be applied to the joints at higher structural levels. For example, for a level-2 structure composed of four 8-cube level-1 structures shown in Fig. 2b and Supplementary Fig. 5, i.e., the <8R, 4R> structure, the four level-1 structures are connected through four pairs of facing edges (joints) in the cubes along two centerlines. Along the two orthogonal centerlines, i.e., the x and y-axes, there are 12 pairs of facing cubes: 8 along the x-axis and 4 along the y-axis. This configuration allows for a combinatorial number of $4^4 \times 4^2 =$ 4096 hinge connections at the four level-2 joints. By combining the combinatorial designs at level 1 and higher leveled joints, we enable a tremendous design space for constructing hierarchical

structures. For example, for the <8R, 4R> level-2 structure, if we only consider the $4^8 = 65536$ types of edge connectivity in one 8-cube level-1 structure, it results in approximately 0.27 billion ($4^8 \times 4^6$) potential designs.

Secondly, since all higher-level structures exhibit structural asymmetry along the thickness direction, we can employ a second combinatorial design strategy by selectively flipping the sub-level structures by 180°. For example, based on the <8R, 4R> level-2 structures, Supplementary Fig. 5C presents two representative design examples achieved by flipping two 8R level structures around the y-axis.

Thirdly, as shown in Supplementary Fig. 3, the combinatorial design can also be accomplished by manipulating the flexible link types and the number of joints at each hierarchical level, choosing from any of the 4 basic motifs: 2R, 4R, 6R, and 8R. For instance, for a level-2 structure with 4 level-2 joints (4R motif), the level-1 structures can be composed of 4, 6, or 8 cubes, denoted as <4R, 4R>, <6R, 4R>, and <8R, 4R>, respectively.

Supplementary Note 3. Definition of the four categories of the level-2 structures

Essentially, the four categories of level-2 structures are classified based on two factors: (1) structural symmetries and (2) the type of level-1 structure. However, it should be noted that this classification method is based on the condition that the level-2 structure is composed of four identical <8R> level-1 structures, as detailed in Supplementary Fig. 5a.

As depicted in Supplementary Fig. 5b, the first category (Category 1) structure is constructed by arranging four level-1 structures (schematically highlighted in blue) with both *xz*-plane and *yz*-plane symmetries. Category 2 structures are built by placing level-1 structures with 2-fold structural symmetry around the z-axis. Specifically, the two level-1 structures at the top-right and bottom-left corners (schematically highlighted in green) are obtained by flipping the other two level-1 structures (identical to those used in Category 1) around the y-axis by 180 degrees (see the right insets in Supplementary Fig. 5b). By then placing the flipped level-1 structures with 1-fold structural symmetry around the z-axis, we obtain the Category 3 and Category 4 level-2 structures, as illustrated in the right two schematic figures in Supplementary Fig. 5b and the inset in Fig. 2f.

It should be noted that the aforementioned four categories of level-2 structures are created using one of the 8R level structures with the highest structural symmetries in its hinges. Therefore, undoubtedly, new categories of level-2 structures can be constructed using other types of level-1 structures. Given the extremely high design possibilities, we leave this research for future endeavors.

Supplementary Note 4. Theoretical framework underpinning shape morphing of hierarchical origami metastructures

We explore the theoretical framework that underpins the shape morphing of our proposed hierarchical origami metastructures, encompassing two key aspects. Firstly, we present a mathematical description that encompasses all shapes, including both the initial configurations and the reconfigured ones. Secondly, we elucidate the operations involved in the transformations between mutually reconfigurable shapes. We verify our proposed theoretical framework using the results obtained from the level-2 structure depicted in Fig. 3a.

Indeed, the essence of the shape morphing of our proposed hierarchical metastructure relies on the spatial position changes of the structural components induced by the relative rotations of the internal folds. The reconfigured shapes are specially defined by the relative spatial positions of the internal structural components. To offer a clearer description of the shape morphing process,

we draw inspiration from graph theory, commonly applied in computer science. By constructing a data-tree-like diagram wherein reconfigured shapes are represented as data points, with internal rotation angles set to multiples of 90°, we establish a method applicable across all hierarchical origami metastructures outlined in this work. Through this approach, we can systematically elucidate the descriptions of reconfigured shapes and delineate the intricacies of their shape-changing processes.

Supplementary Note 4.1. Rational description of the reconfigured shapes

The geometry of the reconfigured shapes are topologically determined by the relative spatial positions of the included structural components. Therefore, the reconfigured shapes can be accurately decided if the spatial positions of the composed structural elements are determined.

For our hierarchical origami metastructures, the relative spatial positions of the cube components can be exactly described by the local coordinates defined at their body center. Then, we can mathematically describe the cube's spatial position through a spatial vector as $\boldsymbol{v}$ with form as

$$\boldsymbol{v} = \left(v_x, v_y, v_z\right)^T \tag{S1}$$

Therefore, for any given reconfigured shape, we can rationally describe it through a shape matrix **M** which contains all the spatial vectors of its structural components. Mathematically, the shape matrix can be expressed as

$$\mathbf{M} = \left(\boldsymbol{v}_1, \boldsymbol{v}_2, \boldsymbol{v}_3, \cdots, \boldsymbol{v}_k\right) \tag{S2}$$

where k is integer and determined by the structural motif it uses; for example for the (8R, 4R) type of level-2 structures, k is equal to 4×8 = 32 while for the 8R based level-1 structure, and k is equal to 8.

Supplementary Note 4.2. Rational analysis of the operations of the shape transformations of reconfigured shapes

The physical meaning of the mathematical operations on the data-tree diagram represents the shape transformations among mutually reconfigurable configurations. This operation relies on the spatial position changes of some related structural components. Adhering to the basic design principle illustrated in Supplementary Fig. 3, certain shape transformation paths are intrinsically linked with the distinctive rotations of particular line folds. Consequently, these rotations give rise to unique, path-dependent reconfigured shapes.

Conventionally, we can always refer to a transformation matrix **t** to mathematically describe the internal rotation induced shape changes of any given structures. Therefore, the operations of the shape changes of our proposed hierarchical origami metastructures are can be rationally represented by some unique transformation matrices.

From Note 4.1, for any two mutually transformable shapes, we can simply express them by two shape matrix **M** and **M**' (transformed from **M**). Then, we can rationally express the operations of the shape changes between shape **M** and **M**' by a generalized mathematical equation as

$$\mathbf{M}' = \mathbf{tM} \tag{S3}$$

where the transformation matrix with explicit form as $\mathbf{t} = (\mathbf{t}_1, \mathbf{t}_2, \mathbf{t}_3, \ldots\ldots, \mathbf{t}_k)^T$ with k determined by the number of structural components. Therefore, combining equations (S2) and (S3) we can determine the new configuration **M**' as

$$\mathbf{M}' = \left(\mathbf{t}_1\boldsymbol{v}_1, \mathbf{t}_2\boldsymbol{v}_2, \mathbf{t}_3\boldsymbol{v}_3, \cdots, \mathbf{t}_k\boldsymbol{v}_k\right) \tag{S4}$$

Particularly in three dimensions in triads of left-handed (x, y z) Cartesian coordinates systems, the counter-clockwise transformation matrices are with explicit forms as

$$\mathbf{t}_x = \begin{bmatrix} 1 & 0 & 0 \\ 0 & \cos\theta_x & \sin\theta_x \\ 0 & -\sin\theta_x & \cos\theta_x \end{bmatrix}, \mathbf{t}_y = \begin{bmatrix} \cos\theta_y & 0 & -\sin\theta_y \\ 0 & 1 & 0 \\ \sin\theta_y & 0 & \cos\theta_y \end{bmatrix}, \mathbf{t}_z = \begin{bmatrix} \cos\theta_z & \sin\theta_z & 0 \\ -\sin\theta_z & \cos\theta_z & 0 \\ 0 & 0 & 1 \end{bmatrix} \quad \text{(S5)}$$

with θ as the rotation angles.

It should note that the equations (S1) to (S5) give the general form to uncover the basic principles for shape description and transformation/operation. By the following, we validate the above theoretical framework through the shape morphing of level-2 structure illustrated in Fig. 3a.

Supplementary Note 4.3. Validation of the basic principles about the shape description and operations

Indeed, the shape transformation of a configuration is inherently dictated by the rotations of its internal folds. Thus, it becomes necessary to firstly understand the specifics of these folds—their relative spatial positions—in order to ascertain the rotation matrix and subsequent reconfigured shapes. Notably, the arrangement of folds is predetermined by adhering to the design principle of structural hierarchy, wherein specific structural motifs are chosen at each structural level (Supplementary Fig. 3c, i). Consequently, the relative spatial positions of the folds are invariably determined and traceable once their arrangements in the initial configuration are known. As a result, we can reliably discern which folds are involved and how they rotate during each shape transformation.

Specifically, we exemplify the shape morphing of the level-2 structure depicted in Fig. 3a, selecting representative shape transformations to validate the aforementioned theory. Illustrated in Fig. 3a (ii) and Supplementary Fig. 9, we elucidate the theory through the shape morphing processes situated in shape morphing loop 1, namely, from configuration $\mathbf{M}_{\mathrm{A}}$ to configuration $\mathbf{M}_{\mathrm{E}}$.

As shown in Supplementary Fig. 10a-(i), we firstly define the Cartesian coordinate systems fixed on the bottom surface center of the initial configuration $\mathbf{M}_{\mathrm{A}}$. We label each cube with ordered number (1 to 32) to track them during the whole shape morphing process, see the labeled cubes in Supplementary Fig. 10a. According to the equation (S2), we can explicitly and uniquely describe the initial shape by the shape matrix $\mathbf{M}_{\mathrm{A}}$ as

$$\mathbf{M}_{\mathbf{A}} = (\boldsymbol{v}_1, \boldsymbol{v}_2, \boldsymbol{v}_3, \cdots, \boldsymbol{v}_{32})$$

$$= \begin{pmatrix} \begin{pmatrix} -7 \\ -3 \\ 1 \end{pmatrix}, \begin{pmatrix} -7 \\ -1 \\ 1 \end{pmatrix}, \begin{pmatrix} -7 \\ 1 \\ 1 \end{pmatrix}, \begin{pmatrix} -7 \\ 3 \\ 1 \end{pmatrix}, \begin{pmatrix} -5 \\ 3 \\ 1 \end{pmatrix}, \begin{pmatrix} -5 \\ 1 \\ 1 \end{pmatrix}, \begin{pmatrix} -5 \\ -1 \\ 1 \end{pmatrix}, \begin{pmatrix} -5 \\ -3 \\ 1 \end{pmatrix}, \begin{pmatrix} -3 \\ -3 \\ 1 \end{pmatrix}, \begin{pmatrix} -3 \\ -1 \\ 1 \end{pmatrix}, \begin{pmatrix} -3 \\ 1 \\ 1 \end{pmatrix}, \begin{pmatrix} -3 \\ 3 \\ 1 \end{pmatrix}, \begin{pmatrix} -1 \\ 3 \\ 1 \end{pmatrix}, \begin{pmatrix} -1 \\ 1 \\ 1 \end{pmatrix}, \begin{pmatrix} -1 \\ -1 \\ 1 \end{pmatrix}, \begin{pmatrix} -1 \\ -3 \\ 1 \end{pmatrix}, \\ \begin{pmatrix} 1 \\ -3 \\ 1 \end{pmatrix}, \begin{pmatrix} 1 \\ -1 \\ 1 \end{pmatrix}, \begin{pmatrix} 1 \\ 1 \\ 1 \end{pmatrix}, \begin{pmatrix} 1 \\ 3 \\ 1 \end{pmatrix}, \begin{pmatrix} 3 \\ 3 \\ 1 \end{pmatrix}, \begin{pmatrix} 3 \\ 1 \\ 1 \end{pmatrix}, \begin{pmatrix} 3 \\ -1 \\ 1 \end{pmatrix}, \begin{pmatrix} 3 \\ -3 \\ 1 \end{pmatrix}, \begin{pmatrix} 5 \\ -3 \\ 1 \end{pmatrix}, \begin{pmatrix} 5 \\ -1 \\ 1 \end{pmatrix}, \begin{pmatrix} 5 \\ 1 \\ 1 \end{pmatrix}, \begin{pmatrix} 5 \\ 3 \\ 1 \end{pmatrix}, \begin{pmatrix} 7 \\ 3 \\ 1 \end{pmatrix}, \begin{pmatrix} 7 \\ 1 \\ 1 \end{pmatrix}, \begin{pmatrix} 7 \\ -1 \\ 1 \end{pmatrix}, \begin{pmatrix} 7 \\ -3 \\ 1 \end{pmatrix} \end{pmatrix} \quad \text{(S6)}$$

During the shape transformation process from configuration $\mathbf{M}_{\mathrm{A}}$ to configuration $\mathbf{M}_{\mathrm{B}}$, two rotations around *x*-axis (90 degree) with contrary directions exist for each cube. From equation (S4), we know the new spatial position of each cube $\boldsymbol{v}_{k\_new}$ in $\mathbf{M}_{\mathrm{B}}$ can be determined as

$$
\begin{aligned}
\boldsymbol{v}_{k\_new} &= \mathbf{t}_x\left(\theta_x = 90^\circ\right)\mathbf{t}_x\left(\theta_x = -90^\circ\right)\boldsymbol{v}_k \\
&= \begin{bmatrix} 1 & 0 & 0 \\ 0 & \cos\left(90^\circ\right) & \sin\left(90^\circ\right) \\ 0 & -\sin\left(90^\circ\right) & \cos\left(90^\circ\right) \end{bmatrix} \begin{bmatrix} 1 & 0 & 0 \\ 0 & \cos\left(-90^\circ\right) & \sin\left(-90^\circ\right) \\ 0 & -\sin\left(-90^\circ\right) & \cos\left(-90^\circ\right) \end{bmatrix} \boldsymbol{v}_k \\
&= \boldsymbol{v}_k
\end{aligned} \tag{S7}
$$

Therefore, in terms of shape description, we know that $\mathbf{M}_{\mathrm{A}}=\mathbf{M}_{\mathrm{B}}$ (see details in Supplementary Fig. 10a, ii) with

$$
\begin{aligned}
&\mathbf{M}_{\mathbf{B}} = \left(\boldsymbol{v}_1, \boldsymbol{v}_2, \boldsymbol{v}_3, \cdots, \boldsymbol{v}_{32}\right) \\
&= \left(\begin{matrix}
\begin{pmatrix} -7 \\ -3 \\ 1 \end{pmatrix}, \begin{pmatrix} -7 \\ -1 \\ 1 \end{pmatrix}, \begin{pmatrix} -7 \\ 1 \\ 1 \end{pmatrix}, \begin{pmatrix} -7 \\ 3 \\ 1 \end{pmatrix}, \begin{pmatrix} -5 \\ 3 \\ 1 \end{pmatrix}, \begin{pmatrix} -5 \\ 1 \\ 1 \end{pmatrix}, \begin{pmatrix} -5 \\ -1 \\ 1 \end{pmatrix}, \begin{pmatrix} -5 \\ -3 \\ 1 \end{pmatrix}, \begin{pmatrix} -3 \\ -3 \\ 1 \end{pmatrix}, \begin{pmatrix} -3 \\ -1 \\ 1 \end{pmatrix}, \begin{pmatrix} -3 \\ 1 \\ 1 \end{pmatrix}, \begin{pmatrix} -3 \\ 3 \\ 1 \end{pmatrix}, \begin{pmatrix} -1 \\ 3 \\ 1 \end{pmatrix}, \begin{pmatrix} -1 \\ 1 \\ 1 \end{pmatrix}, \begin{pmatrix} -1 \\ -1 \\ 1 \end{pmatrix}, \begin{pmatrix} -1 \\ -3 \\ 1 \end{pmatrix}, \\
\begin{pmatrix} 1 \\ -3 \\ 1 \end{pmatrix}, \begin{pmatrix} 1 \\ -1 \\ 1 \end{pmatrix}, \begin{pmatrix} 1 \\ 1 \\ 1 \end{pmatrix}, \begin{pmatrix} 1 \\ 3 \\ 1 \end{pmatrix}, \begin{pmatrix} 3 \\ 3 \\ 1 \end{pmatrix}, \begin{pmatrix} 3 \\ 1 \\ 1 \end{pmatrix}, \begin{pmatrix} 3 \\ -1 \\ 1 \end{pmatrix}, \begin{pmatrix} 3 \\ -3 \\ 1 \end{pmatrix}, \begin{pmatrix} 5 \\ -3 \\ 1 \end{pmatrix}, \begin{pmatrix} 5 \\ -1 \\ 1 \end{pmatrix}, \begin{pmatrix} 5 \\ 1 \\ 1 \end{pmatrix}, \begin{pmatrix} 5 \\ 3 \\ 1 \end{pmatrix}, \begin{pmatrix} 7 \\ 3 \\ 1 \end{pmatrix}, \begin{pmatrix} 7 \\ 1 \\ 1 \end{pmatrix}, \begin{pmatrix} 7 \\ -1 \\ 1 \end{pmatrix}, \begin{pmatrix} 7 \\ -3 \\ 1 \end{pmatrix}
\end{matrix}\right)
\end{aligned} \tag{S8}
$$

For the shape morphing from configuration $\mathbf{M}_{\mathrm{B}}$ to configuration $\mathbf{M}_{\mathrm{C}}$, some local Cartesian coordinate systems are built at each fold to more mathematically calculate the relative spatial rotations of local cube structural components. For example, we construct the local Cartesian coordinate system at the fold between cube #27 and #30. Then, we can feasibly calculate the new coordinates of the cube #30 under the coordinate system at fixed coordinate systems based on the following rule with a generalized mathermatical form as

$$
\boldsymbol{v}_{k\_new} = \mathbf{t}\boldsymbol{v}_{k\_local} + \boldsymbol{d} \tag{S9}
$$

where vector $\boldsymbol{d}$ is the translational distance between the fixed coordinate systems and the local coordinate systems with

$$
\boldsymbol{d} = \boldsymbol{r}_x + \boldsymbol{r}_y + \boldsymbol{r}_z \tag{S10}
$$

where $\boldsymbol{r}_x$, $\boldsymbol{r}_y$ and $\boldsymbol{r}_z$ separately represents the directional translations shown as Supplementary Fig. 10b; $\boldsymbol{v}_{k\_local}$ is the coordinates of the body center of the rotated cube structural component which can be determined as

$$
\boldsymbol{v}_{k\_local} = \boldsymbol{v}_{k\_old} - \boldsymbol{d} \tag{S11}
$$

For the cube #30, comparing the fixed and the local coordinate systems shown as Supplementary Fig. 10a, ii and iii, we have $\boldsymbol{r}_x = (6, 0, 2)^T$ and $\boldsymbol{r}_y = \boldsymbol{r}_z = \mathbf{0}$. Thus from equation (S10) we have

$$
\boldsymbol{d} = \left(6, 0, 2\right)^T \tag{S12}
$$

Moreover, from equation (S7), we know that the coordinates of cube #30 in configuration $\mathbf{M}_{\mathrm{B}}$ is $\boldsymbol{v}_{30\_old} = (7, 1, 1)$. Thus, from equations (S11) and (S12), we determine the coordinates of the cube #30 in the local coordinate systems as

$$
\boldsymbol{v}_{30\_local}^{\mathbf{M}_{\mathrm{B}}} = \left(7,1,1\right)^T - \left(6,0,2\right)^T = \left(1,1,-1\right)^T \tag{S13}
$$

Given the cube #30 performs the y-direction rotation about 180 degree, we thus can obtain its new coordinates by using equations (S5), (S9) and (S10) with results as

$$\boldsymbol{v}_{30\_new}^{\mathbf{M}_\mathrm{C}} = \begin{bmatrix} \cos\left(180^\circ\right) & 0 & -\sin\left(180^\circ\right) \\ 0 & 1 & 0 \\ \sin\left(180^\circ\right) & 0 & \cos\left(180^\circ\right) \end{bmatrix} \begin{pmatrix} 1 \\ 1 \\ -1 \end{pmatrix} + \begin{pmatrix} 6 \\ 0 \\ 2 \end{pmatrix} = \left(5,1,3\right)^T \tag{S14}$$

To validate the above result, we construct the same coordinate systems as the fixed coordinate systems illustrated as Supplementary Fig. 10a(iii) and obtain the coordinates of cube #30 as

$$\boldsymbol{v}_{30}^{\mathbf{M}_\mathrm{C}} = \left(5,1,3\right)^T \tag{S15}$$

Comparing equations (S12) and (S13) gives

$$\boldsymbol{v}_{30\_new}^{\mathbf{M}_\mathrm{C}} = \boldsymbol{v}_{30}^{\mathbf{M}_\mathrm{C}} \tag{S16}$$

Therefore, we can conclude that the theoretical framework given in equations (S4), (S5), (S9) to (S11) can rationally describe the mathematical operations of shape morphing process between any two mutually transformable configurations.

Consequentially, we can get the shape matrix $\mathbf{M}_\mathrm{C}$ transformed from $\mathbf{M}_\mathrm{B}$ as

$$\begin{aligned} \mathbf{M_C} &= \left(\boldsymbol{v}_1, \boldsymbol{v}_2, \boldsymbol{v}_3, \cdots, \boldsymbol{v}_{32}\right) \\ &= \left(\begin{matrix} \begin{pmatrix}-5\\-3\\3\end{pmatrix}, \begin{pmatrix}-5\\-1\\3\end{pmatrix}, \begin{pmatrix}-5\\1\\3\end{pmatrix}, \begin{pmatrix}-5\\3\\3\end{pmatrix}, \begin{pmatrix}-5\\3\\1\end{pmatrix}, \begin{pmatrix}-5\\1\\1\end{pmatrix}, \begin{pmatrix}-5\\-1\\1\end{pmatrix}, \begin{pmatrix}-5\\-3\\1\end{pmatrix}, \begin{pmatrix}-3\\-3\\1\end{pmatrix}, \begin{pmatrix}-3\\-1\\1\end{pmatrix}, \begin{pmatrix}-3\\1\\1\end{pmatrix}, \begin{pmatrix}-3\\3\\1\end{pmatrix}, \begin{pmatrix}-1\\3\\1\end{pmatrix}, \begin{pmatrix}-1\\1\\1\end{pmatrix}, \begin{pmatrix}-1\\-1\\1\end{pmatrix}, \begin{pmatrix}-1\\-3\\1\end{pmatrix}, \\ \begin{pmatrix}1\\-3\\1\end{pmatrix}, \begin{pmatrix}1\\-1\\1\end{pmatrix}, \begin{pmatrix}1\\1\\1\end{pmatrix}, \begin{pmatrix}1\\3\\1\end{pmatrix}, \begin{pmatrix}3\\3\\1\end{pmatrix}, \begin{pmatrix}3\\1\\1\end{pmatrix}, \begin{pmatrix}3\\-1\\1\end{pmatrix}, \begin{pmatrix}3\\-3\\1\end{pmatrix}, \begin{pmatrix}5\\-3\\1\end{pmatrix}, \begin{pmatrix}5\\-1\\1\end{pmatrix}, \begin{pmatrix}5\\1\\1\end{pmatrix}, \begin{pmatrix}5\\3\\1\end{pmatrix}, \begin{pmatrix}5\\3\\3\end{pmatrix}, \begin{pmatrix}5\\1\\3\end{pmatrix}, \begin{pmatrix}5\\-1\\3\end{pmatrix}, \begin{pmatrix}5\\-3\\3\end{pmatrix} \end{matrix}\right) \end{aligned} \tag{S17}$$

and similarly the shape matrix $\mathbf{M}_\mathrm{D}$, $\mathbf{M}_\mathrm{E}$ and $\mathbf{M}_\mathrm{F}$ as

$$\begin{aligned} \mathbf{M_D} &= \left(\boldsymbol{v}_1, \boldsymbol{v}_2, \boldsymbol{v}_3, \cdots, \boldsymbol{v}_{32}\right) \\ &= \left(\begin{matrix} \begin{pmatrix}-1\\-3\\7\end{pmatrix}, \begin{pmatrix}-1\\-1\\7\end{pmatrix}, \begin{pmatrix}-1\\1\\7\end{pmatrix}, \begin{pmatrix}-1\\3\\7\end{pmatrix}, \begin{pmatrix}-3\\3\\5\end{pmatrix}, \begin{pmatrix}-3\\1\\5\end{pmatrix}, \begin{pmatrix}-3\\-1\\5\end{pmatrix}, \begin{pmatrix}-3\\-3\\5\end{pmatrix}, \begin{pmatrix}-3\\-3\\3\end{pmatrix}, \begin{pmatrix}-3\\-1\\3\end{pmatrix}, \begin{pmatrix}-3\\1\\3\end{pmatrix}, \begin{pmatrix}-3\\3\\3\end{pmatrix}, \begin{pmatrix}-1\\3\\1\end{pmatrix}, \begin{pmatrix}-1\\1\\1\end{pmatrix}, \begin{pmatrix}-1\\-1\\1\end{pmatrix}, \begin{pmatrix}-1\\-3\\1\end{pmatrix}, \\ \begin{pmatrix}1\\-3\\1\end{pmatrix}, \begin{pmatrix}1\\-1\\1\end{pmatrix}, \begin{pmatrix}1\\1\\1\end{pmatrix}, \begin{pmatrix}1\\3\\1\end{pmatrix}, \begin{pmatrix}3\\3\\3\end{pmatrix}, \begin{pmatrix}3\\1\\3\end{pmatrix}, \begin{pmatrix}3\\-1\\3\end{pmatrix}, \begin{pmatrix}3\\-3\\3\end{pmatrix}, \begin{pmatrix}3\\-3\\5\end{pmatrix}, \begin{pmatrix}3\\-1\\5\end{pmatrix}, \begin{pmatrix}3\\1\\5\end{pmatrix}, \begin{pmatrix}3\\3\\5\end{pmatrix}, \begin{pmatrix}1\\3\\7\end{pmatrix}, \begin{pmatrix}1\\1\\7\end{pmatrix}, \begin{pmatrix}1\\-1\\7\end{pmatrix}, \begin{pmatrix}1\\-3\\7\end{pmatrix} \end{matrix}\right) \\ \mathbf{M_E} &= \left(\boldsymbol{v}_1, \boldsymbol{v}_2, \boldsymbol{v}_3, \cdots, \boldsymbol{v}_{32}\right) \\ &= \left(\begin{matrix} \begin{pmatrix}-1\\-7\\3\end{pmatrix}, \begin{pmatrix}-1\\-5\\5\end{pmatrix}, \begin{pmatrix}-1\\5\\5\end{pmatrix}, \begin{pmatrix}-1\\7\\3\end{pmatrix}, \begin{pmatrix}-3\\7\\-1\end{pmatrix}, \begin{pmatrix}-3\\3\\5\end{pmatrix}, \begin{pmatrix}-3\\-3\\5\end{pmatrix}, \begin{pmatrix}-3\\-7\\-1\end{pmatrix}, \begin{pmatrix}-3\\-5\\-1\end{pmatrix}, \begin{pmatrix}-3\\-1\\3\end{pmatrix}, \begin{pmatrix}-3\\1\\3\end{pmatrix}, \begin{pmatrix}-3\\5\\-1\end{pmatrix}, \begin{pmatrix}-1\\3\\-1\end{pmatrix}, \begin{pmatrix}-1\\1\\1\end{pmatrix}, \begin{pmatrix}-1\\-1\\1\end{pmatrix}, \begin{pmatrix}-1\\-3\\-1\end{pmatrix}, \\ \begin{pmatrix}1\\-3\\-1\end{pmatrix}, \begin{pmatrix}1\\-1\\1\end{pmatrix}, \begin{pmatrix}1\\1\\1\end{pmatrix}, \begin{pmatrix}1\\3\\-1\end{pmatrix}, \begin{pmatrix}3\\5\\-1\end{pmatrix}, \begin{pmatrix}3\\1\\3\end{pmatrix}, \begin{pmatrix}3\\-1\\3\end{pmatrix}, \begin{pmatrix}3\\-5\\-1\end{pmatrix}, \begin{pmatrix}3\\-7\\1\end{pmatrix}, \begin{pmatrix}3\\-3\\5\end{pmatrix}, \begin{pmatrix}3\\3\\5\end{pmatrix}, \begin{pmatrix}3\\7\\1\end{pmatrix}, \begin{pmatrix}1\\7\\3\end{pmatrix}, \begin{pmatrix}1\\5\\5\end{pmatrix}, \begin{pmatrix}1\\-5\\5\end{pmatrix}, \begin{pmatrix}1\\-7\\3\end{pmatrix} \end{matrix}\right) \\ \mathbf{M_F} &= \left(\boldsymbol{v}_1, \boldsymbol{v}_2, \boldsymbol{v}_3, \cdots, \boldsymbol{v}_{32}\right) \\ &= \left(\begin{matrix} \begin{pmatrix}-5\\-3\\3\end{pmatrix}, \begin{pmatrix}-5\\-1\\3\end{pmatrix}, \begin{pmatrix}-5\\1\\3\end{pmatrix}, \begin{pmatrix}-5\\3\\3\end{pmatrix}, \begin{pmatrix}-3\\7\\3\end{pmatrix}, \begin{pmatrix}-5\\1\\1\end{pmatrix}, \begin{pmatrix}-5\\-1\\1\end{pmatrix}, \begin{pmatrix}-3\\-7\\3\end{pmatrix}, \begin{pmatrix}-5\\-5\\3\end{pmatrix}, \begin{pmatrix}-3\\-1\\1\end{pmatrix}, \begin{pmatrix}-3\\1\\1\end{pmatrix}, \begin{pmatrix}-5\\5\\3\end{pmatrix}, \begin{pmatrix}-5\\3\\1\end{pmatrix}, \begin{pmatrix}-3\\1\\1\end{pmatrix}, \begin{pmatrix}-3\\-1\\1\end{pmatrix}, \begin{pmatrix}-5\\-3\\1\end{pmatrix}, \\ \begin{pmatrix}5\\-3\\1\end{pmatrix}, \begin{pmatrix}3\\-1\\1\end{pmatrix}, \begin{pmatrix}3\\1\\1\end{pmatrix}, \begin{pmatrix}5\\3\\1\end{pmatrix}, \begin{pmatrix}5\\5\\3\end{pmatrix}, \begin{pmatrix}3\\1\\1\end{pmatrix}, \begin{pmatrix}3\\-1\\1\end{pmatrix}, \begin{pmatrix}3\\-5\\3\end{pmatrix}, \begin{pmatrix}3\\-7\\3\end{pmatrix}, \begin{pmatrix}5\\-1\\1\end{pmatrix}, \begin{pmatrix}5\\1\\1\end{pmatrix}, \begin{pmatrix}3\\7\\1\end{pmatrix}, \begin{pmatrix}5\\3\\3\end{pmatrix}, \begin{pmatrix}5\\1\\3\end{pmatrix}, \begin{pmatrix}5\\-1\\3\end{pmatrix}, \begin{pmatrix}5\\1\\3\end{pmatrix} \end{matrix}\right) \end{aligned} \tag{S18}$$

Particularly, for the cube components with multiple directional rotations, two cases should be considered:

Case 1: multiple directional rotations in same fold (see Supplementary Fig. 10d). In this case, we can rewrite equation (S8) by combining with equation (S5) as

$$\boldsymbol{v}_{k\_new} = \left(\prod_1^m \mathbf{t}_m\right)\boldsymbol{v}_{k\_local} + \boldsymbol{d} \tag{S19}$$

where m can be 1, 2 or 3, and represent the number of its directional rotations.

Case 2: the most general case, i.e., multiple directional rotations at different folds (see Supplementary Fig. 10e). By equations (S5) and (S19), we can determine the new coordinates of certain cube components in new reconfigured configurations as

$$\boldsymbol{v}_{k\_new} = \left(\prod_1^n \mathbf{t}_n\right)\mathbf{F}_{t-1}\left(\mathbf{t}, \boldsymbol{v}_{k\_local}, \boldsymbol{d}_{t-1}\right) + \boldsymbol{d}_t \tag{S20}$$

with the function $\mathbf{F}_{t\text{-}1}$ is an multiply iteration vector function with $\mathbf{F}_1 = \left(\prod_1^m \mathbf{t}_m^1\right)\boldsymbol{v}_{k\_local} + \boldsymbol{d}_1$ and $\mathbf{F}_2 = \left(\prod_1^n \mathbf{t}_n^2\right)\left[\left(\prod_1^m \mathbf{t}_m^1\right)\boldsymbol{v}_{k\_local} + \boldsymbol{d}_1\right] + \boldsymbol{d}_2$ (the superscript in the rotation matrix representing different folds with total number as t, t is integer and $t \leq 32$). Moreover, for the shape morphing processes involving with multiple folds whose rotation angles (i.e., the reconfiguration kinematics) are related, the rotation angle relations will needed to be firstly derived. And we present the theoretical frame work to determine reconfiguration kinematics in Note 7.

Besides from the reconfiguration loop 1, we also use the above theoretical framework to analyze the shape morphing process on the branch from configuration $\mathbf{M}_B$ to $\mathbf{M}_{19}$ bypassing configuration $\mathbf{M}_8$ and configuration $\mathbf{M}_{14}$, see Supplementary Fig. 10c. Starting from equation (S8) and based on equations (S4), (S5), (S8) to (S10) and (S19), we can derive the explicit form of the shape matrix of configurations $\mathbf{M}_8$, $\mathbf{M}_{14}$ and $\mathbf{M}_{19}$ as

$$\mathbf{M}_8 = \left(\boldsymbol{v}_1, \boldsymbol{v}_2, \boldsymbol{v}_3, \cdots, \boldsymbol{v}_{32}\right)$$
$$= \begin{pmatrix} \begin{pmatrix}-7\\-3\\1\end{pmatrix}, \begin{pmatrix}-7\\-1\\1\end{pmatrix}, \begin{pmatrix}-7\\1\\3\end{pmatrix}, \begin{pmatrix}-7\\3\\3\end{pmatrix}, \begin{pmatrix}-5\\3\\3\end{pmatrix}, \begin{pmatrix}-5\\1\\3\end{pmatrix}, \begin{pmatrix}-5\\-1\\1\end{pmatrix}, \begin{pmatrix}-5\\-3\\1\end{pmatrix}, \begin{pmatrix}-3\\-3\\1\end{pmatrix}, \begin{pmatrix}-3\\-1\\1\end{pmatrix}, \begin{pmatrix}-3\\1\\3\end{pmatrix}, \begin{pmatrix}-3\\3\\3\end{pmatrix}, \begin{pmatrix}-1\\3\\3\end{pmatrix}, \begin{pmatrix}-1\\1\\3\end{pmatrix}, \begin{pmatrix}-1\\-1\\1\end{pmatrix}, \begin{pmatrix}-1\\-3\\1\end{pmatrix}, \\ \begin{pmatrix}1\\-3\\1\end{pmatrix}, \begin{pmatrix}1\\-1\\1\end{pmatrix}, \begin{pmatrix}1\\1\\3\end{pmatrix}, \begin{pmatrix}1\\3\\3\end{pmatrix}, \begin{pmatrix}3\\3\\3\end{pmatrix}, \begin{pmatrix}3\\1\\3\end{pmatrix}, \begin{pmatrix}3\\-1\\1\end{pmatrix}, \begin{pmatrix}3\\-3\\1\end{pmatrix}, \begin{pmatrix}5\\-3\\1\end{pmatrix}, \begin{pmatrix}5\\-1\\1\end{pmatrix}, \begin{pmatrix}5\\1\\3\end{pmatrix}, \begin{pmatrix}5\\3\\3\end{pmatrix}, \begin{pmatrix}7\\3\\3\end{pmatrix}, \begin{pmatrix}7\\1\\3\end{pmatrix}, \begin{pmatrix}7\\-1\\1\end{pmatrix}, \begin{pmatrix}7\\-3\\1\end{pmatrix} \end{pmatrix}$$

$$\mathbf{M}_{14} = \left(\boldsymbol{v}_1, \boldsymbol{v}_2, \boldsymbol{v}_3, \cdots, \boldsymbol{v}_{32}\right)$$
$$= \begin{pmatrix} \begin{pmatrix}-7\\-3\\-1\end{pmatrix}, \begin{pmatrix}-7\\-1\\-1\end{pmatrix}, \begin{pmatrix}-5\\1\\3\end{pmatrix}, \begin{pmatrix}-5\\3\\3\end{pmatrix}, \begin{pmatrix}-5\\3\\1\end{pmatrix}, \begin{pmatrix}-5\\1\\1\end{pmatrix}, \begin{pmatrix}-5\\-1\\1\end{pmatrix}, \begin{pmatrix}-5\\-3\\1\end{pmatrix}, \begin{pmatrix}-3\\-3\\1\end{pmatrix}, \begin{pmatrix}-3\\-1\\1\end{pmatrix}, \begin{pmatrix}-3\\1\\3\end{pmatrix}, \begin{pmatrix}-3\\3\\3\end{pmatrix}, \begin{pmatrix}-1\\3\\3\end{pmatrix}, \begin{pmatrix}-1\\1\\3\end{pmatrix}, \begin{pmatrix}-1\\-1\\1\end{pmatrix}, \begin{pmatrix}-1\\-3\\1\end{pmatrix}, \\ \begin{pmatrix}1\\-3\\1\end{pmatrix}, \begin{pmatrix}1\\-1\\1\end{pmatrix}, \begin{pmatrix}1\\1\\3\end{pmatrix}, \begin{pmatrix}1\\3\\3\end{pmatrix}, \begin{pmatrix}3\\3\\3\end{pmatrix}, \begin{pmatrix}3\\1\\3\end{pmatrix}, \begin{pmatrix}3\\-1\\1\end{pmatrix}, \begin{pmatrix}3\\-3\\1\end{pmatrix}, \begin{pmatrix}5\\-3\\1\end{pmatrix}, \begin{pmatrix}5\\-1\\1\end{pmatrix}, \begin{pmatrix}5\\1\\1\end{pmatrix}, \begin{pmatrix}5\\3\\1\end{pmatrix}, \begin{pmatrix}7\\3\\-1\end{pmatrix}, \begin{pmatrix}7\\1\\-1\end{pmatrix}, \begin{pmatrix}7\\-1\\-1\end{pmatrix}, \begin{pmatrix}7\\-3\\-1\end{pmatrix} \end{pmatrix}$$

$$\mathbf{M}_{19} = \left(\boldsymbol{v}_1, \boldsymbol{v}_2, \boldsymbol{v}_3, \cdots, \boldsymbol{v}_{32}\right)$$
$$= \begin{pmatrix} \begin{pmatrix}-3\\-3\\-5\end{pmatrix}, \begin{pmatrix}-3\\-1\\-5\end{pmatrix}, \begin{pmatrix}-1\\1\\-5\end{pmatrix}, \begin{pmatrix}-1\\3\\-5\end{pmatrix}, \begin{pmatrix}-1\\3\\-3\end{pmatrix}, \begin{pmatrix}-1\\1\\-3\end{pmatrix}, \begin{pmatrix}-3\\-1\\-3\end{pmatrix}, \begin{pmatrix}-3\\-3\\-3\end{pmatrix}, \begin{pmatrix}-3\\-3\\-1\end{pmatrix}, \begin{pmatrix}-3\\-1\\-1\end{pmatrix}, \begin{pmatrix}-1\\1\\-1\end{pmatrix}, \begin{pmatrix}-1\\3\\-1\end{pmatrix}, \begin{pmatrix}-1\\3\\1\end{pmatrix}, \begin{pmatrix}-1\\1\\1\end{pmatrix}, \begin{pmatrix}-1\\-1\\1\end{pmatrix}, \begin{pmatrix}-1\\-3\\1\end{pmatrix}, \\ \begin{pmatrix}1\\-3\\1\end{pmatrix}, \begin{pmatrix}1\\-1\\1\end{pmatrix}, \begin{pmatrix}1\\1\\1\end{pmatrix}, \begin{pmatrix}1\\3\\1\end{pmatrix}, \begin{pmatrix}3\\3\\-1\end{pmatrix}, \begin{pmatrix}3\\1\\-1\end{pmatrix}, \begin{pmatrix}1\\-1\\-1\end{pmatrix}, \begin{pmatrix}1\\-3\\-1\end{pmatrix}, \begin{pmatrix}1\\-3\\-3\end{pmatrix}, \begin{pmatrix}1\\-1\\-3\end{pmatrix}, \begin{pmatrix}3\\1\\-3\end{pmatrix}, \begin{pmatrix}3\\3\\-3\end{pmatrix}, \begin{pmatrix}3\\3\\-5\end{pmatrix}, \begin{pmatrix}3\\1\\-5\end{pmatrix}, \begin{pmatrix}1\\-1\\-5\end{pmatrix}, \begin{pmatrix}1\\-3\\-5\end{pmatrix} \end{pmatrix} \tag{S21}$$

It should be noted that the theoretical framework outlined above is based on the forward shape morphing of a single structure. Consequently, we can determine the ultimate shape matrix $\mathbf{M}_{\text{final}}$ for each branch. Ultimately, the final configuration should have no forward reconfiguration degrees of freedom. Thus, the rotation matrix in equation (S5) will be with form as

$$\mathbf{t}_x = \mathbf{t}_y = \mathbf{t}_z = \mathbf{0} \tag{S22}$$

Moreover, for the shape morphing branch with reconfiguration loop, the final shape matrix $\mathbf{M}_{\text{final}}$ can be feasibly decided when the rotation matrix **t** repeatedly shows up.

Furthermore, distinguishing the initial shape solely based on the shape matrix M is challenging, as the initial shape of different designs within each leveled structure shares an identical structural form. Given that the primary distinction among specific structures within each level design lies in the relative spatial positions of hinges, we adopt a similar strategy in constructing the shape matrix here, focusing on accurately discerning the initial shape through the hinges' position matrix with mathematical forma as $\mathbf{H} = (\boldsymbol{h}_1, \boldsymbol{h}_2, \ldots, \boldsymbol{h}_\text{k})$ (k is the hinge quantity, and $\boldsymbol{h}_k$ is the position vector of the center of each hinge).

For example, based on the fixed global coordinate system and the cube labeling method defined in Supplementary Fig. 10a, we can get the hinge position matrix of the <8R, 4R> type of level-2 structure shown in Fig. 3a as

$$\mathbf{H}_1^2 = (\boldsymbol{v}_1, \boldsymbol{v}_2, \boldsymbol{v}_3, \cdots, \boldsymbol{v}_{36})$$

$$= \left(\begin{matrix} \begin{pmatrix}-7\\-2\\2\end{pmatrix} & \begin{pmatrix}-7\\0\\0\end{pmatrix} & \begin{pmatrix}7\\2\\2\end{pmatrix} & \begin{pmatrix}-6\\4\\1\end{pmatrix} & \begin{pmatrix}-6\\0\\1\end{pmatrix} & \begin{pmatrix}-6\\0\\1\end{pmatrix} & \begin{pmatrix}-6\\-4\\1\end{pmatrix} & \begin{pmatrix}-4\\-3\\2\end{pmatrix} & \begin{pmatrix}-4\\-1\\2\end{pmatrix} & \begin{pmatrix}-4\\1\\2\end{pmatrix} & \begin{pmatrix}-4\\3\\2\end{pmatrix} & \begin{pmatrix}-2\\4\\1\end{pmatrix} & \begin{pmatrix}-2\\0\\1\end{pmatrix} & \begin{pmatrix}-2\\0\\1\end{pmatrix} & \begin{pmatrix}-2\\-4\\1\end{pmatrix} & \begin{pmatrix}-1\\-2\\2\end{pmatrix} & \begin{pmatrix}1\\2\\2\end{pmatrix} & \begin{pmatrix}0\\3\\2\end{pmatrix} \\ \begin{pmatrix}0\\-3\\2\end{pmatrix} & \begin{pmatrix}1\\-2\\2\end{pmatrix} & \begin{pmatrix}1\\2\\2\end{pmatrix} & \begin{pmatrix}2\\4\\1\end{pmatrix} & \begin{pmatrix}2\\0\\1\end{pmatrix} & \begin{pmatrix}2\\0\\1\end{pmatrix} & \begin{pmatrix}2\\-4\\1\end{pmatrix} & \begin{pmatrix}4\\-3\\2\end{pmatrix} & \begin{pmatrix}4\\-1\\2\end{pmatrix} & \begin{pmatrix}4\\1\\2\end{pmatrix} & \begin{pmatrix}4\\3\\2\end{pmatrix} & \begin{pmatrix}6\\4\\1\end{pmatrix} & \begin{pmatrix}6\\0\\1\end{pmatrix} & \begin{pmatrix}6\\0\\1\end{pmatrix} & \begin{pmatrix}6\\-4\\1\end{pmatrix} & \begin{pmatrix}7\\-2\\2\end{pmatrix} & \begin{pmatrix}7\\0\\0\end{pmatrix} & \begin{pmatrix}7\\2\\2\end{pmatrix} \end{matrix}\right)$$

with the superscript “2” representing as structural level. Similarly, the combinatorically designed level-2 structure by changing the structural symmetries of level 1 hinges shown in Fig. 3d, i has form as

$$\mathbf{H}_2^2 = (\boldsymbol{v}_1, \boldsymbol{v}_2, \boldsymbol{v}_3, \cdots, \boldsymbol{v}_{36})$$

$$= \left(\begin{matrix} \begin{pmatrix}-7\\-2\\2\end{pmatrix} & \begin{pmatrix}-7\\0\\0\end{pmatrix} & \begin{pmatrix}7\\2\\2\end{pmatrix} & \begin{pmatrix}-6\\4\\1\end{pmatrix} & \begin{pmatrix}-6\\0\\1\end{pmatrix} & \begin{pmatrix}-6\\0\\1\end{pmatrix} & \begin{pmatrix}-6\\-4\\1\end{pmatrix} & \begin{pmatrix}-4\\-3\\2\end{pmatrix} & \begin{pmatrix}-4\\-1\\2\end{pmatrix} & \begin{pmatrix}-4\\1\\2\end{pmatrix} & \begin{pmatrix}-4\\3\\2\end{pmatrix} & \begin{pmatrix}-2\\4\\1\end{pmatrix} & \begin{pmatrix}\mathbf{-2}\\\mathbf{0}\\\mathbf{1}\end{pmatrix} & \begin{pmatrix}\mathbf{-2}\\\mathbf{-1}\\\mathbf{0}\end{pmatrix} & \begin{pmatrix}-2\\1\\0\end{pmatrix} & \begin{pmatrix}-1\\-2\\2\end{pmatrix} & \begin{pmatrix}1\\2\\2\end{pmatrix} & \begin{pmatrix}0\\3\\2\end{pmatrix} \\ \begin{pmatrix}0\\-3\\2\end{pmatrix} & \begin{pmatrix}1\\-2\\2\end{pmatrix} & \begin{pmatrix}1\\2\\2\end{pmatrix} & \begin{pmatrix}2\\4\\1\end{pmatrix} & \begin{pmatrix}\mathbf{2}\\\mathbf{1}\\\mathbf{0}\end{pmatrix} & \begin{pmatrix}\mathbf{2}\\\mathbf{-1}\\\mathbf{0}\end{pmatrix} & \begin{pmatrix}2\\-4\\1\end{pmatrix} & \begin{pmatrix}4\\-3\\2\end{pmatrix} & \begin{pmatrix}4\\-1\\2\end{pmatrix} & \begin{pmatrix}4\\1\\2\end{pmatrix} & \begin{pmatrix}4\\3\\2\end{pmatrix} & \begin{pmatrix}6\\4\\1\end{pmatrix} & \begin{pmatrix}6\\0\\1\end{pmatrix} & \begin{pmatrix}6\\0\\1\end{pmatrix} & \begin{pmatrix}6\\-4\\1\end{pmatrix} & \begin{pmatrix}7\\-2\\2\end{pmatrix} & \begin{pmatrix}7\\0\\0\end{pmatrix} & \begin{pmatrix}7\\2\\2\end{pmatrix} \end{matrix}\right)$$

wherein the spatial vectors of the position changed level-1 hinges have been highlighted with bond font.

Supplementary Note 5. Demonstration of the flexible length of level-2 links

The continuous shape-changing capabilities of our designed hierarchical structure are directly influenced by the flexible lengths of the links within higher-level structures. For simplicity, we adopt the distance between the centers of two adjacent level-2 line folds as the measure of the level-2 link length. Under the fixed global coordinate system, we can precisely determine the spatial coordinates of the center of level-2 folds. Specifically, we represent them using four spatial vectors as $\boldsymbol{V}_m$ with m = 1 to 4. Consequently, we can establish our defined length of two adjacent level-2 line folds as

$$d = \left|\boldsymbol{V}_{m+1} - \boldsymbol{V}_m\right| \tag{S23}$$

Based on the equation (S23), we have derived the varying level-2 lengths and present the result in Fig. 3b. Moreover, shown as Supplementary Fig. 11, based on two continuously reconfigured shapes, i.e. shape $\mathbf{M}_4$ to shape $\mathbf{M}_{10}$ displayed in Fig. 3a, ii, we simply calculated and compared the link length changes from level-2 fold B11 to fold T21’ and from level-2 fold B11’ to fold T21’ with results shown in Supplementary Fig. 11b.

We show that the length of the shape changed links keep varying with different values (see the black line in Supplementary Fig. 11b) while the link lengths of non-reconfigured part remain constant (see the green line in Supplementary Fig. 11b).

Supplementary Note 6. Reconfiguration DOFs of the selected level-2 structure in Fig. 3

While our proposed hierarchical structures can produce a vast array of complex configurations, it's noteworthy that the reconfiguration degrees of freedom (DOFs) for each shape-changing process consistently remain relatively low, typically not exceeding three. This observation is exemplified by the analyzed level-1 and level-2 structures depicted in Fig. 3a, where we monitored the reconfiguration DOFs for selected shape-changing processes and concurrently compared them with their corresponding rotated folds numbers.

Based on the Denavit-Hartbenberg theorem, i.e.,

$$\prod_{m=1}^{8}\mathbf{T}_m = \mathbf{I} \tag{S24}$$

we can calculate the number of reconfiguration DOFs for all the reconfiguration processes of the 8R level-1 structure (see details in Supplementary Fig. 7b-7c), which is no larger than two. The shape-changing processes involving two reconfiguration degrees of freedom (DOFs) in the 8R level-1 structure consist of the chain-like reconfiguration process, where the structure remains compact, and the fully opening process as an 8R looped mechanism. However, upon integration of the level-1 structure into the level-2 structures as flexible links, the reconfiguration DOFs of its 8R reconfiguration path automatically reduce to one. This reduction occurs due to the constraints arising from the structural symmetries of the level-2 structures.

Taking these considerations into account, we monitored the reconfiguration degrees of freedom (DOFs) for several representative shape-changing processes of the level-2 structures depicted in Fig. 3a and summarized the results in Supplementary Fig. 14. It is evident that the number of rotated folds for all selected reconfiguration processes (with a maximum of 18 folds) is consistently much less than the total number of level-2 folds (36). Furthermore, the corresponding reconfiguration DOFs for each process are significantly smaller than the rotated folds number. For instance, the reconfiguration processes from shape $\mathbf{M}_7$ to $\mathbf{M}_{13}$ and from shape $\mathbf{M}_{13}$ to $\mathbf{M}_{18}$, both of which contain 18 rotated folds, have only one DOF (Supplementary Fig. 14, A and B).

We note that the maximum reconfiguration DOFs for all selected reconfiguration processes is three, while most processes have just one DOF. Additionally, we analyzed the combinatorically designed level-2 system shown in Fig. 3d and found that the reconfiguration DOFs for all selected shape-changing processes are one (Supplementary Fig. 15). This low reconfiguration DOF is attributed to the internal fold interconnectivity induced by the 8R and 4R looped-mechanism-like folds of the level-1 and level-2 structures.

Supplementary Note 7. Reconfiguration kinematic analysis of level-1 and level-2 structures

Supplementary Note 7.1 Reconfiguration kinematics by analyzing the relations of opening angles

We observe that the transitional geometric kinematics of all hierarchical structures depend on the kinematics of level-1 structures. As all level-1 systems are based on looped bar-link mechanisms, we can theoretically determine their kinematics using the Denavit-Hartenberg theorem with equation (S24). Illustrated by Fig. 3a, we understand that the kinematic bifurcation of both level-1 and level-2 structures plays important roles in developing more configuration paths and thus reconfigured shapes. Employing a linear-perturbation method based on equation (S24), we can

numerically track the occurrence of kinematic bifurcations. The analytical details are described below.

The level-1 and level-2 structures depicted in Fig. 3a are chosen here to illustrate the derivation details. Regarding the level-1 structure, when symmetrically deployed as an 8R bar-link looped mechanism-like system (Path-2 shown in Supplementary Fig. 7b), and based on the constructed local coordinate systems shown in Supplementary Fig. 16, it exhibits only one reconfiguration degree of freedom (DOF), with the relationships of the 8 opening angles as follows:

$$\begin{aligned}&\gamma_{k2}=\gamma_{k4}=\gamma_{k6}=\gamma_{k8},\gamma_{k1}=\gamma_{k3}=\gamma_{k5}=\gamma_{k7}\\&\gamma_{k1}=\sin^{-1}\left[\left(1-\cos\gamma_{k2}\right)\left(1+\cos\gamma_{k2}\right)^{-1}\right]\end{aligned}\tag{S25}$$

wherein $0 \le \gamma_{k2} \le 180^o$ and $1 \le k \le 4$ is integer representing the four links of level-2 structures. And when the level-1 system is deployed as a 6R looped-mechanism like system (Path-3 shown as Supplementary Fig. 7b), for example with $\gamma_{k1} \equiv \gamma_{k5} \equiv 0$, the relation of the opening angles has form as

$$\begin{aligned}&\gamma_{k2}\equiv\gamma_{k6}=\gamma_{k4}\equiv\gamma_{k8},\ \gamma_{k5}\equiv\gamma_{k1},\gamma_{k3}\equiv\gamma_{k7}=0\\&\gamma_{k1}=\sin^{-1}\left[\sin^2\gamma_{k2}\left(1+\cos^2\gamma_{k2}\right)^{-1}\right]\end{aligned}\tag{S26}$$

when $0^o \le \gamma_{k2} \le 90^o$; For $90^o \le \gamma_{k2} \le 180^o$, Equation. (S3) can be rewritten as

$$\begin{aligned}&\gamma_{k2}\equiv\gamma_{k6}=\gamma_{k4}\equiv\gamma_{k8},\gamma_{k5}\equiv\gamma_{k1},\gamma_{k3}\equiv\gamma_{k7}=0\\&\gamma_{k1}=180^\circ-\sin^{-1}\left[\sin^2\gamma_{k2}\left(1+\cos^2\gamma_{k2}\right)^{-1}\right]\end{aligned}\tag{S27}$$

Note that the above kinematics are suitable for level-1 system reconfigure starting from the initial shapes with all opening angles as 0°. In fact, for the reconfigured shapes mutually transformable with opening angles in different links as n × 90°, the level-1 structure links are always in different reconfigured states from the initial configuration state. We present one example in Supplementary Fig. 17 with γ_{k1} and $\gamma_{k5} = 90^o$, which corresponds to the level-2 reconfiguration process from shape $\mathbf{M}_A$ to shape $\mathbf{M}_F$ in Fig. 3a. We find that the angle relations in this process also follow the rules in equation (S26). In fact, the opening angles γ_{k1} and γ_{k5} can be randomly selected between 0° and 90°, such as γ_0 ($0^o \le \gamma_0 \le 90^o$) while the maximum value of the opening angles γ_{k2}, γ_{k4}, γ_{k6} and γ_{k8} are limited to

$$\begin{aligned}&\left(\gamma_{k2}\right)_{\max}=\left(\gamma_{k6}\right)_{\max}=\left(\gamma_{k4}\right)_{\max}=\left(\gamma_{k8}\right)_{\max}\\&\left(\gamma_{k2}\right)_{\max}=\cos^{-1}\left[\left(1-\sin\gamma_{k1}\right)/\left(1+\sin\gamma_{k1}\right)\right]^{0.5}\end{aligned}\tag{S28}$$

with three cases with $\gamma_{k1} = \gamma_{k5} = 30^o$, 60° and 90° are plotted in Supplementary Fig. 17.

Moreover, we note that sequential deployment can as well occur for the level-1 structure. For example, the level-1 links involved in the reconfiguration process from shape $\mathbf{M}_7$ to shape $\mathbf{M}_{13}$ can firstly change the opening angles γ_{k2}, γ_{k4}, γ_{k6} and γ_{k8} to a certain value, such as γ_{k0} with $0^o \le \gamma_{k0} \le 90^o$. Then, the opening angles γ_{k1}, γ_{k3}, γ_{k5} and γ_{k7} can be linearly increased by following equation (S25) until a limited value as

$$\begin{aligned}&\gamma_{k1}=\gamma_{k3}=\gamma_{k5}=\gamma_{k7}\\&\left(\gamma_{k1}\right)_{Limited}=\sin^{-1}\left[\left(1-\cos\gamma_{k0}\right)\left(1+\cos\gamma_{k0}\right)^{-1}\right]\end{aligned}\tag{S29}$$

Lastly, the eight angles can open simultaneously by following equation (S25) to deploy the level-1 structure as an 8R-looped mechanism, see the demonstration with physical prototypes in Supplementary Fig. 17a**,** i.

By setting the initial values of opening angles γ_{k2}, γ_{k4}, γ_{k6} and γ_{k8} to 90º, 110º, 130º, 150º and 170º, we plot the variations of the opening angles of the reconfigured level-1 link in Supplementary Fig. 17b, ii.

Further, based on equations (S25) to (S27), we also analyze some other reconfiguration processes listed in Fig. 3a as shown in Supplementary Fig. 16, for example, the processes from shape $\mathbf{M}_{14}$ and to shape $\mathbf{M}_{20}$, from shape $\mathbf{M}_{14}$ to $\mathbf{M}_{29}$, and the whole reconfiguration loop $\mathbf{M}_A$-$\mathbf{M}_B$-$\mathbf{M}_C$-$\mathbf{M}_D$-$\mathbf{M}_E$-$\mathbf{M}_F$-$\mathbf{M}_A$. We find that all the three reconfiguration processes exhibit rather simple linear or nonlinear but controllable kinematics for both level-1 and level-2 folds. The simple transition kinematics, from the control aspect, also benefit our hierarchical design concept being more advantageous than previous designs with complex or tedious paths.

Supplementary Note 7.2 Kinematic bifurcation analysis

As explained in the main text, kinematic bifurcation enables the branched shape changes in both level-1 and level-2 structures. In the following, we numerically evaluate the occurrence of kinematic bifurcations for the level-1 links and the level-2 structure in Fig. 3a. Based on the singular value decomposition (SVD) method of the Jacobian matrix, we can firstly decompose the transformation matrix **T** into translational and rotational parts, i.e.,

$$\boldsymbol{T}_k = \boldsymbol{T}_k^L \boldsymbol{T}_k^\gamma \tag{S30}$$

with

$$\begin{aligned} \left[\boldsymbol{T}_k^L\right] &= \begin{bmatrix} 1 & 0 & 0 & -a_k \\ 0 & \cos\alpha_k & \sin\alpha_k & -s_k\sin\alpha_k \\ 0 & -\sin\alpha_k & \cos\alpha_k & -s_k\cos\alpha_k \\ 0 & 0 & 0 & 1 \end{bmatrix} \\ \left[\boldsymbol{T}_k^\gamma\right] &= \begin{bmatrix} \cos\gamma_k & \sin\gamma_k & 0 & 0 \\ -\sin\gamma_k & \cos\gamma_k & 0 & 0 \\ 0 & 0 & 1 & 0 \\ 0 & 0 & 0 & 1 \end{bmatrix} \end{aligned} \tag{S31}$$

wherein α_k is the angle between the successive hinge axis z_i and hinge axis z_{i+1} positively with right hand rules in the positive direction of axis x_{i+1}; a_k is the perpendicular distance between the hinge axis z_i and hinge axis z_{i+1}; γ_k is the angle between the link bar axis of *i*(*i*+1), i.e., x_i and the link axis of *i*(*i*+1), i.e., x_{i+1}, positively along the positive direction of axis z_i with right hand rule; s_k the perpendicular distance between the *x*-axes. And all these geometrical parameters can be determined easily according to the local coordinate systems built in Supplementary Fig. 16.

Then, a linear perturbation method can be used by increasing the opening angle γ_k with an infinitesimal incremental, i.e., $\delta\gamma_k$. Substituting the varied opening angle, i.e., $\gamma_k + \delta\gamma_k$, into equation (S31) gives

$$\left[\boldsymbol{T}_k^{\gamma_k+\delta\gamma_k}\right] = [\boldsymbol{T}_k^{\gamma_k}] + [\boldsymbol{T}_k^{\gamma_k}]'\delta\gamma_k \tag{S32}$$

with

$$[\boldsymbol{T}_k^{\gamma_k}]' = \left[\frac{\partial \boldsymbol{T}_k^{\gamma_k}}{\partial \gamma_k}\right] = \begin{bmatrix} -\sin\gamma_k & \cos\gamma_k & 0 & 0 \\ -\cos\gamma_k & -\sin\gamma_k & 0 & 0 \\ 0 & 0 & 1 & 0 \\ 0 & 0 & 0 & 1 \end{bmatrix} \quad \text{(S33)}$$

Combining Equations (S31) to (S33) with equation (S24) can give

$$\prod_{k=1}^{4\ or\ 8} \boldsymbol{T}_k^L \boldsymbol{T}_k^{\gamma_k} + \sum_{k=1}^{4\ or\ 8} \boldsymbol{M}_k \delta\gamma_k = \boldsymbol{I} \quad \text{(S34)}$$

wherein "8" represent the 8R looped-mechanism like level-1 structure and "4" is for the 4R looped-mechanism like level-2 structure.

Thus, for level-1 structure with 8 links, from equation (S34), we have

$$\sum_{k=1}^{8} \boldsymbol{M}_k \delta\gamma_k = \left(\prod_{k=1}^{8} \boldsymbol{T}_k^L [\boldsymbol{T}_k^{\gamma_k}]'\right)\delta\gamma_1 + \cdots + \left(\prod_{k=1}^{8} \boldsymbol{T}_k^L [\boldsymbol{T}_k^{\gamma_k}]'\right)\delta\gamma_8 \quad \text{(S35)}$$

, meanwhile for level-2 structure, we have

$$\sum_{k=1}^{4} \boldsymbol{M}_k \delta\gamma_k^2 = \left(\prod_{k=1}^{4} \boldsymbol{T}_k^L [\boldsymbol{T}_k^{\gamma_k^2}]'\right)\delta\gamma_1^2 + \cdots + \left(\prod_{k=1}^{4} \boldsymbol{T}_k^L [\boldsymbol{T}_k^{\gamma_k^2}]'\right)\delta\gamma_4^2 \quad \text{(S36)}$$

wherein the superscript ^{2} represent the second hierarchy level.

Finally, decomposing the coefficient matrix in Equations (S35) and (S36) by singular value method can determine the occurrence of kinematic bifurcation for both level-1 and level-2 structures with respect to their opening angles. Generally, the SVD method gives

$$\mathbf{M} = \mathbf{UVW}^{\mathrm{T}} \quad \text{(S37)}$$

Wherein for the level-1 structure, **U** and **W** are respective 6×6 and 8×8 square orthogonal matrices containing left- and right-singular vectors, while **V** is a 6×8 rectangular matrix with r non-zero singular values on its main diagonal and r is the rank of the matrix **M**; for the level-2 structure, **U**, **V**, **W** are all respective 4×4 matrices.

Physically, the matrix **V** for level-1 structure with a 6×8 form stands for the multiple structural DOFs. However, as abovementioned, one of the structural mobility is constrained by the structural symmetry, which reduces the level-1 structure with just one DOF when there is no occurrence of kinematic bifurcation. If kinematic bifurcation occurs, the structural DOFs will suddenly increase to be larger than one at a unique opening angle, which is equal to m×90° (m is integer with m = 0 to 4) for more different shapes. We verified the occurrence of the level-1 structure used in Fig. 3a. From the results shown in the inset in Fig. 3b, we can see that the six singular values that are equal to zero correspond to the occurrence of kinematic bifurcation, where more reconfiguration path branches show up as demonstrated in Supplementary Fig. 7b. Moreover, the structural reconfiguration topologies also change for different bifurcated reconfiguration paths. For example, at the bifurcation configuration state with γ_k = 90°, the level-1 structure can reconfigure from its original 8R-looped mechanism like to 4R looped-mechanism like or 2R chain-like systems (see details in Supplementary Fig. 7b, Path 2).

For the hierarchical level-2 structure, we note the occurrence of its kinematic bifurcation is related to the variations of the distance between level-2 folds induced by the reconfiguration of level-1 links. With some simple geometrical calculations and combining with equations (S24), (S35), and (S36), we can obtain (see the inset in Fig. 3b)

$$v_{44} \equiv 0 \quad \text{(S38)}$$

from where we can conclude that the level-2 structure in Fig. 3a is always reconfigurable with multiple DOFs.

Supplementary Note 8. Quantifying the number of reconfiguration paths of level-2 structure

Based on the theory provided in Note 7.2, we can predict all the locations of kinematic bifurcations of all combinatorically designed level-1 structures, and thus calculate the quantities of all the deployable reconfiguration paths of each level-1 design.

Given the multileveled structural hierarchy features, we note that it is rather complicated to directly predict the exact number of all the reconfiguration paths of level-2 structures by theory since kinematic bifurcations occur in both the level-1 and level-2 links. Instead, here we combine the SVD theoretical method in Note 7.2 and the experimental results to more efficiently calculate almost all the reconfiguration paths. The calculation process is listed as below:

Step-1: Experimentally determine the number of initial reconfiguration path types and also the number of any combinatorically designed level-2 structure;

Step-2: For each reconfiguration path achieved in Step-1, when the level-1 and level-2 rotation angle equal to times of 90 degree, we use the SVD method for both the level-1 and level-2 links to calculate their singular values v_{kk} (k_{max} = 6 for level-1 link while equal to 4 for level-2 link). If v_{kk} = 0 for level-1 and/or level-2 link, we know that the structure can morph further whose compatible type and quantity need to be experimentally determined. Meanwhile, we monitored all the reconfiguration paths without kinematic bifurcation yet possess multiple DOFs.

Step-3: Repeat step-1 and 2 and summarize all the achieved kinematic bifurcation points by building the data tree shown in Fig. 3a and Supplementary Fig. 9 until there is no further deployable paths.

Based on the above method, we can mathematically express all the achievable reconfiguration paths as

$$N = \sum_{k=1}^{p} N_k + \sum_{k=1}^{q} m_{k+1} M_k + \sum_{k=1}^{r} r_{k+1} R_k \tag{S39}$$

wherein N_k denotes the number of configuration state at level-k of the date tree; M_k demotes the number of bifurcated configuration at level-*k* of the data tree while m_k as its branched paths at the level-(*k*+1) of the data tree; R_k denotes the number of configurations without kinematic bifurcation but with multiple DoFs while r_{k+1} as its combinatorically deployable paths.

Based Equation. (S39), we determined all the data points in Fig. 3c.

Supplementary Note 9. Untethered and remotely controlled designs

To demonstrate the capabilities of the system to reconfigure autonomously, we built a robotic prototype for both the level-1 and level-2 mechanisms, see details in Supplementary Fig. 19a-19b.

The modules are fabricated in polylactic acid (PLA) via fused deposition modeling (FDM), using the QIDI Tech X-Max 3D printer. They consist of the four lateral faces of a cube (81.5 mm side, average mass 40 g), with empty spots to reduce the weight of the structure and to avoid interference during reconfiguration. By exploiting the kinematic redundancy of the mechanisms, only a subset of the joints is actuated. More in detail, the active joints are 5 and 22 for the level-1 and level-2 structures, respectively. The passive joints are implemented as pin joints, while the active ones rely on servomotors for the actuation. The motors are connected to the cubes via u-shaped mounting brackets so that the rotation axis coincides with one edge of the cube. Apposite support structures are integrated into the cubes for the assembly of the motor brackets.

Each motor (DSservo RDS3225) is powered by a 3.7V LiPo battery and controlled via its specific control board (Adafruit ItsyBitsy nRF52840 Express). Additional chips are incorporated for accommodating the JST connector for the battery (Adafruit Pro Trinket LiIon/LiPoly Backpack

Add-On) and for adaption of the supply voltage (SparkFun Logic Level Converter - Bi-Directional). The control boards are identified by a numeric ID and communicate with each other via Bluetooth according to a serial framework where each controller receives the information from the previous one and sends them to the next one. Upstream, an application with a graphical interface has been developed in Mathworks to enable intuitive open-loop position control through either predefined sequences or input commands by the user (see details in Supplementary Fig. 20 and Supplementary Fig. 21).

We show that legged locomotion can be performed by the repetition of an opportune gait cycle. An example is illustrated in Supplementary Fig. 19c, where the gait cycle is divided into four phases and has a total duration of 6s. Supplementary Fig. 19d shows how the locomotion speed is affected by some external conditions, such as the introduction of payloads or the presence of slope. It is worth noting that the implemented gait cycle was empirically chosen to demonstrate the feasibility of the locomotion. Friction is expected to strongly affect the effectiveness of locomotion. Thus, an optimization study would be interesting to enhance the speed performance of the robot in various conditions.

Supplementary Note 10. Structural stability and loading capacity of the reconfigured shapes

In terms of the structural stability, the reconfigured shapes can be classified as two different types. However, we here consider only the case of the hierarchical origami-based structures being with folds with negligible bending stiffness. Therefore, we can conclude that the first type includes the reconfigured shapes that can stand stably when being placed onto ground in natural state with/without constraints from the ground friction force. Reflecting by the reconfigured shapes of the level-2 structure demonstrated in Fig. 3a, we find the reconfigured shapes with structural stability include shapes $\mathbf{M}_B$, $\mathbf{M}_C$, $\mathbf{M}_E$, $\mathbf{M}_3$, $\mathbf{M}_4$, $\mathbf{M}_6$, $\mathbf{M}_7$, $\mathbf{M}_9$, $\mathbf{M}_{10}$, $\mathbf{M}_{11}$, $\mathbf{M}_{12}$, $\mathbf{M}_{14}$, $\mathbf{M}_{15}$, $\mathbf{M}_{16}$, $\mathbf{M}_{18}$, $\mathbf{M}_{19}$, $\mathbf{M}_{22}$, $\mathbf{M}_{24}$. Illustrated by the static force analysis (Supplementary Fig. 23) of two representative stable reconfigured shapes in Fig. 6c, we note that the structural stability of these stable reconfigured shapes are attributed to the constraints from the structural interference among cube-shaped structural components with non-negligible thickness and/or the ground frictions. Shown as static force analysis in Supplementary Fig. 23a-23b**,** (ii), the structural interference between cubes can make the bridge-shaped configuration into an over-constrained structure. Constrained by the ground supporting and friction forces, it can tolerate some external loads and enable the internal folds-based hinges being stretched.

The second type are those that cannot stably hold their reconfigured shapes and will collapse in natural state. Without external locking mechanisms (for example using folds with high non-elastic torsional stiffness to maintain the deformed state), these unstable reconfigured shapes are without force balance and thus will collapse. Reflecting by the reconfigured shapes of the level-2 structure demonstrated in Fig. 3a, the unstable reconfigured shapes include $\mathbf{M}_D$, $\mathbf{M}_F$, $\mathbf{M}_5$, $\mathbf{M}_8$, $\mathbf{M}_{13}$, $\mathbf{M}_{17}$, $\mathbf{M}_{20}$, $\mathbf{M}_{21}$, $\mathbf{M}_{23}$, $\mathbf{M}_{25}$.

Supplementary Note 11. Inverse design of level-2 structure for imitating target shapes

Inverse design to imitate target shapes for special application scenarios can also be accessible for our hierarchical structures. However, the imitating process of our inverse design is different from previous designs by prestigiously presetting unique material/structural patterns to purposely retain the target shapes. Our inverse design method is based on the selection algorithm from the reconfigured shape library by following several steps.

First is to build a database for the configuration library. Each cube can be treated as a spatial pixel with its geometrical center represented by a vector. Then, we can use a matrix to characterize a morphed shape, where the spatial positions of composed cubes are described by their corresponding vectors. For example, for all the combinatorically designed level-2 structures shown in Supplementary Fig. 5, for one special design k, all its reconfigured shapes can be summarized into

$$\left(\mathbf{M}_{\mathrm{k1}},\cdots,\mathbf{M}_{\mathrm{kn}}\right) \tag{S40}$$

where $\mathbf{M}_{\mathrm{kn}}$ represent the mathematically expressed forms of the nth reconfigured shapes in the transition tree for the kth combinatorically designed level-2 structures.

Second is to compose all the combinatorically designed level-2 structures into the database matrix **D** in the form of

$$\mathbf{D}=\begin{pmatrix} \mathbf{M}_{11} & \mathbf{M}_{12} & \cdots & \mathbf{M}_{1i} & 0 & 0 & 0 & 0 & 0 & 0 \\ \mathbf{M}_{21} & \mathbf{M}_{22} & \cdots & \mathbf{M}_{2i} & \cdots & \mathbf{M}_{2j} & 0 & 0 & 0 & 0 \\ \vdots & \vdots & \vdots & \vdots & \vdots & \vdots & \vdots & \vdots & \vdots & \vdots \\ \mathbf{M}_{(k-1)1} & \mathbf{M}_{(k-1)2} & \cdots & \mathbf{M}_{(k-1)i} & \cdots & \mathbf{M}_{(k-1)j} & \cdots & \mathbf{M}_{(k-1)m} & 0 & 0 \\ \mathbf{M}_{k1} & \mathbf{M}_{k2} & \cdots & \mathbf{M}_{ki} & \cdots & \mathbf{M}_{kj} & \cdots & \mathbf{M}_{km} & \cdots & \mathbf{M}_{kz} \end{pmatrix} \tag{S41}$$

where z stands for the maximum number of reconfigured shapes by the kth level-2 structure.

Third is to discretize the target shape into cube-shaped voxelated pixels and mathematically convert it into a mathematical matrix **T**.

Last is to find the shapes in the database that match for the target shape by comparing the matrix **T** with the components of database matrix, i.e., $\mathbf{D}_{\mathrm{ij}}$. There are two criterions to find out the optimal imitated shape: (1) find the smallest value of the error function **Errf** defined as

$$\mathbf{Errf}=\left\|\mathbf{T}-\mathbf{D}_{ij}\right\| / \left\|\mathbf{T}-\mathbf{D}_{ij}\right\|_{\max} \tag{S42}$$

wherein $\| \ \|$ represent the mode of matrix and usually $\left\|\mathbf{T}-\mathbf{D}_{ij}\right\|_{\max}$ is determined as $\|\mathbf{T}\|+\left\|\mathbf{D}_{\mathrm{ij}}\right\|_{\max}$ for simplicity. (2) The conditions that guarantee the imitated shapes whose cube pixels are with approximately the same absolute spatial positions with the target shape, i.e.

$$\left\|\boldsymbol{v}_{\mathbf{T}}-\boldsymbol{v}_{\mathbf{D}_{ij}}\right\|=0 \tag{S43}$$

Finally, we can obtain the most approximately imitated shape $\mathbf{M}_{\mathrm{km}}$ from the database. The inverse design method is briefly summarized in Supplementary Fig. 25.

We validate our inverse design method by imitating a flight-shape robot shown as Supplementary Fig. 26. However, to conduct the second criterion judging the shape limitations without relative errors induced by the coordinate system, we need firstly translate the geometrical center of all the reconfigured shapes to the center of the built Cartesian coordinate systems (8 by 8 by 8, see details in Supplementary Fig. 26**,** a to c). We can finally get the imitated shape from our built database with

$$\left(\mathbf{Errf}\right)_{\min}\approx 5.6\% \tag{S44}$$

while $\left(\mathbf{Errf}\right)_{\max}\approx 50\%$, see the imitated shape in Supplementary Fig. 26d.

Supplementary Note 12. Demonstration of the generality of the proposed hierarchical design principle

In the main text, we successfully demonstrated our proposed design principle by introducing the commonly used structural hierarchy concept to construct hierarchical origami-based meta-

structures. However, we should stress out that our proposed hierarchical design principle is a fundamental and generalizable design scheme, and not restricted to the cube-shaped structural components.

Given the truth that the rigidly foldable origami structures (both the 2D thin sheet and 3D thick panel origami structures) can be equivalently simplified as bar-linkage systems, we therefore can conclude that our proposed hierarchical origami-based meta-structures are free from the geometrical features of the used structural components. Based on the structural motifs in Supplementary Fig. 3a(i) by using four basic bar-linkage rigid kinematic mechanisms, we firstly illustrate the above conclusion by replacing the cube-shaped structural components with prism-shaped one with triangle-shaped cross section. Shown as the simulation results in Supplementary Fig. 27, the newly designed 8R level-1 structure exhibits not only the identical shape morphing features as the original design with cube-shaped structural components, but also generate new shapes which are believed to be induced by the unique structural geometry of the triangular prism. Similarly, based on the hierarchical design principle in Supplementary Fig. 3, we also successfully demonstrate the generalized design possibility of <8R, 4R> type of level-2 structures shown as the representative simulation results in Supplementary Fig. 27b which is based on the <8R> level-1 structure in Supplementary Fig. 27A.

Inspired by the first exploration with triangular prism as structural component, we further illustrate other shape structural components, such as those shown in Supplementary Fig. 28a to S28b by using the thick plate and tetra-decahedron. More generally, we believe our proposed hierarchical design principle is suitable to shaped structural components only if they can form into the compatible structures without self-stress in the initial configuration state.

Moreover, beyond the illustrated designs in main text and the extended cases in Supplementary Fig. 27 and Supplementary Fig. 28a to S28b whose structural forms are only arranged in 2D plane, we note our proposed hierarchical design principle have another generalized design degree of freedom by using the third structural dimensions. We briefly demonstrate this based on the level-1 structure with simplest structural form shown as Supplementary Fig. 28c(i), i.e., the cube shaped <8R> overconstrained structure composed by eight cubes connected with equal number of line folds to form into a loop similar as that shown in Fig. 2a.

Then, according to our proposed the hierarchical design principle, we further construct the <8R, 8R> type of level-2 structure made by 8 <8R> type of level-1 structures connected by 8 level-2 line folds. We present one representative 3D level-2 structure in Supplementary Fig. 28c. By running some simulations, we find the designed 3D level-2 structure not only can change shape with structural form exactly identical as its composed level-1 structures, but also display advancing structural shape morphing features and deploy into more complex structural form shown as Supplementary Fig. 28c, ii. In fact, this 3D formed hierarchical origami-based metastructure can be significantly expanded with a systematic and huge design space. And we leave this to the future explorations.

Therefore, we can conclude that our proposed hierarchical design principle for the construction of hierarchical origami-based metastructures is a universal design scheme which can fundamentally enlarge and rich the origami family.

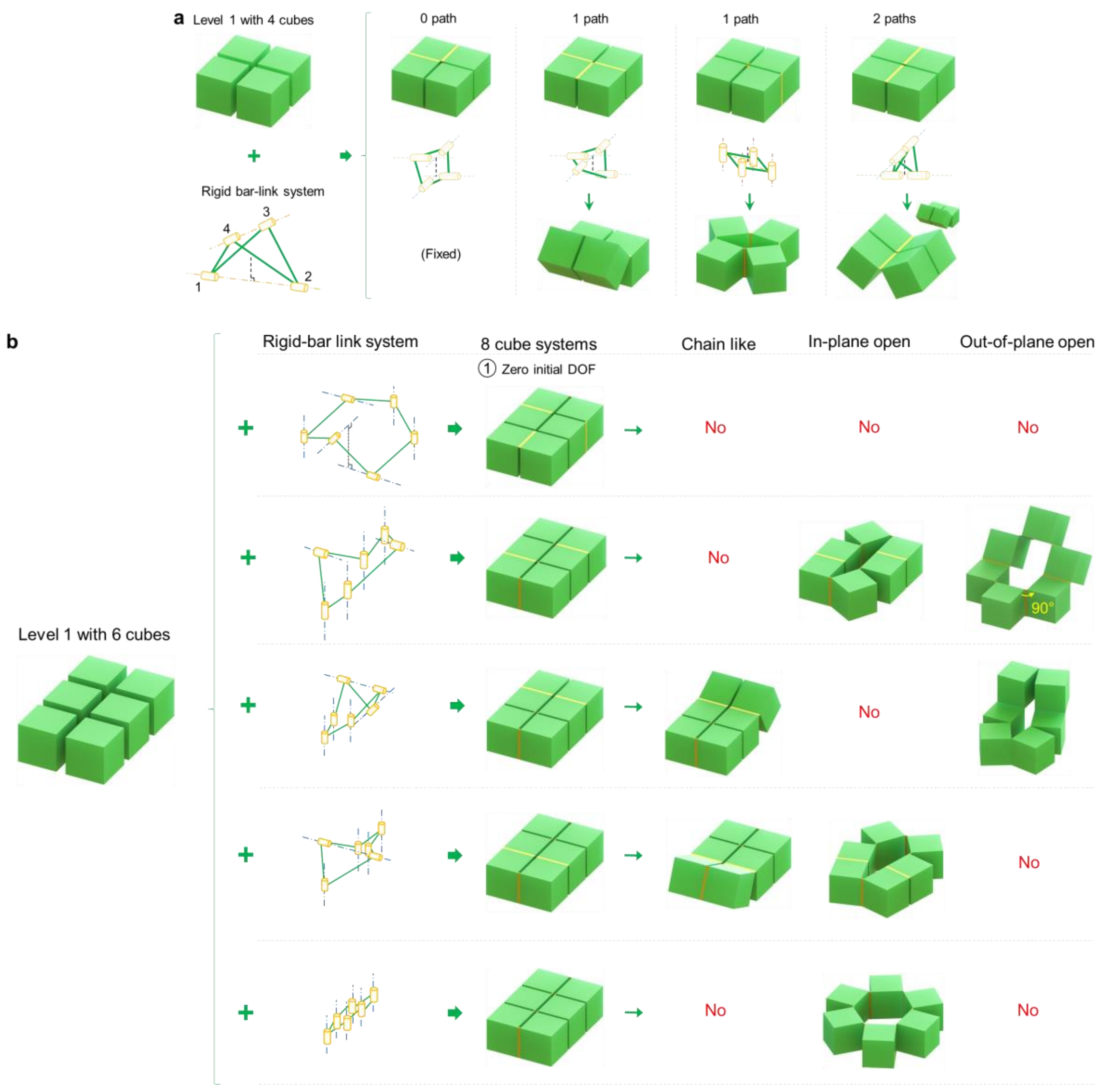
a
Level 1 with 4 cubes
0 path
1 path
1 path
2 paths
Rigid bar-link system
4
3
1
2
(Fixed)
b
Rigid-bar link system
8 cube systems
① Zero initial DOF
Chain like
In-plane open
Out-of-plane open
No
No
No
No
90°
No
No
No
No
Level 1 with 6 cubes

Contd.

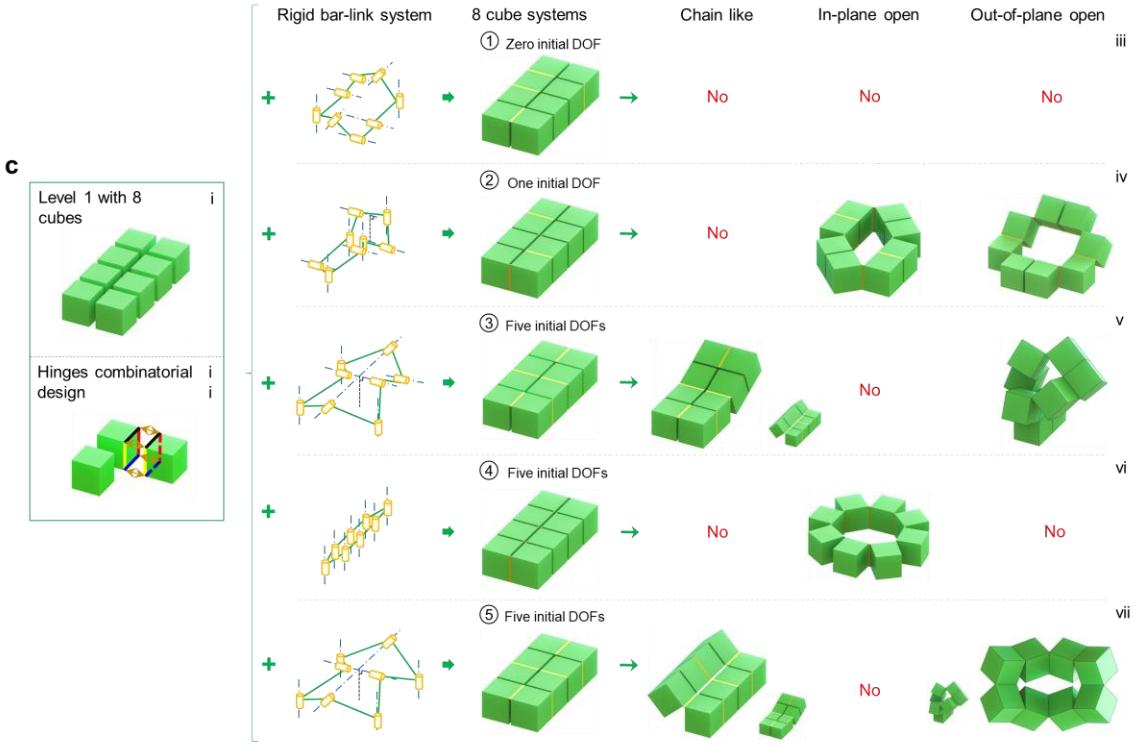

Supplementary Fig. 1. Combinatorial design details of 4, 6 and 8-cube based level-1 structures with different structural DOFs.

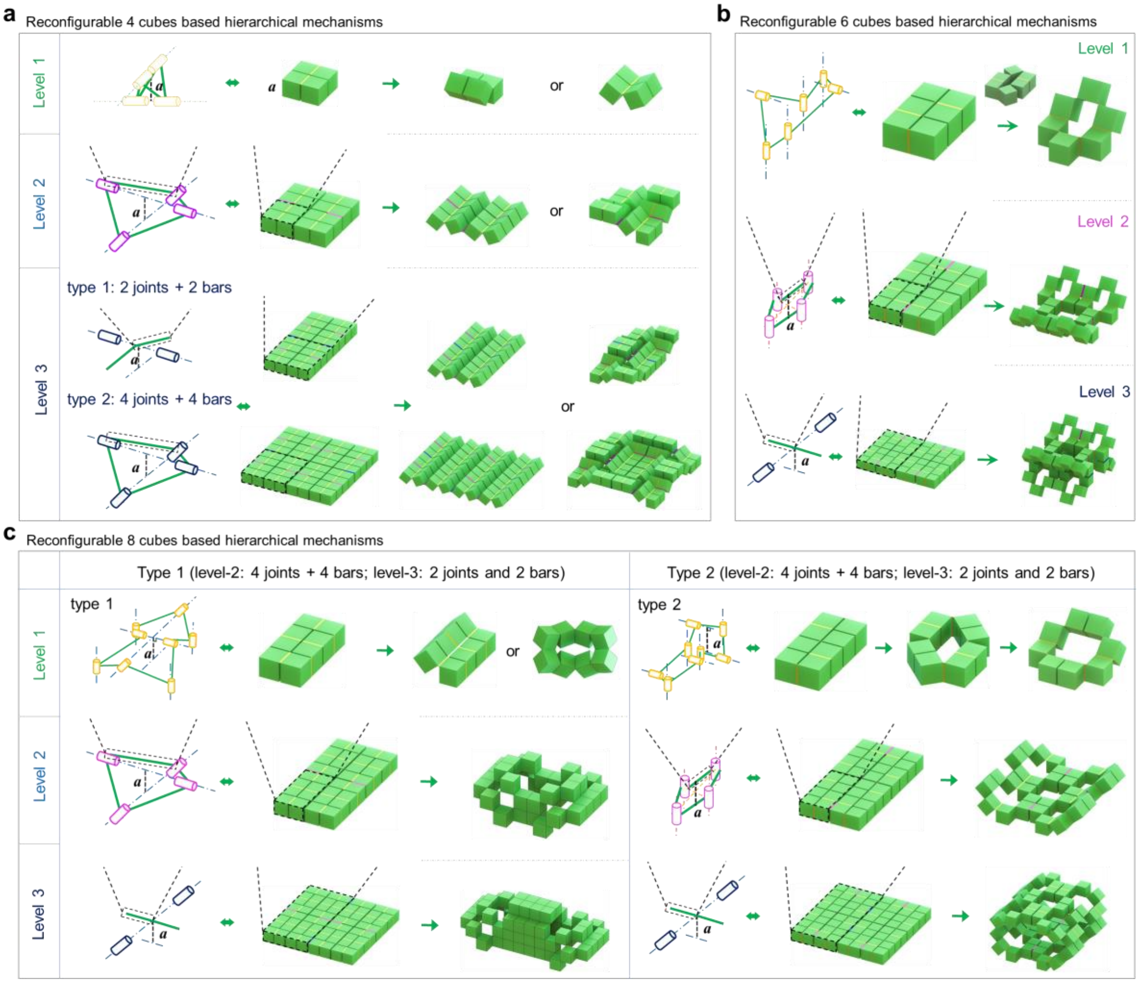

Supplementary Fig. 2. Schematic illustration of the combinatorial design of deformable level-2 systems by using different number of cube-shaped structural components and rearranging the folds positions. a, Design details for <4R, 4R>, <4R, 4R, 2R> and <4R, 4R, 4R> types of level-2, 3 systems. **b**, Design details for <6R, 4R> and <6R, 4R, 2R> type of level-2, 3 systems. **c**, Design details for two <8R, 4R> type of level-2 systems and two <8R, 4R, 2R> type of level-3 systems.

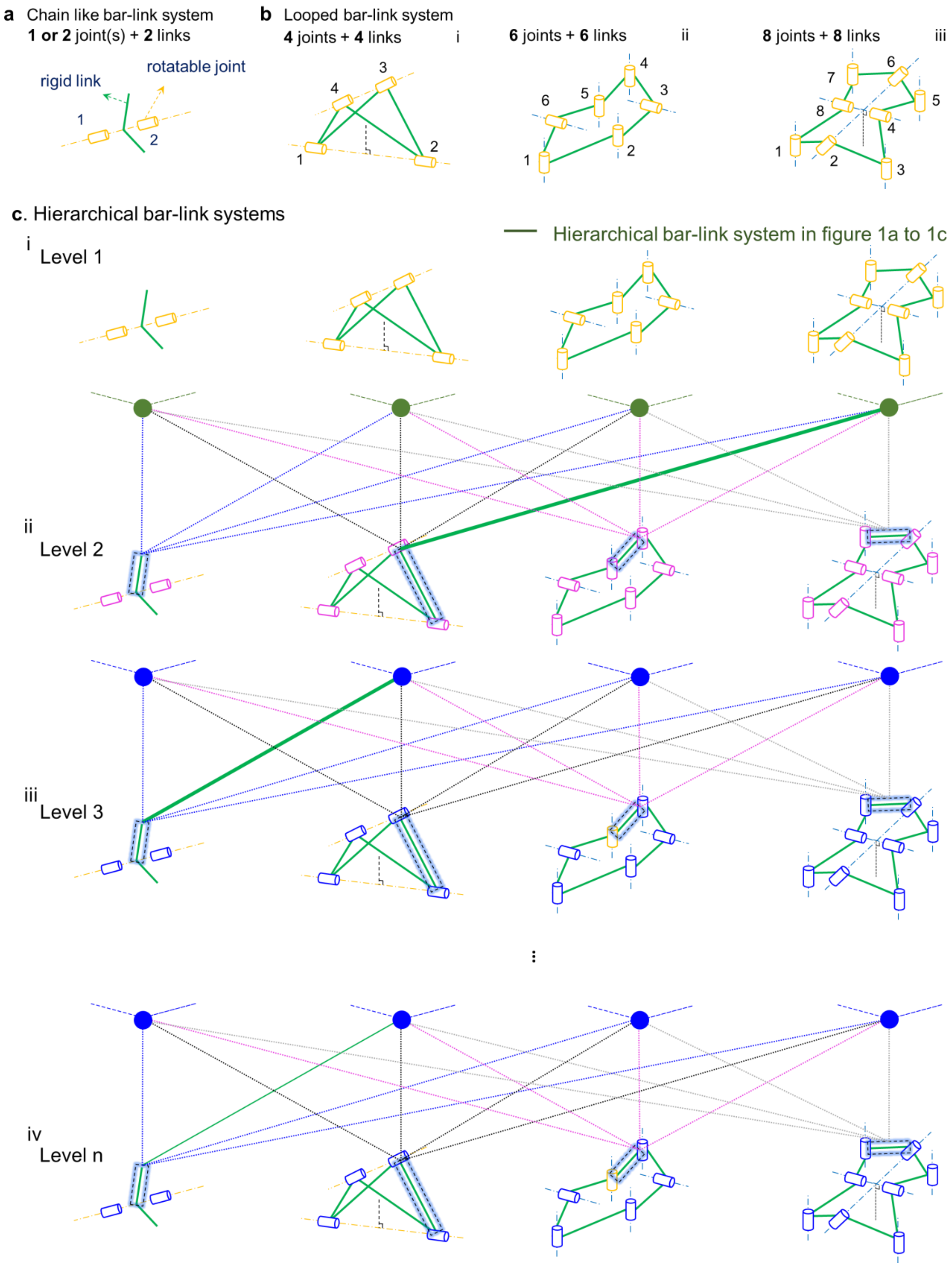

Supplementary Fig. 3. Combinatorial design details of level-1 structures with different structural motifs. a and **b,** Four different types of basic structural motifs for hierarchical uses: the

2R chain-like (a), and three 4R-, 6R-, and 8R-looped bar-link mechanism like structural motif(s) (**b**). (**c**) Schematics of the combinatorial design principle of the hierarchical origami-based metastructures: i, Four different types of rigid bar-linkage kinematic mechanisms as structural motifs: the 2R non-loop rigid kinematic mechanism (two rigid links hinged by one rotatable bar), and the 4R, 6R and 8R looped overconstrained rigid kinematic mechanisms (the quantity of rotating bars is equal to the rigid links; ii-iv Schematics of the construction of higher level n (n ⩾ 2) origami-based metastructures by using the same four structural motifs in level 1 structures and combinatorically replacing the higher level links with lower level structures, see the links highlighted by the rectangular shaped dash lines.

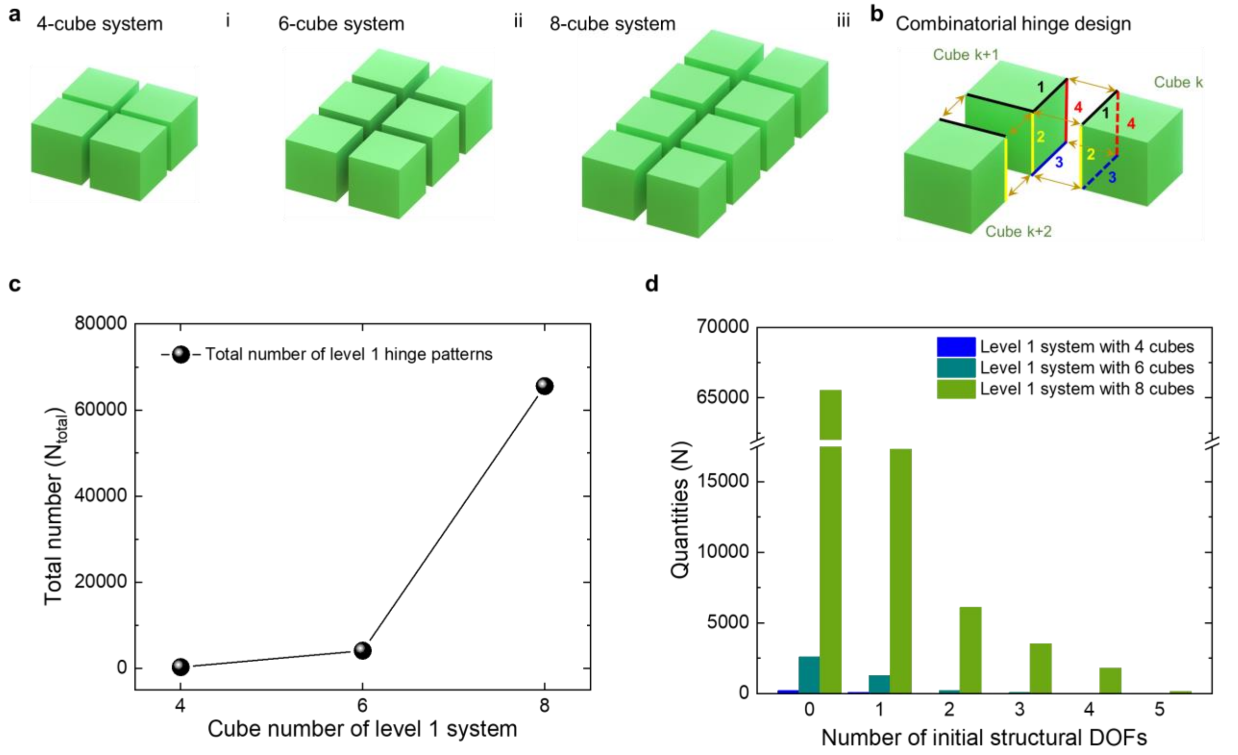

Supplementary Fig. 4. Combinatorial design of level-1 structures with cube shaped structural component. **a**, Combinatorial design by using different number of structural components: from left to right as 4, 6 and 8 cube systems. **b**, Combinatorial design by changing folds positions between two adjacent cubes. **c**, Total number of design possibilities of the 4, 6, and 8 cube level-1 systems by rearranging folds positions. **d**, The number of designs for the three different type of level-1 systems under different structural DOFs.

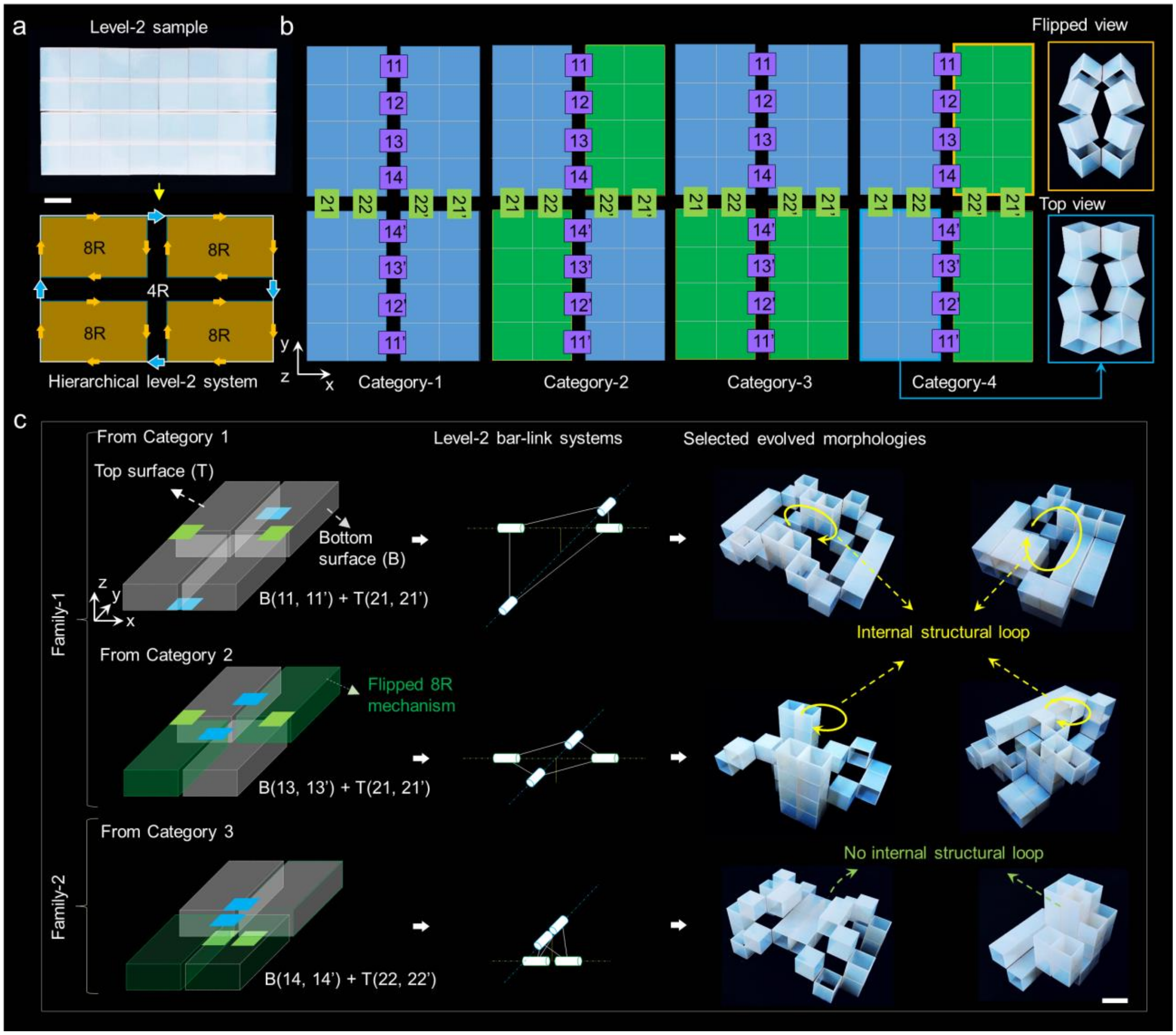

Supplementary Fig. 5. Combinatorial design of <8R, 4R> type of level-2 systems by rearranging the spatial positions of both folds and level-1 system. a, Level-2 sample and the schematic illustration of hierarchical <8R, 4R> type of level-2 folds. **b**, Four different design cases for <8R, 4R> level-2 systems by placing level-1 systems with xz- and yz-plane symmetries, one-fold symmetry, only yz-plane symmetry and xz-plane symmetry. **c**, Two categories of reconfigured level-2 systems: category-1 with internal structural loop and category-2 without internal structural loop.

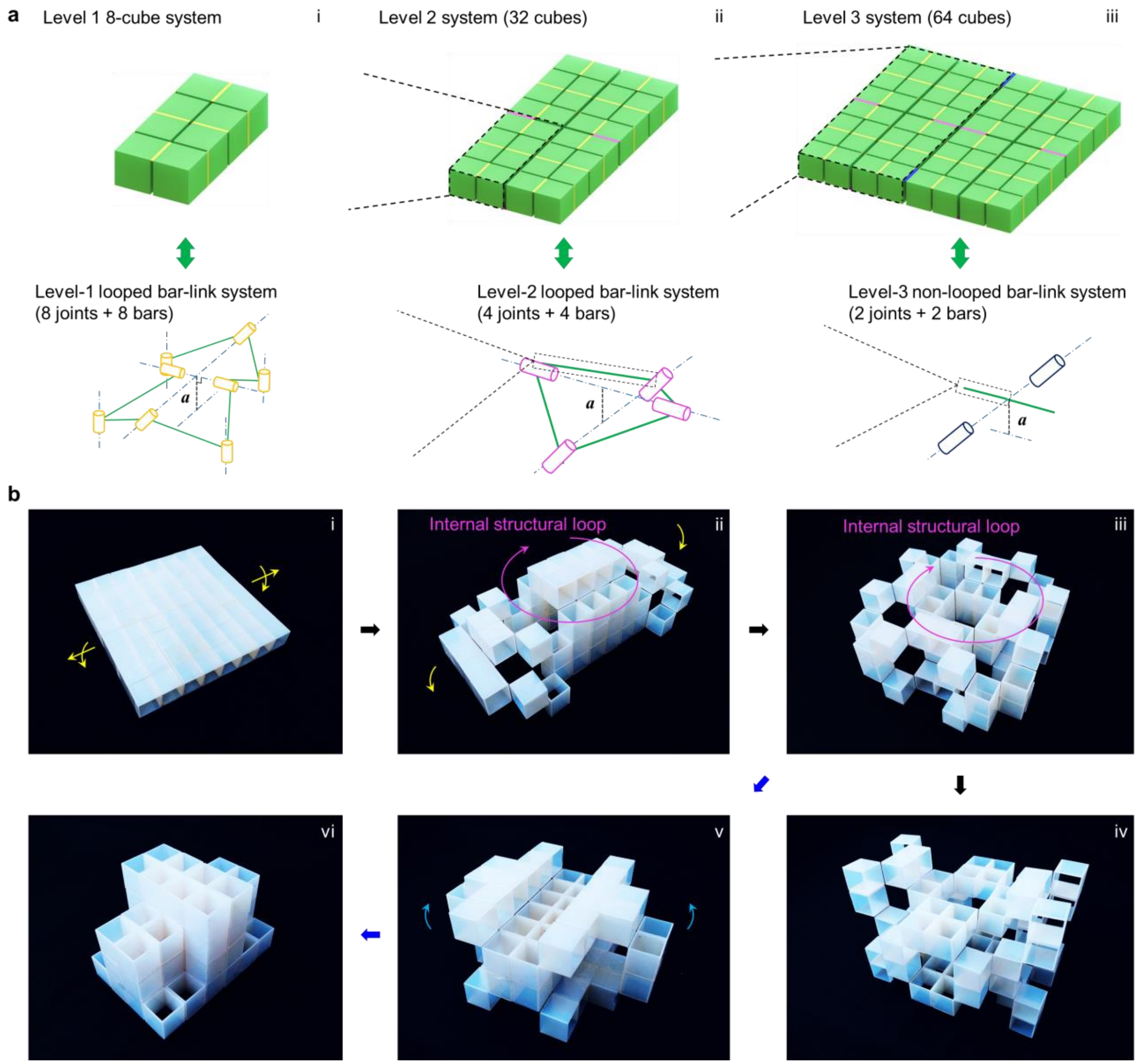

Supplementary Fig. 6. The representative <8R,4R,2R> type of level-3 structure. **a**, The used structural details of different-leveled structures: 8R level-1 structure (i); <8R,4R> type of level-2 structure (ii); the final <8R,4R,2R> level-3 structure. **b**, The continuous reconfiguration details of the present level-3 structure.

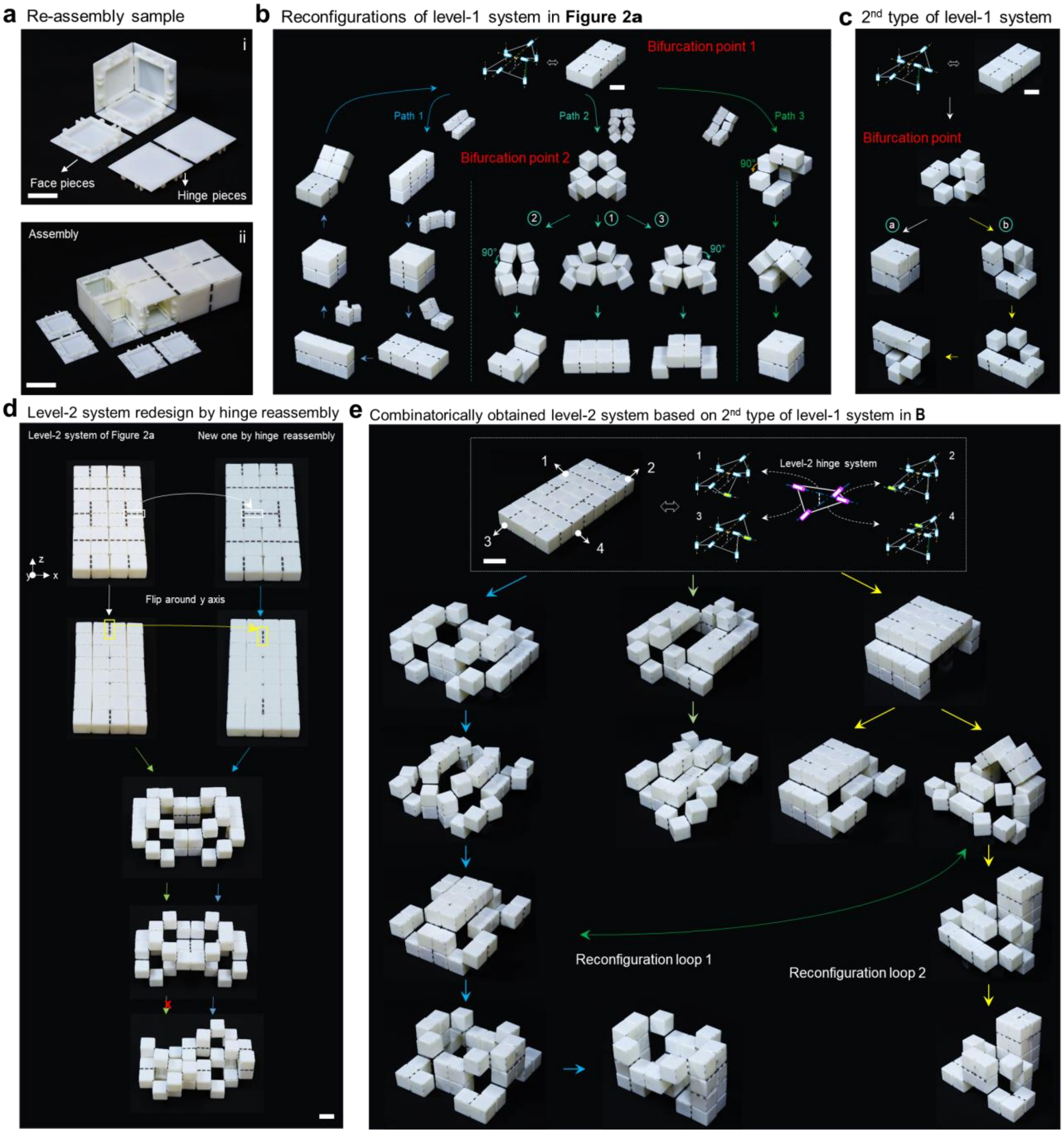

Supplementary Fig. 7. Reconfiguration details of the level-1 and <8R,4R> type of level-2 systems made of re-assemble structural parts. a, Design and assembly details of re-assemble level-1 prototype printed by multi-material 3D printer. **b** and **c**, Reconfiguration details of two different level-1 prototypes designed by changing folds positions. **d,** Reconfiguration details of the <8R,4R> level-2 prototype based on the level-1 system shown in **c**. **e**, Reconfiguration details of level 2 structure in Fig. 2f with some representative transition processes.

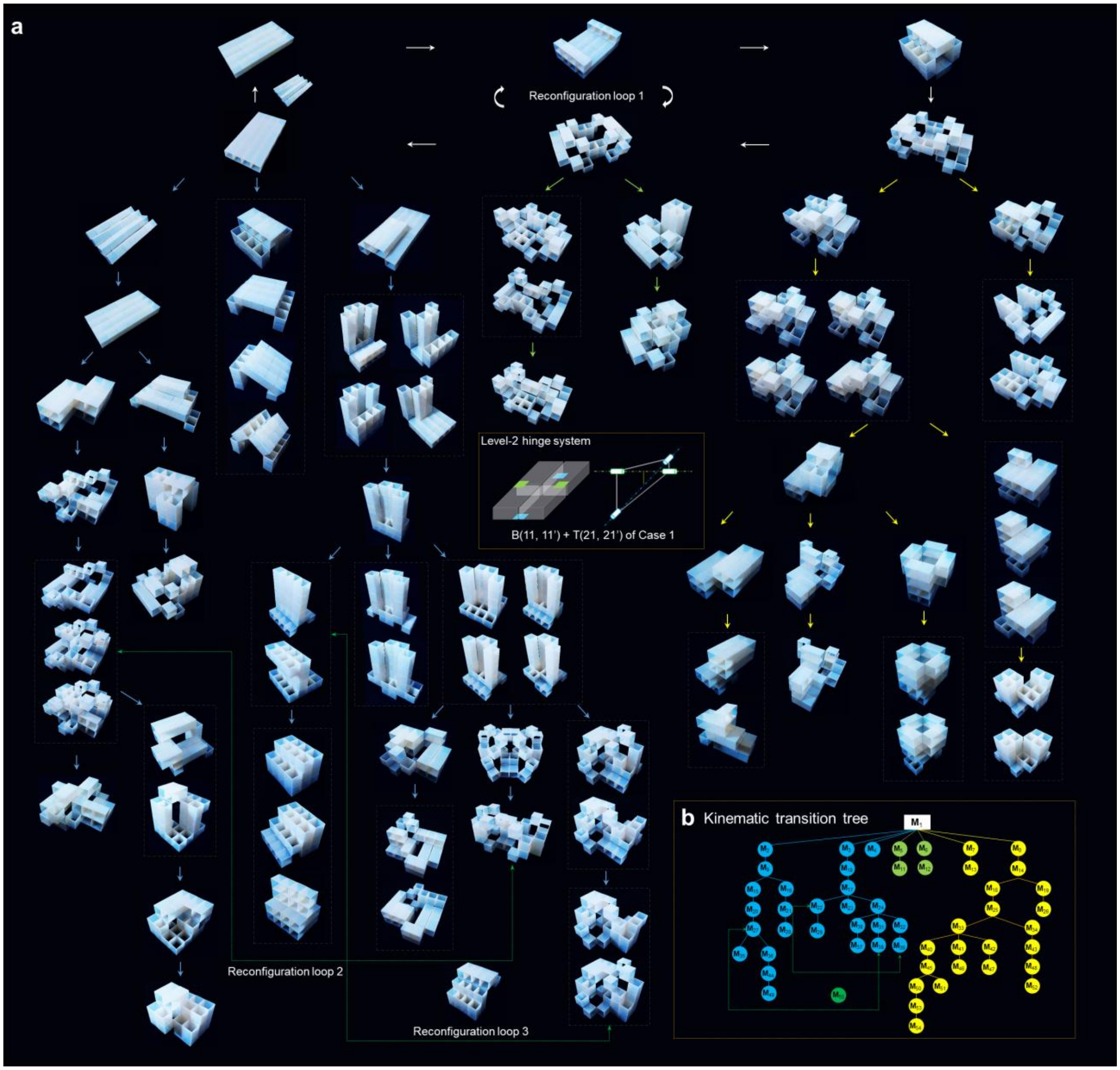

Supplementary Fig. 8. Reconfiguration details of the <8R,4R> type of level-2 structure used in Fig. 2a. a, Reconfiguration details presented with prototypes. **b,** The relative transition tree for labelling the configurations shown in **a**.

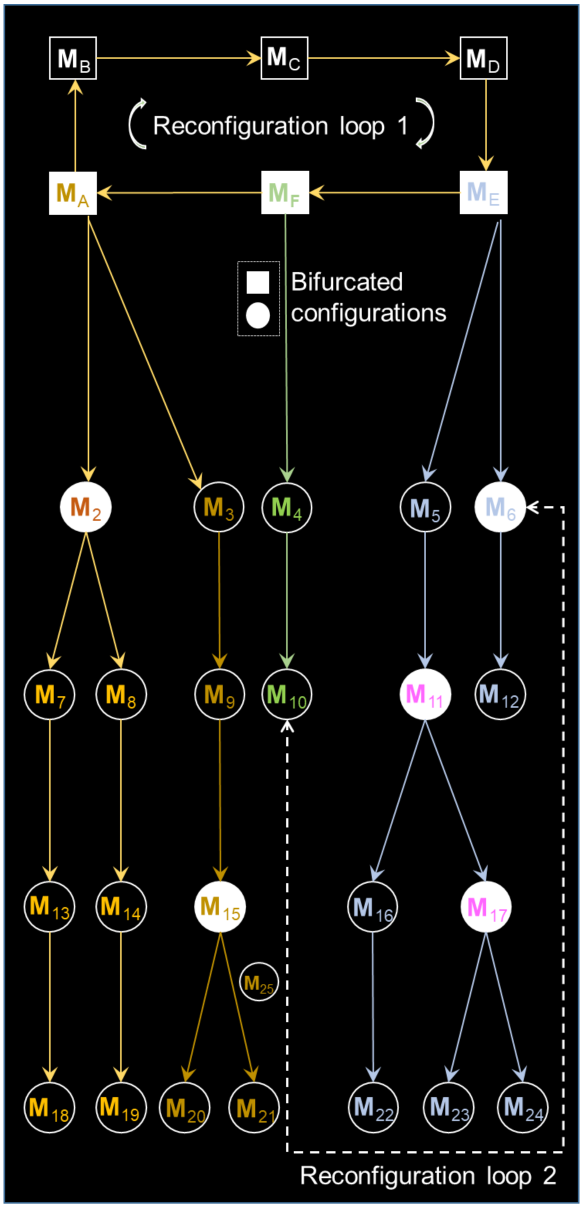

Supplementary Fig. 9. Reconfiguration transition tree corresponding to the shape morphing processes of level 2 structure shown in Fig. 3a.

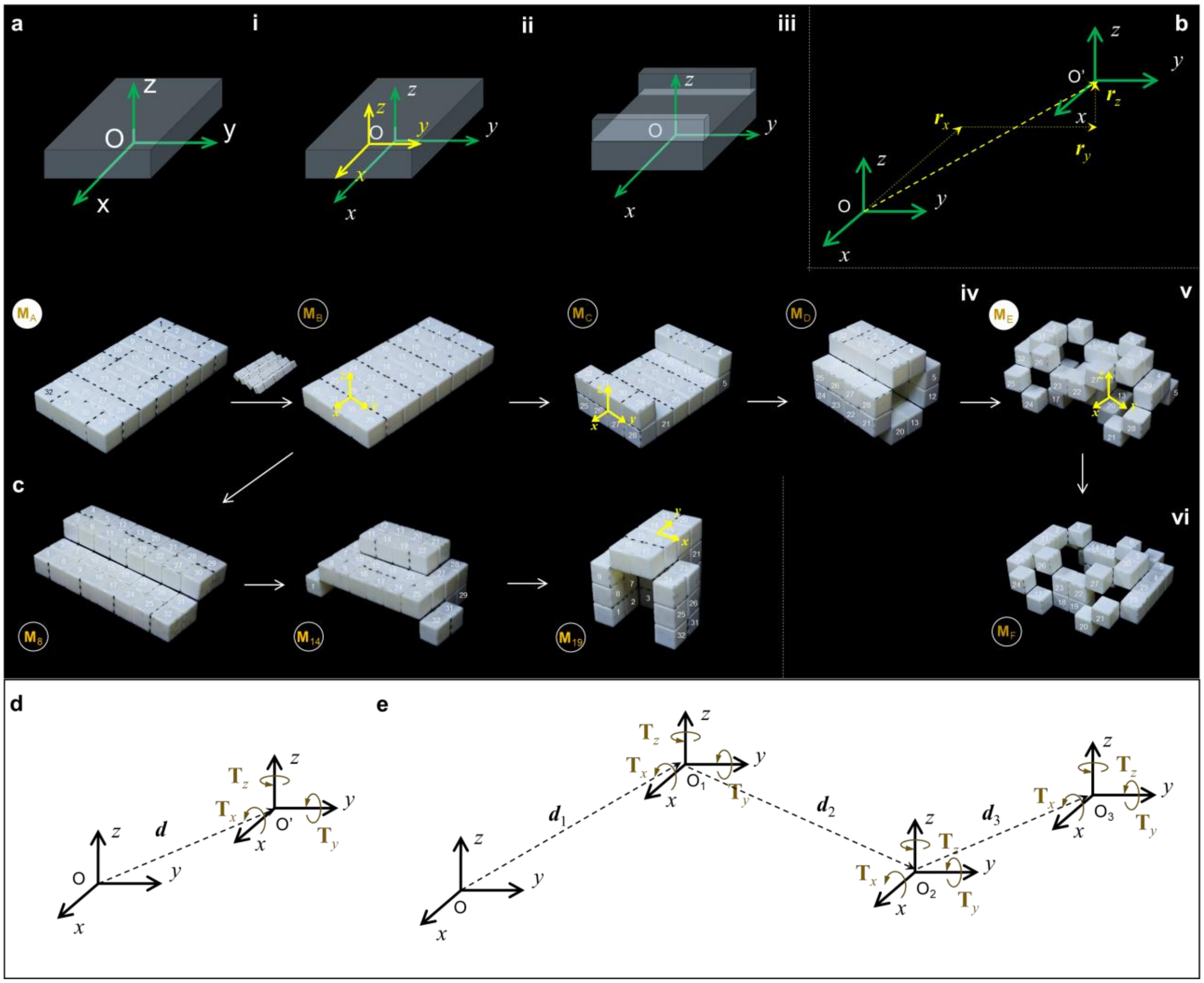

Supplementary Fig. 10. Theoretical analysis basis for the shape morphing of level 2 structures in Fig. 3a. a, Schematics of constructing local and global Cartesian coordinates**. b**, Schematics of the relation between local coordinate systems on structural elements and the global coordinate systems built on the center of certain configurations. **c**, Cube elements tracking with labelled Arabic number.

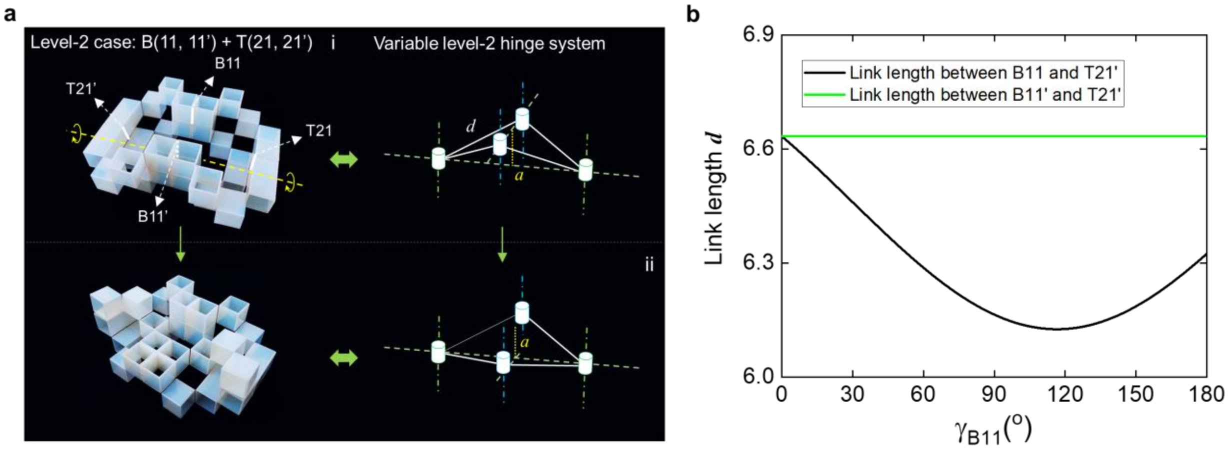

Supplementary Fig. 11. Demonstration of the flexible length of level-2 links. a, The selected reconfiguration process from configuration $\mathbf{M}_4$ to configuration $\mathbf{M}_{10}$ of **Fig. 3a**. **b**, The calculated results of reconfigured and non-reconfigured link lengths.

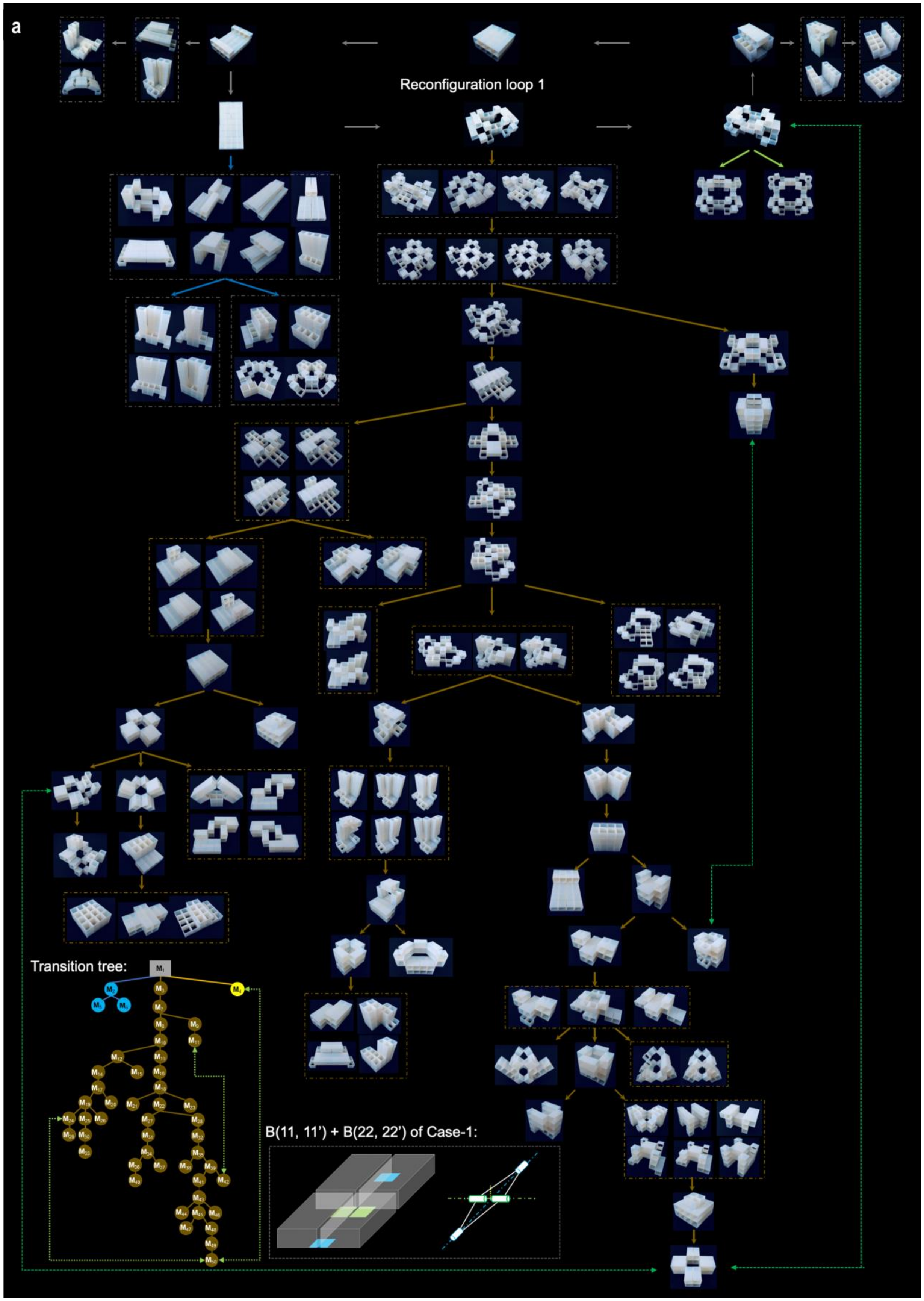
a
Reconfiguration loop 1
Transition tree:
B(11, 11’) + B(22, 22’) of Case-1:

Contd.

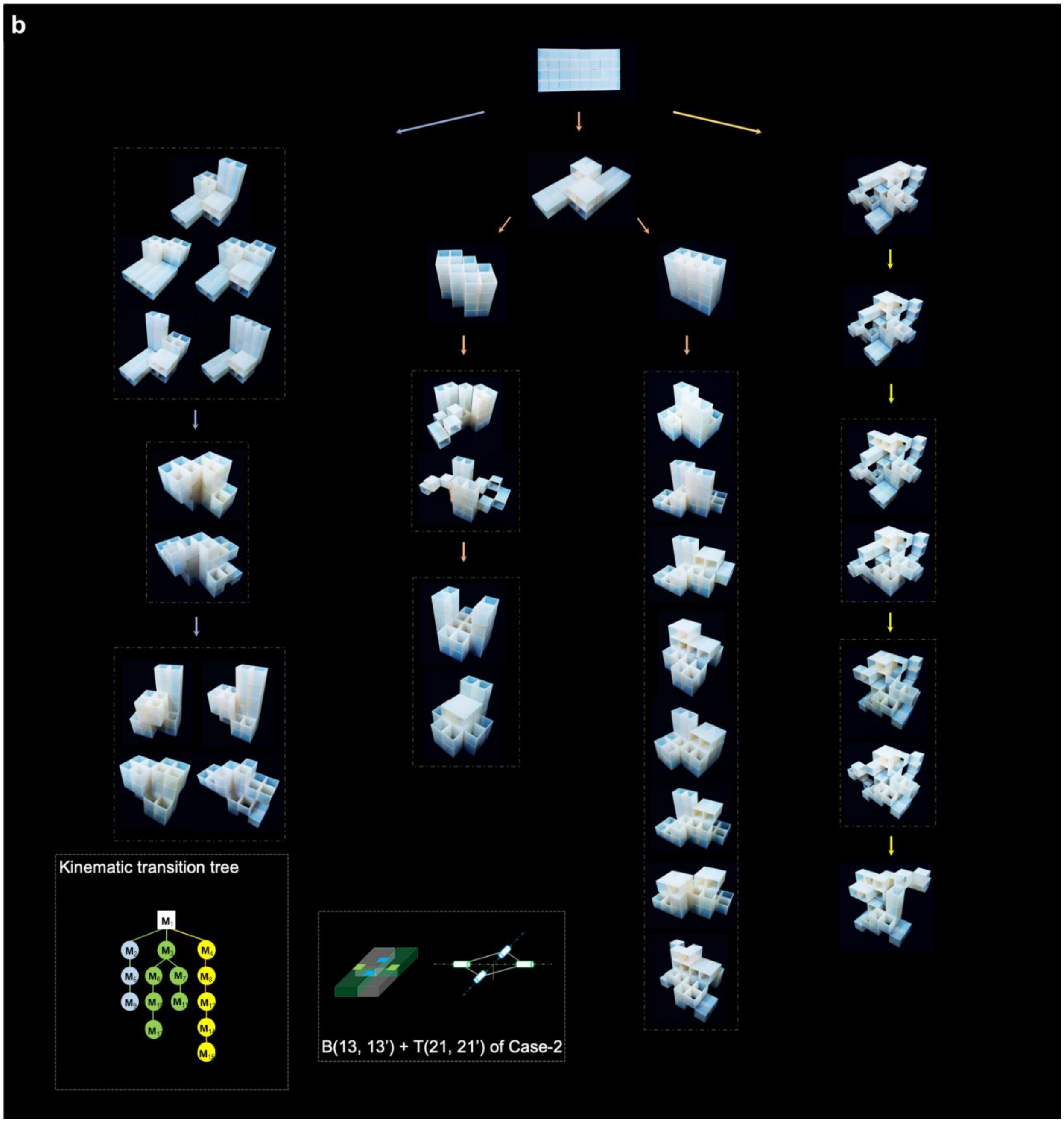

Contd.

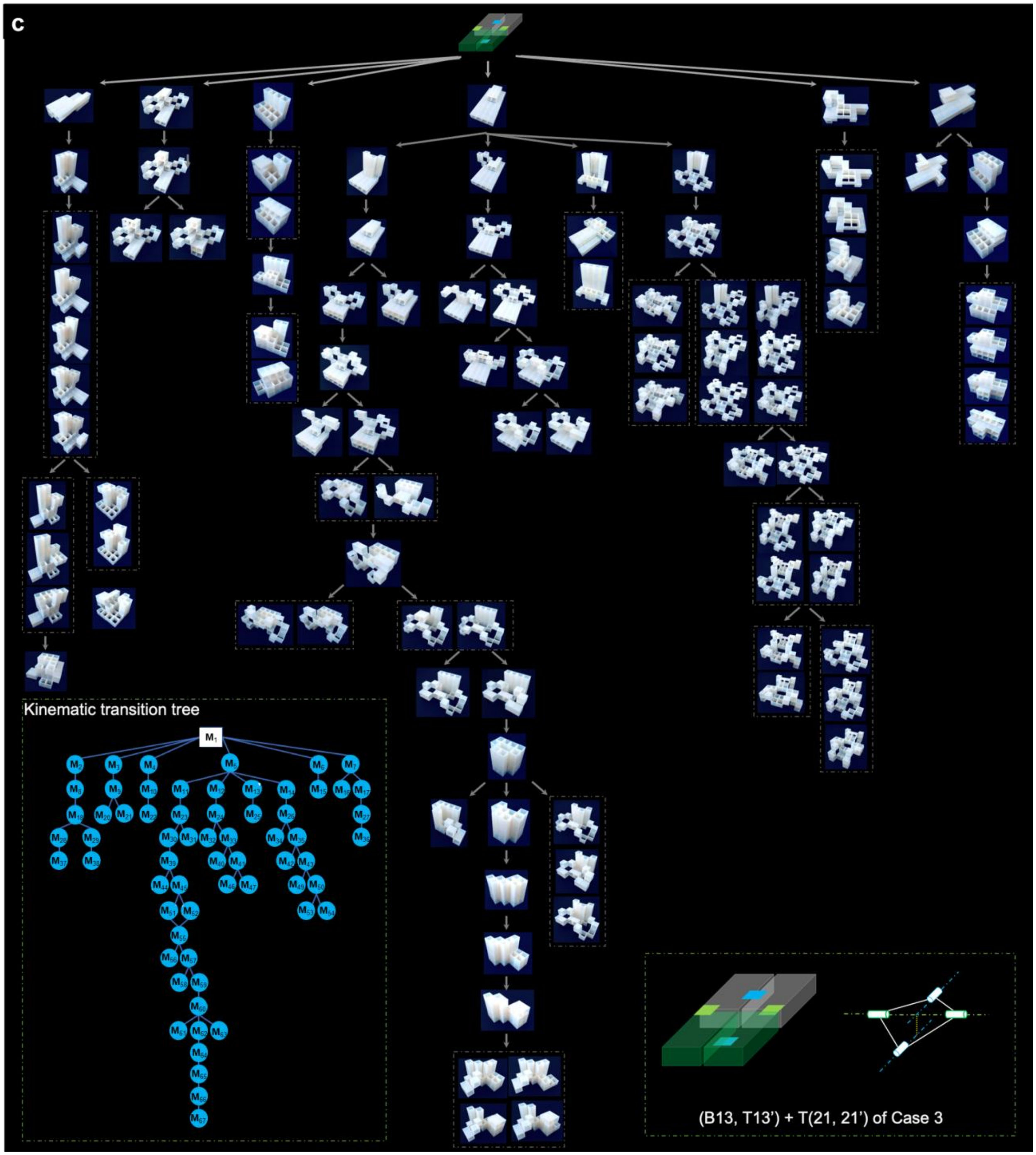

Contd.

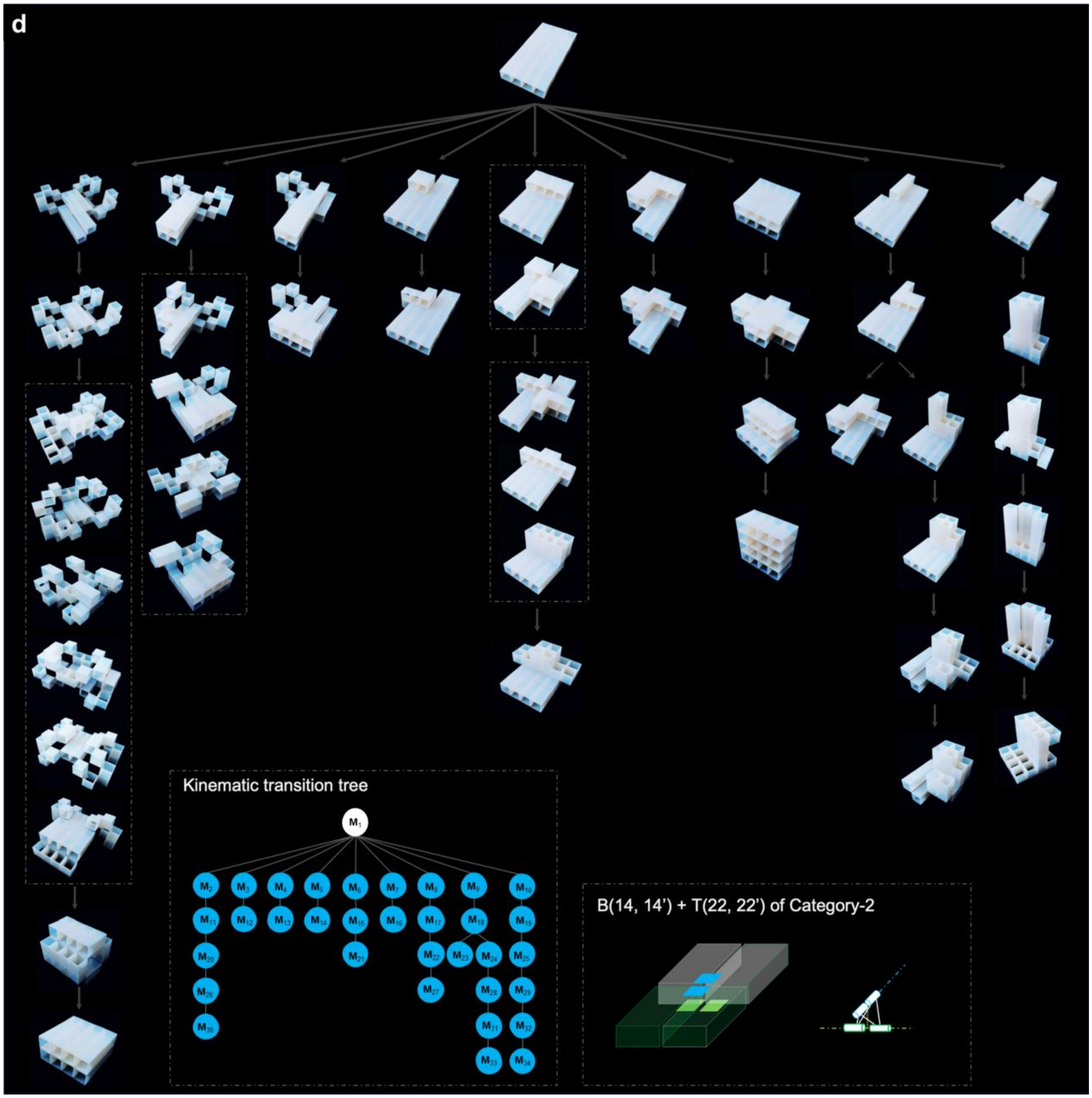

Supplementary Fig. 12. The reconfiguration details of four combinatorically designed level-2 structures based on Supplementary Fig. 6 and their transition trees.

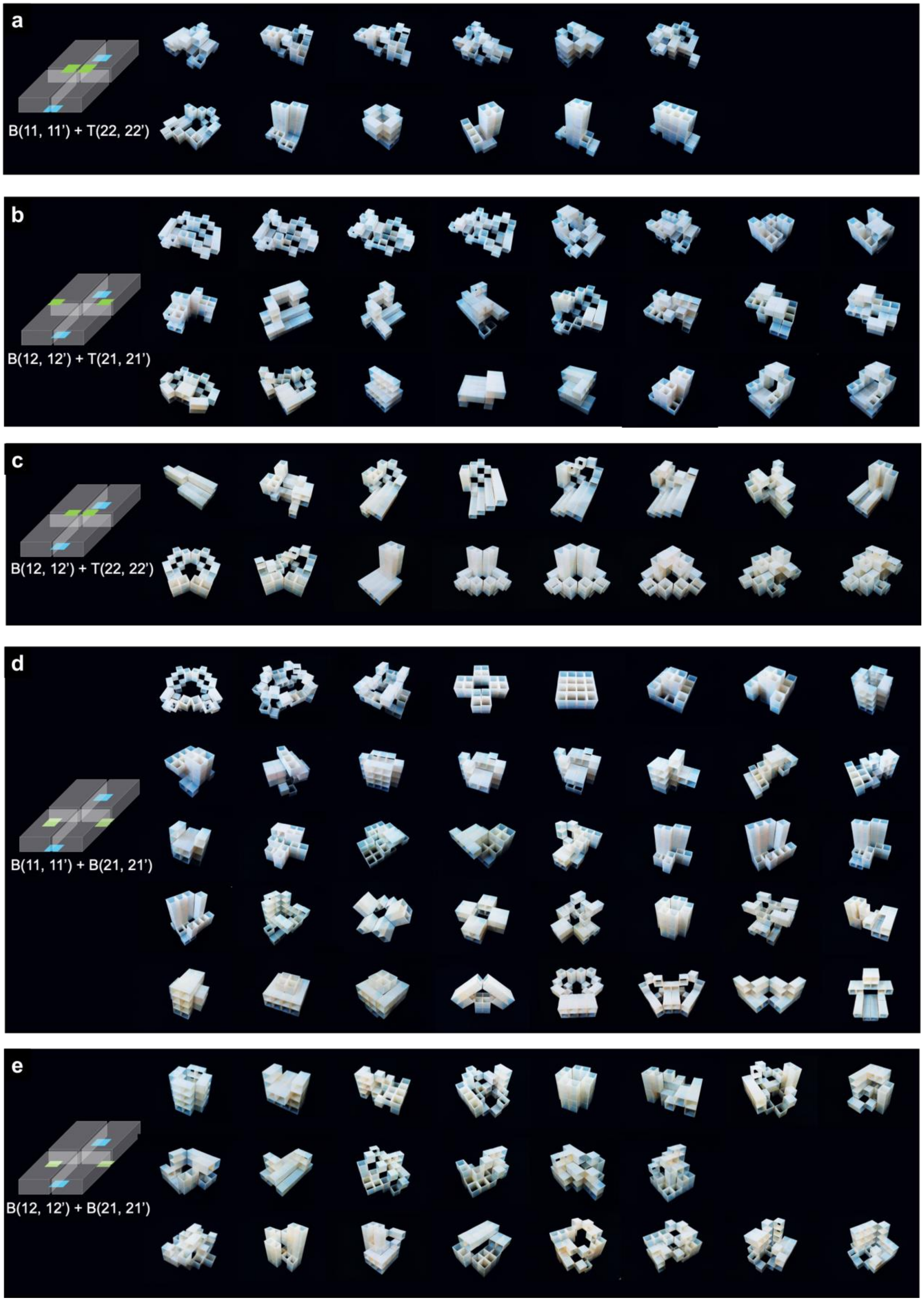
a
B(11, 11') + T(22, 22')
b
B(12, 12') + T(21, 21')
c
B(12, 12') + T(22, 22')
d
B(11, 11') + B(21, 21')
e
B(12, 12') + B(21, 21')

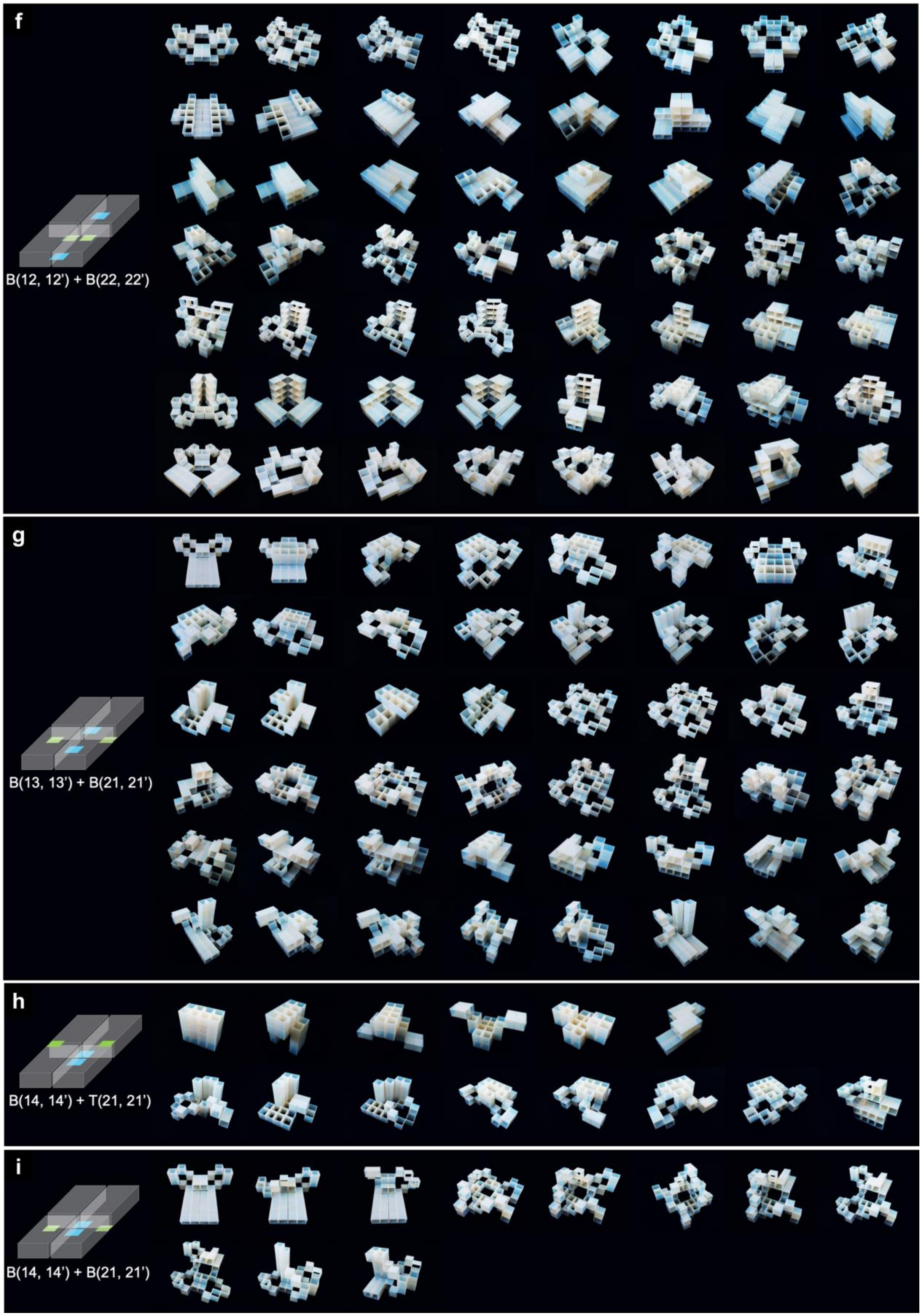
f
B(12, 12') + B(22, 22')
g
B(13, 13') + B(21, 21')
h
B(14, 14') + T(21, 21')
i
B(14, 14') + B(21, 21')

Contd.

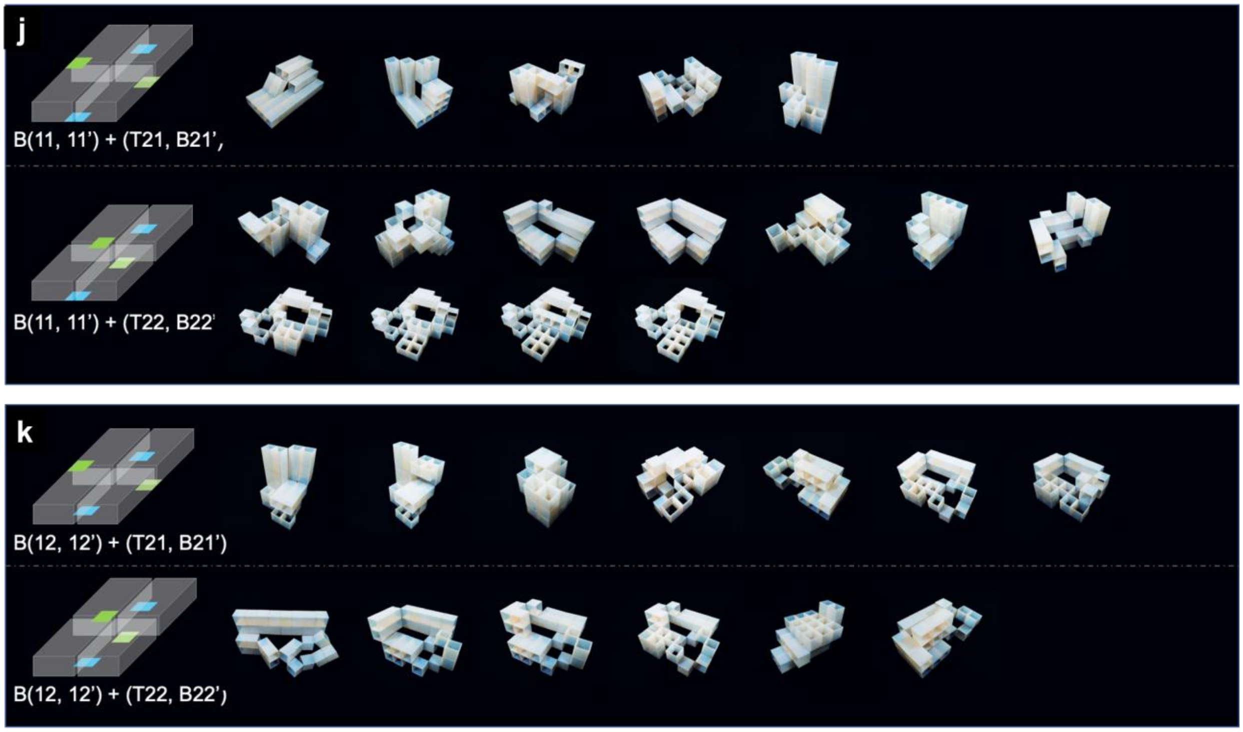

j
B(11, 11') + (T21, B21',
B(11, 11') + (T22, B22'
k
B(12, 12') + (T21, B21')
B(12, 12') + (T22, B22')

Case 2

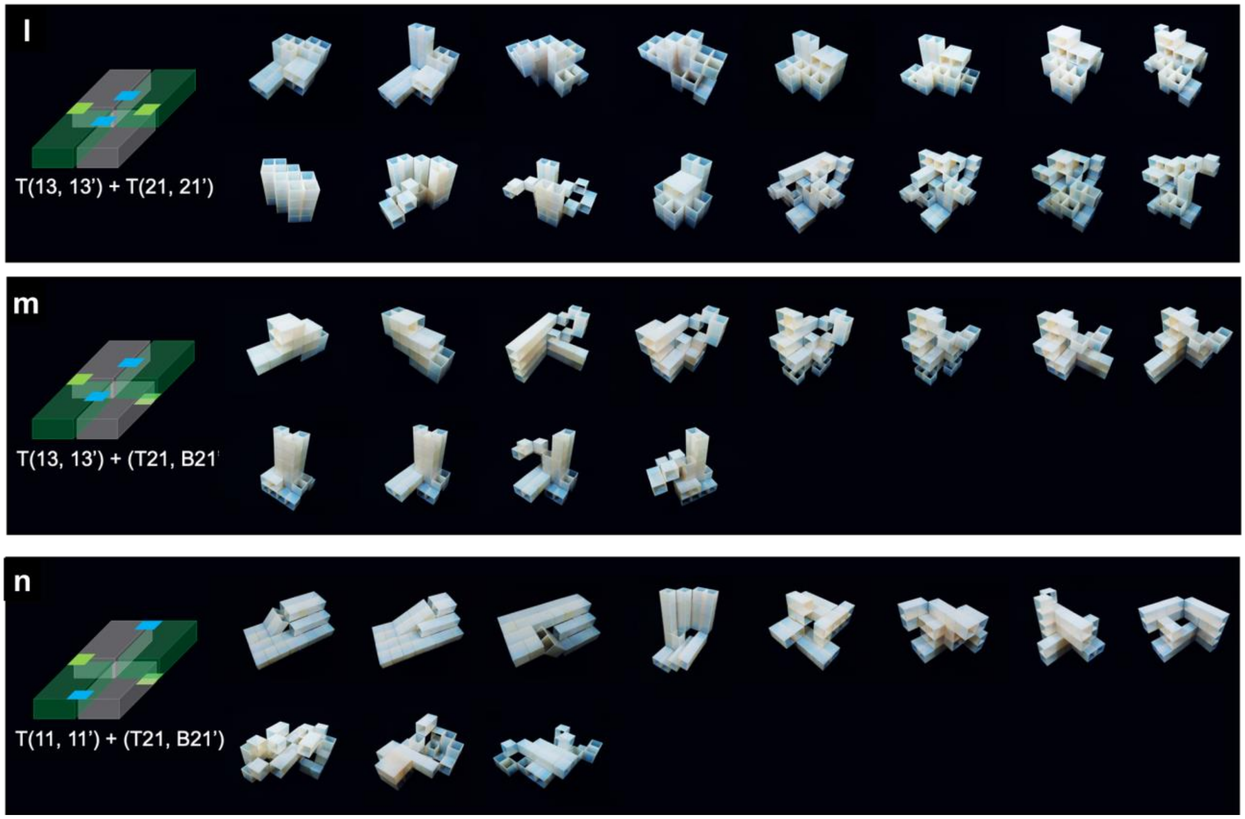

l
T(13, 13') + T(21, 21')
m
T(13, 13') + (T21, B21'
n
T(11, 11') + (T21, B21')

Contd.

Case 3

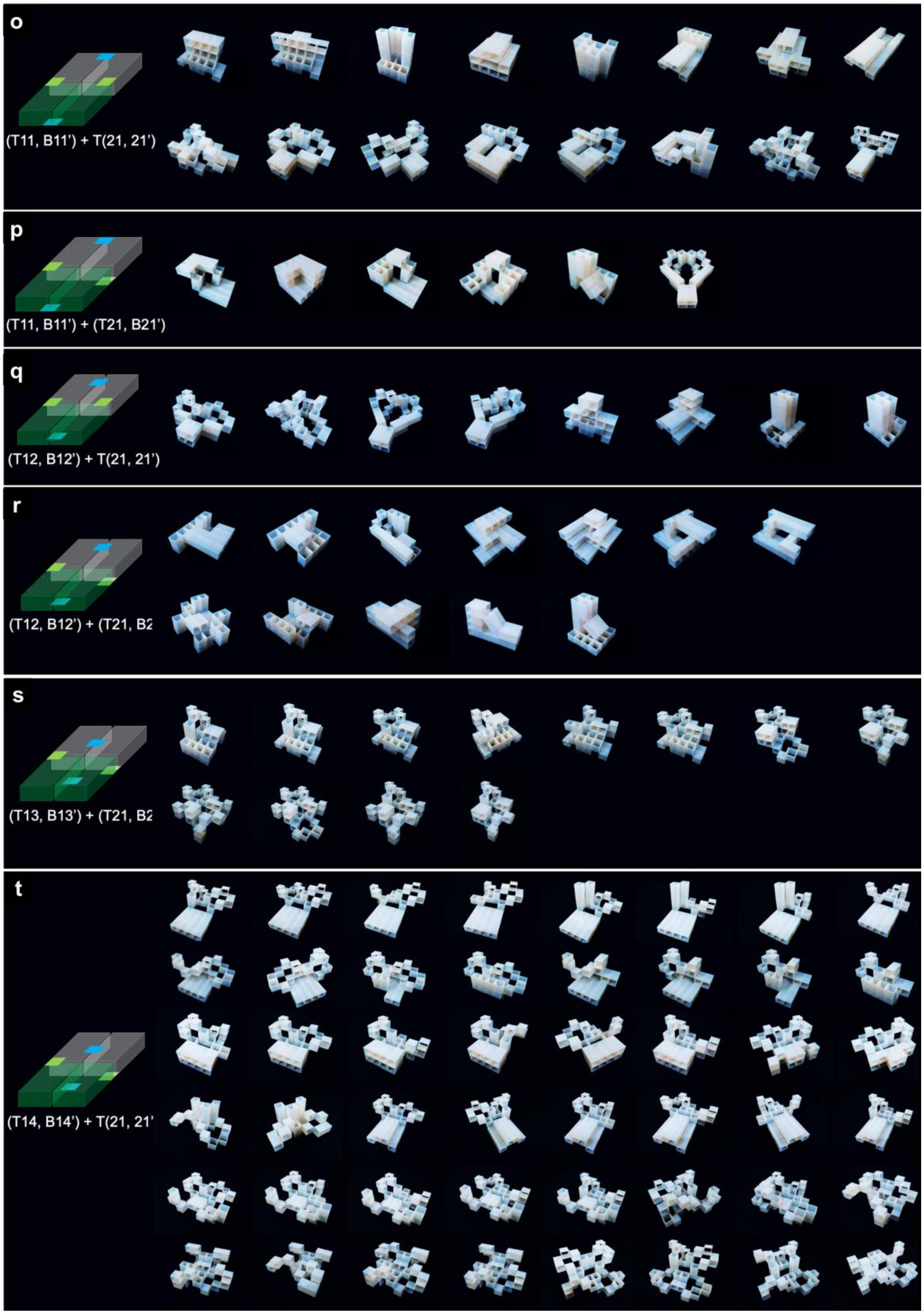

o
(T11, B11') + T(21, 21')
p
(T11, B11') + (T21, B21')
q
(T12, B12') + T(21, 21')
r
(T12, B12') + (T21, B2
s
(T13, B13') + (T21, B2
t
(T14, B14') + T(21, 21'

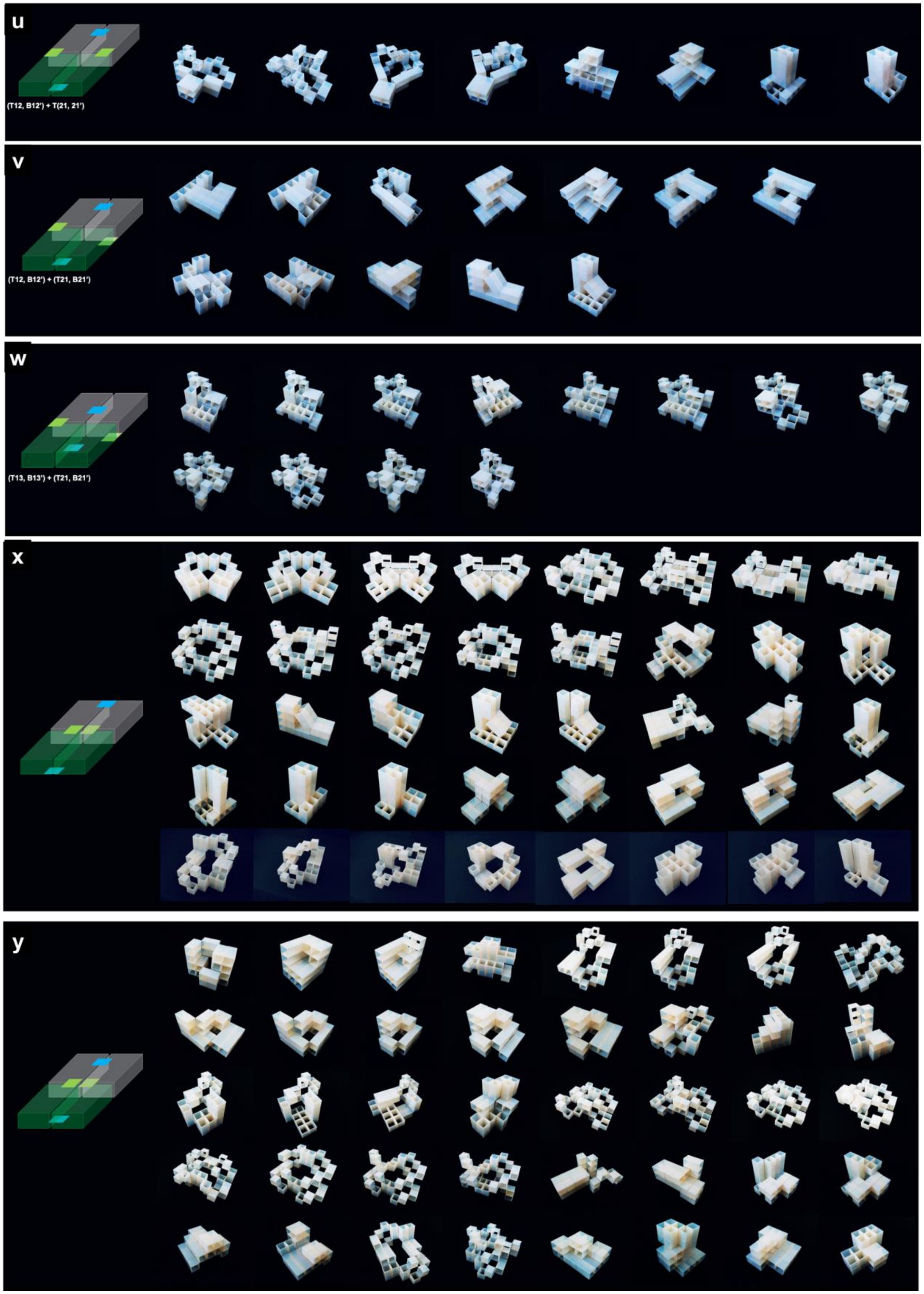
u
(T12, B12') + T(21, 21')
v
(T12, B12') + (T21, B21')
w
(T13, B13') + (T21, B21')
x
y

Contd.

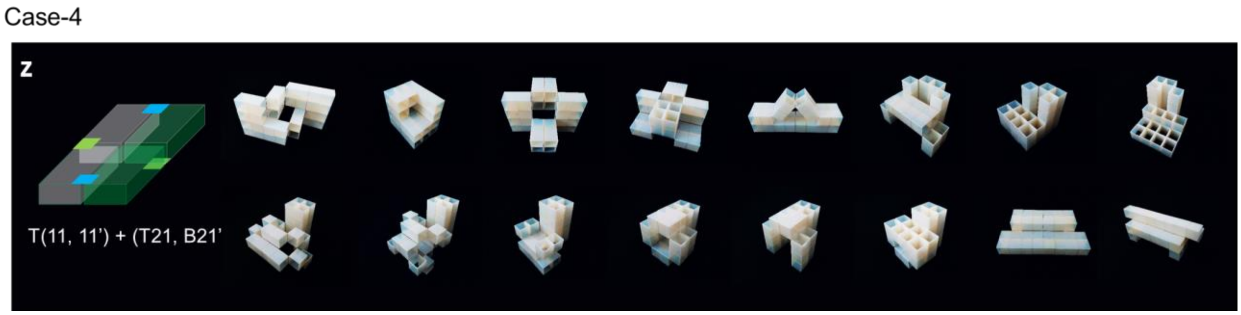

Supplementary Fig. 13. Reconfiguration details of the selected four different cases of the level-2 structures listed in Supplementary Fig. 6.

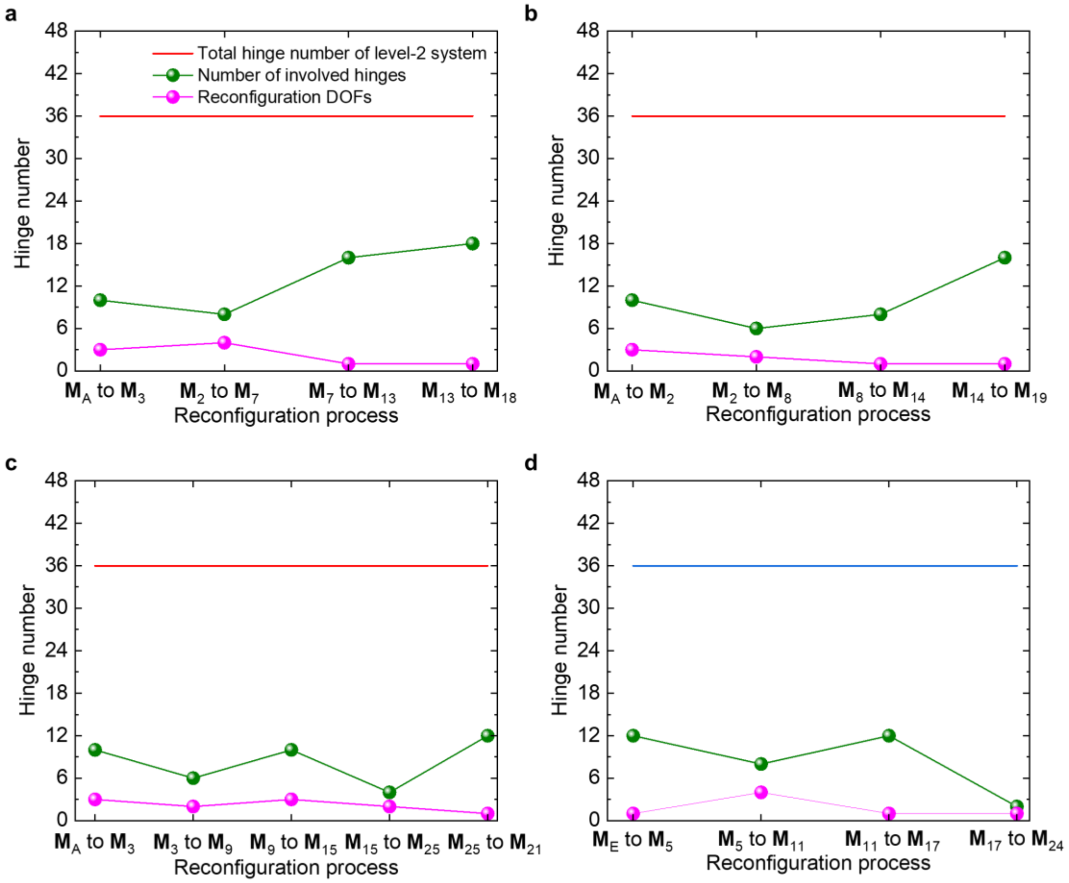

Supplementary Fig. 14. Comparison among the total number of level-2 folds, the rotated folds number during each reconfiguration process and the related reconfiguration DOFs for the reconfiguration processes listed in Fig. 3a.

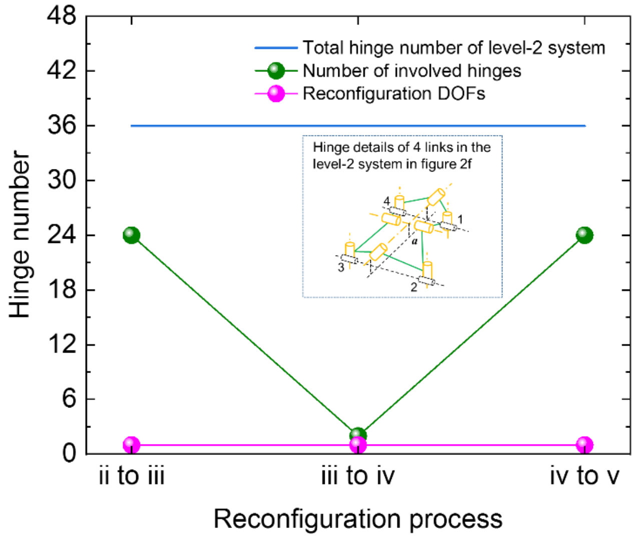

Supplementary Fig. 15. Comparison among the total number of level-2 folds, the rotated folds number during each reconfiguration process and the related reconfiguration DOFs for the reconfiguration processes listed in Fig. 3f.

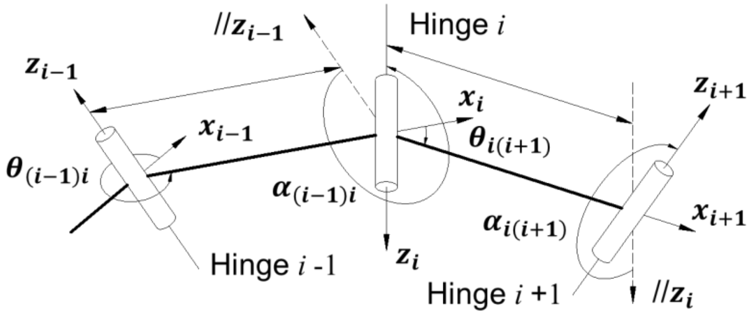

Supplementary Fig. 16. Schematics of consecutive local coordinate systems for the reconfiguration kinematic analysis of hierarchical origami-based metastructures.

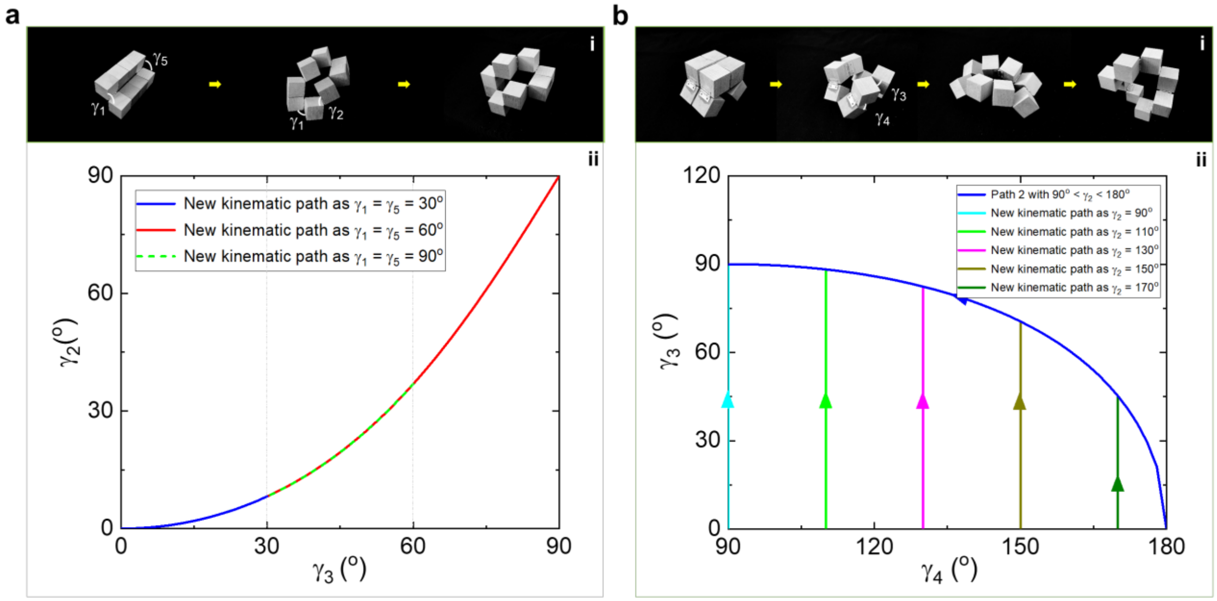

Supplementary Fig. 17. Reconfiguration kinematics of level-1 structure used in Fig. 3a. **a**, The reconfiguration kinematics of 6R-looped mechanism like path with $\gamma_{k1} = \gamma_{k2}$ selected randomly in the range of 0º to 90º. **b**, The sequential reconfiguration kinematics of 8R-looped mechanism like path.

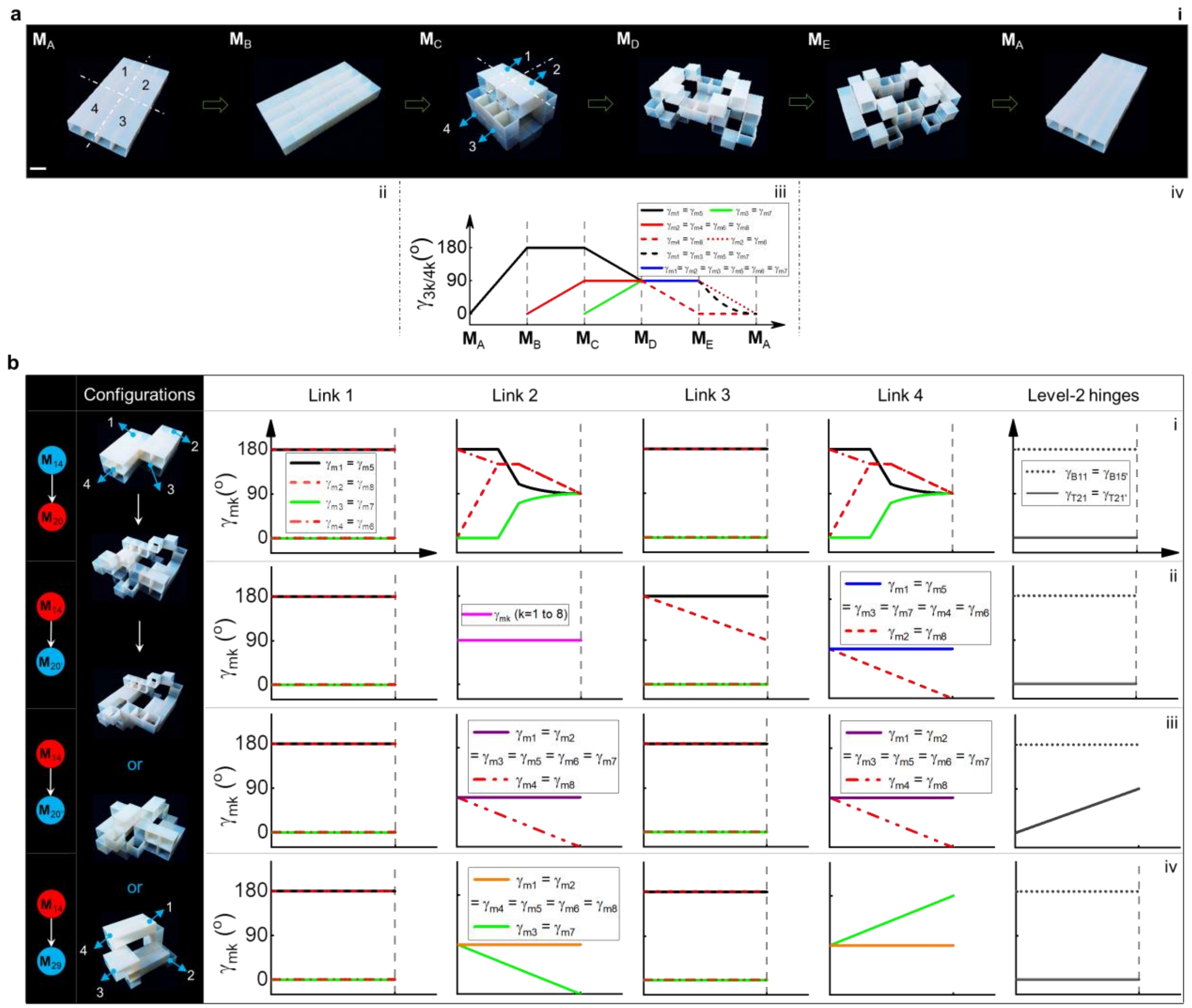

Supplementary Fig. 18. The reconfiguration kinematics of the representative reconfiguration processes of level-2 structure shown in Fig. 3a. **a**, the reconfiguration loop $\mathbf{M}_A$-$\mathbf{M}_B$-$\mathbf{M}_C$-$\mathbf{M}_D$-$\mathbf{M}_E$-$\mathbf{M}_F$-$\mathbf{M}_A$. **b**, the reconfiguration process from configuration $\mathbf{M}_{14}$ to configuration $\mathbf{M}_{20}$, $\mathbf{M}_{20'}$, $\mathbf{M}_{20''}$ and $\mathbf{M}_{29}$ in Supplementary Fig. 9.

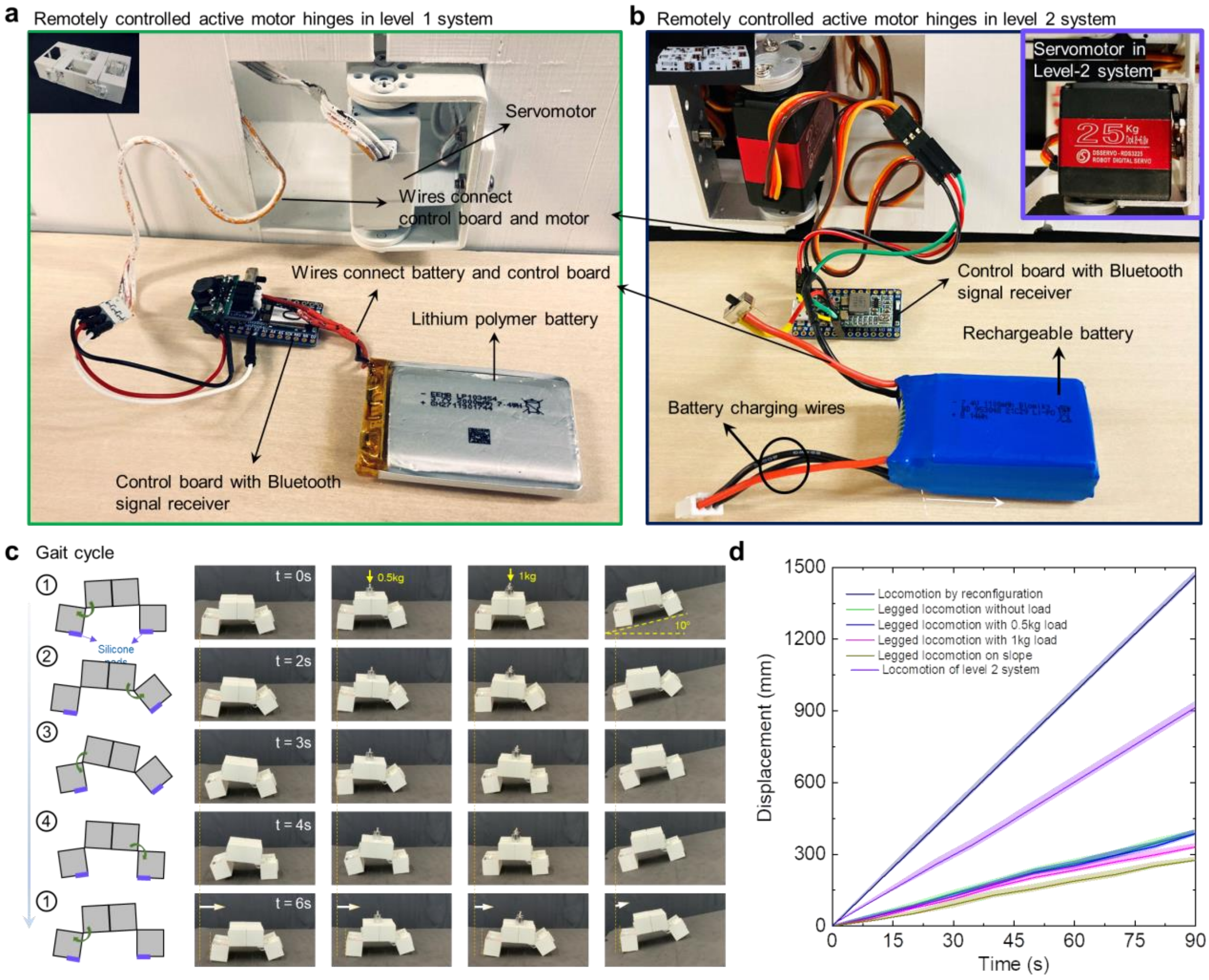

Supplementary Fig. 19. Design details of the remote untethered level-1, 2 systems. a and b, Electronic components to design remotely untethered level-1 and level-2 systems: Lithium polymer battery (3.7V and 7.4V), electrical servo-motor, control board with Bluetooth signal receiver and wires. **c**, Recycle gates of the legged locomotion of level-1 system. **d,** Locomotion speeds of level-1 and level-2 systems under different modes.

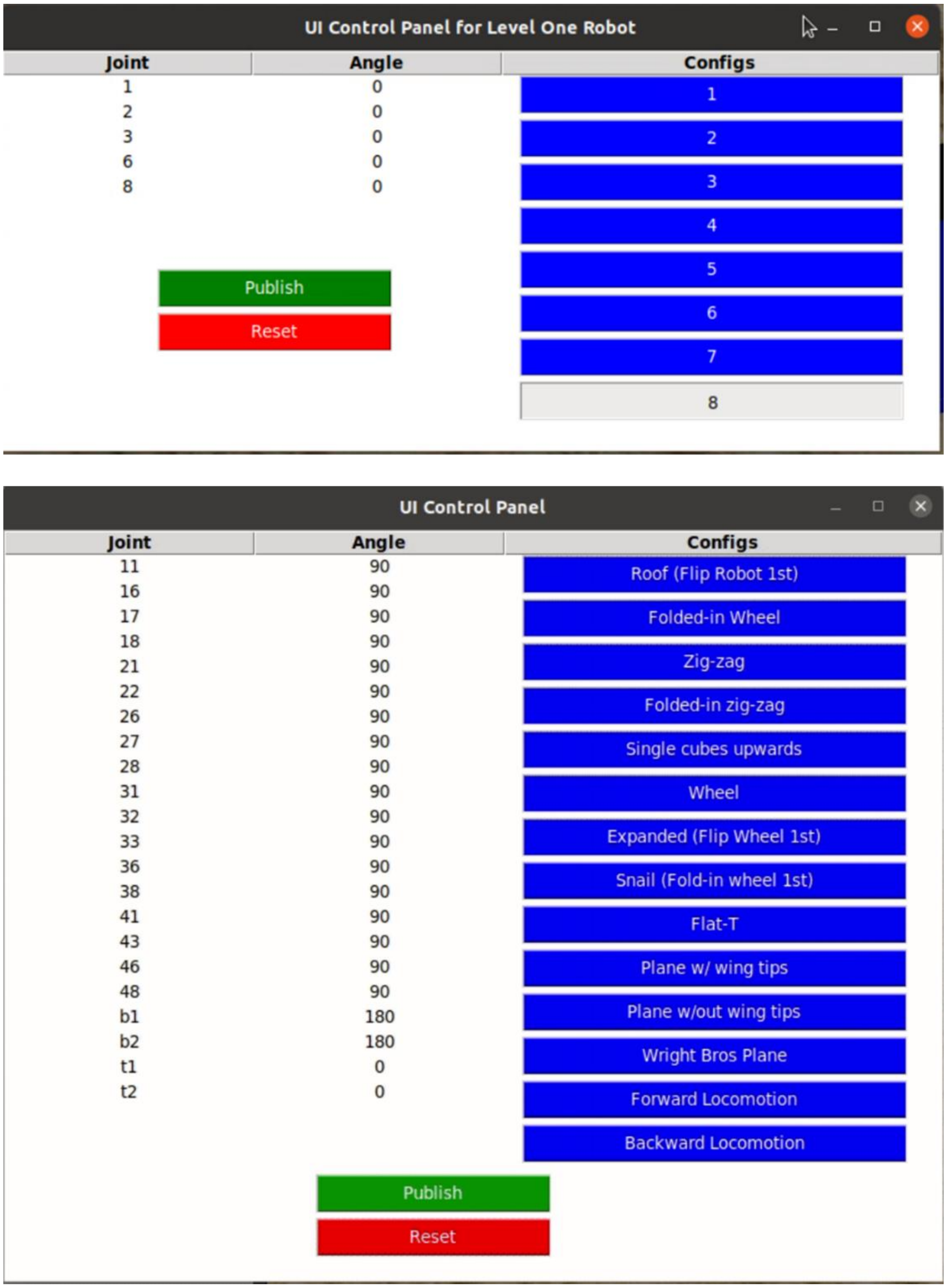

Supplementary Fig. 20. Self-developed control system: graphical interface has been developed in Mathworks to enable intuitive open-loop position control through either predefined sequences or input commands by the user

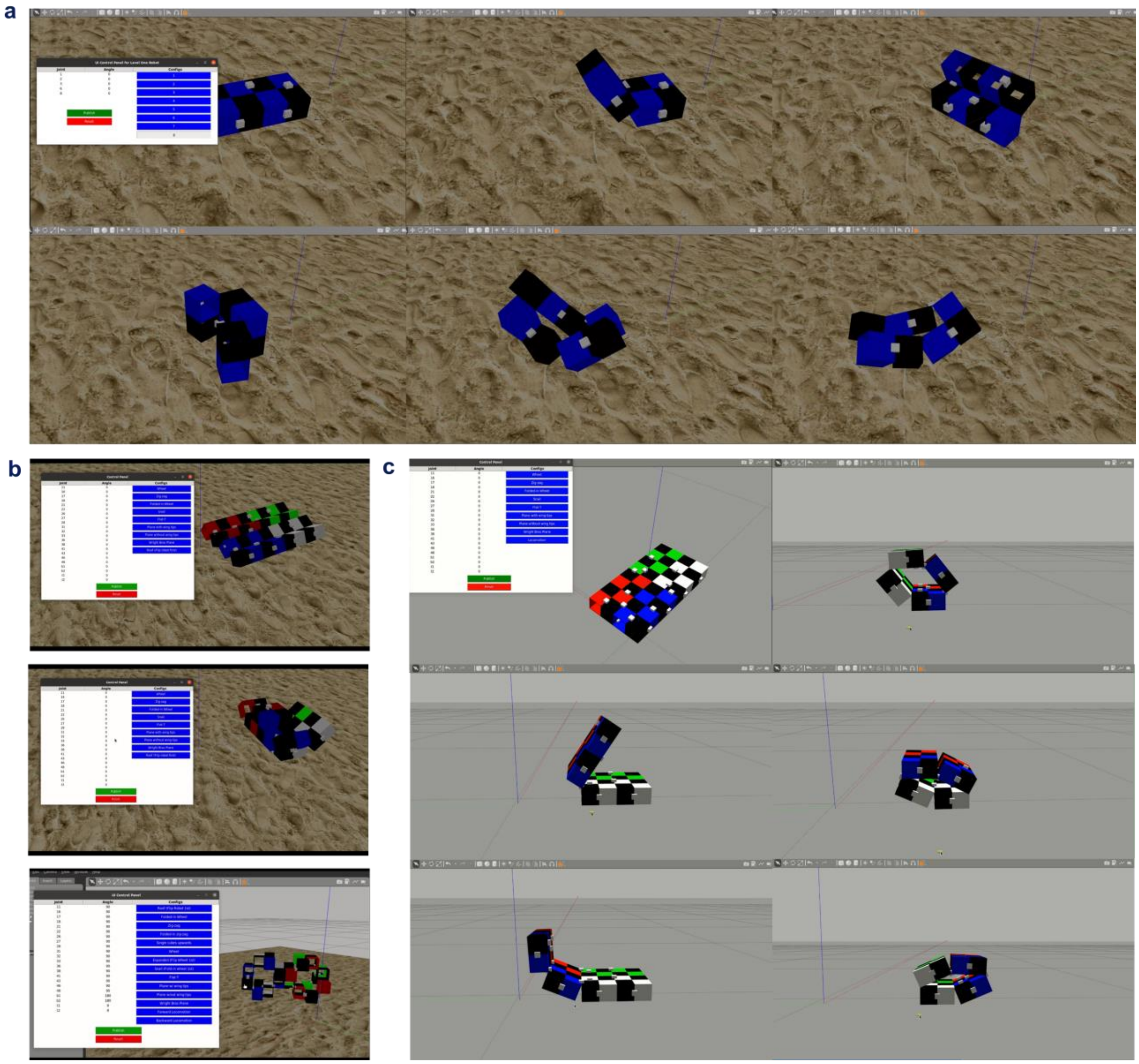

Supplementary Fig. 21. Simulation results on reconfiguring level-1 and level-2 structures used in Fig.2. a, Basic reconfigurations of level-1 system. **b**, Basic reconfigurations of level-2 system. **c**, Rolling based locomotion of level-2 system.

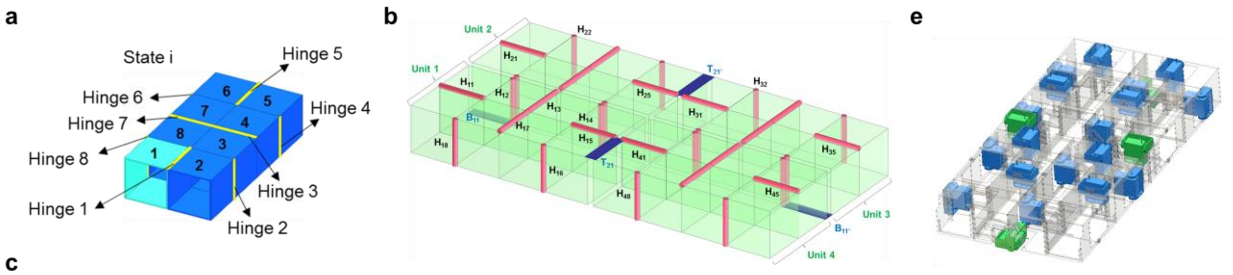

c

transformation proce		Hinge 1	Hinge 2	Hinge 3	Hinge 4	Hinge 5	Hinge 6	Hinge 7	Hinge 8	Notes	Active hinges (Bold number)
Transfor mation-1: 1R or 2R	i to ii			√				√		hinge 3 and 7 increase from 0 to 180	**3** or 7
	ii to iii		√		√		√		√	hinges 2, 4 and hinges 6, 8 increase from 0 to 180	(2, 6) or **(2, 8)** or (4, 6) or (4, 8)
	iii to iv			√				√		hinge 3 and 7 decrease from 180 to 0	3 or 7
	iv to v	√				√				hinge 1 and 5 increase from 0 to 180	**1** or 5
	v to vi		√		√		√		√	hinges 2, 4 and hinges 6, 8 decrease from 180 to 0	(2, 6) or **(2, 8)** or (4, 6) or (4, 8)
	vi to i	√				√				hinge 1 and 5 decrease from 180 to 0	**1** or 5
Transformation-2: 6R loop		1=3=5=7	2=6	1=3=5=7	≡ 0	1=3=5=7	2=6	1=3=5=7	≡ 0	$\gamma_1 \equiv \gamma_5 = \gamma_3 \equiv \gamma_7, \gamma_4 = \gamma_8 = 0,$ $\gamma_2 \equiv \gamma_6 = arcsin[sin^2\gamma_1/(cos^2\gamma_1 + 1)]$	**1 and 2**, or 2 and 3
Transformation-3: 8R loop		1=3=5=7	2=4=6=8	1=3=5=7	2=4=6=8	1=3=5=7	2=4=6=8	1=3=5=7	2=4=6=8	$\gamma_1 \equiv \gamma_5 = \gamma_3 \equiv \gamma_7,$ $\gamma_4 \equiv \gamma_8 = \gamma_2 \equiv \gamma_6 = arcsin[(1 - cos\gamma_1)/(cos\gamma_1 + 1)],$	1, 2 and 4; or **1, 2 and 8**
**Must-be active hinges** for level-1 structure: hinge 1 can be equivalently replaced by hinge 5; 2 equivalently by 6; 3 equivalently by 7; 4 equivalently by 8; Overall, 4 hinges need to be the must-be active hinges to achieve all three transformation process 1 to 3											**1, 2, 3, 8; or 5, 2, 3, 8; or 1, 4, 3, 8; or 5, 4, 3, 8;;** and other combinations

d

Transformation process	T21	T21'	B11	B11'	H11	H12	H13	H14	H15	H16	H17	H18	H21	H22	H23	H24	H25	H26	H27	H28	H31	H32	H33	H34	H35	H36	H37	H38	H41	H42	H43	H44	H45	H46	H47	H48	Active hinges
0 to 1			√	√																																	B11 or B11'
1 to 2							√				√				√				√																		H13/H17/H23/H27
2 to 3										√		√		√		√																					H16 or H18 and H22 or H24
1 to 4	√	√					√				√				√				√				√				√				√				√		T21 or T21' and H13/H17/H23/H27 and H33/H37/H43/h47
0 to 5			√	√	√				√				√				√				√				√				√				√				B11/B11' and H11/H15/H41/H45 and H21/H25/H31/H35
5 to 6			√	√	√				√				√				√				√				√				√				√				B11/B11' and H11/H15/H41/H45 and H21/H25/H31/H35
6 to 7						√		√		√		√		√		√		√		√		√		√		√		√		√		√		√		√	H12/H18/H22/H28 and H14/H16/H24 /H26 and H32/H38/H42/H48 and H34/H36/H44/H46
7 to 8					√		√		√		√		√		√		√		√		√		√		√		√		√		√		√		√		H11/H13/H15/H17/H41/H43/H45/H47 and H21/H23/H25/H27/H31/H33/H35 /H37
8 to 9					√		√		√		√																		√		√		√		√		H11/H13/H15/H17/H41/H43/H45/H47
9 to 10						√		√		√		√																		√		√		√		√	H12/H14 and H16/H18 and H42/H44 and H46/H48
10 to 11													√		√		√		√		√		√		√		√										H21/H23/H25/H27/H31/H33/H35/H37
11 to 12														√		√		√		√		√		√		√		√									H22/H24/H26/H28/H32/H34/H36/H38
11 to 13			√	√																																	B11/B11'
8 to 14					√		√		√		√																		√		√		√		√		H11/H13/H15/H17/H41/H43/H45/H47
14 to 15						√				√						√				√		√				√						√				√	H12/H18/H24/H26 and H34/H36/H42/H48
15 to 16						√				√						√				√		√				√						√				√	H12/H18/H24/H26 and H34/H36/H42/H48
8 to 17						√				√						√				√		√				√						√				√	H12/H18/H24/H26 and H34/H36/H42/H48
17 to 18						√				√						√				√																	H12/H18/H24/H26
18 to 19						√				√						√				√		√				√						√				√	H12/H18/H24/H26 and H34/H36/H42/H48
Therefore, for level-2 structure, the must-be active hinges include: **B11/B11' and T21/T21' ; H11, H12, H13, H14, H16; H21, H22, H23, H24; H31, H32, H33, H34; H41, H42, H43, H44, H46.** Totally level-2 structure needs **20 active hinges**																																					

Supplementary Fig. 22. Electrical servomotor arrangement process for both level-1 and level-2 structures used in Fig. 5 and Fig. 6. a, Folds sequence labelled for level-1 structure. **b**, Folds sequence labelled for level-2 structures. **c**, Demonstration of four active servomotors for level-1 structure to achieve all its reconfiguration paths shown in Supplementary Fig. 7b. **d**, Demonstration of 20 active servomotors for level-2 structure. **e**, Schematic illustration of level-2 structure with 20 active servomotors to achieve all the reconfigured shapes shown in Fig. 3a.

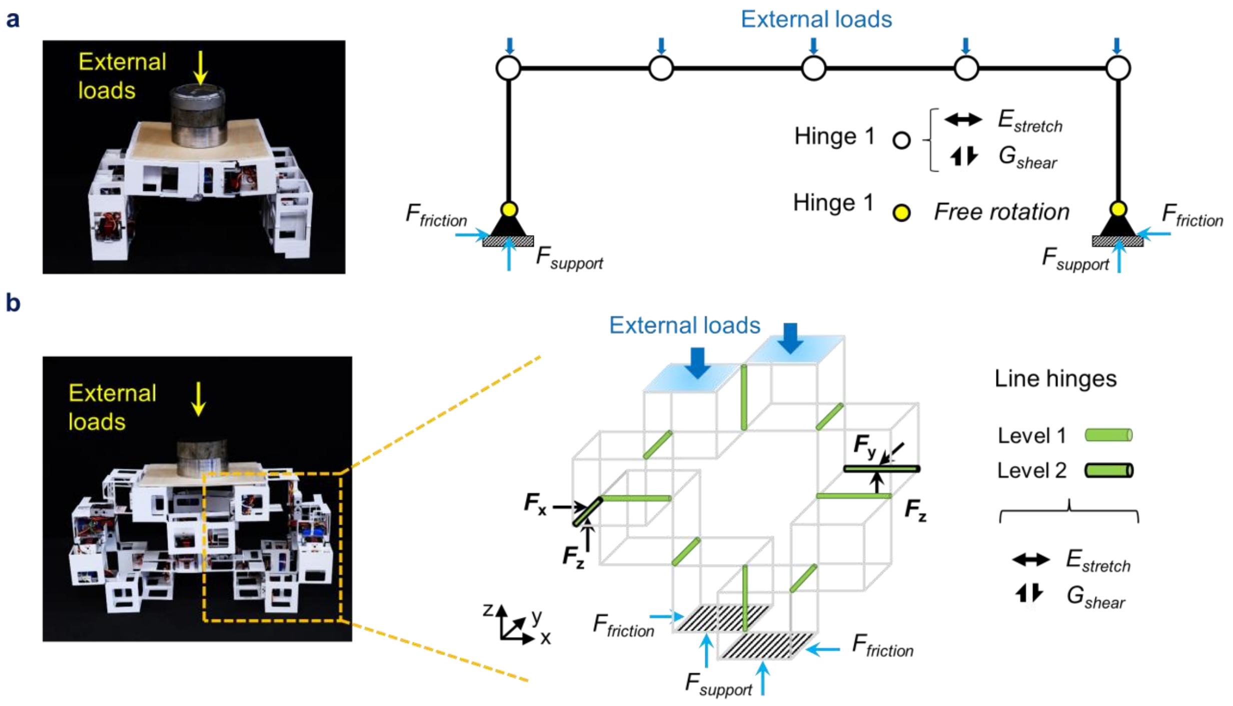

Supplementary Fig. 23. Demonstration of the loads bearing capabilities of reconfigured bridge (a) and complex architectural shape (b) by their free body diagrams.

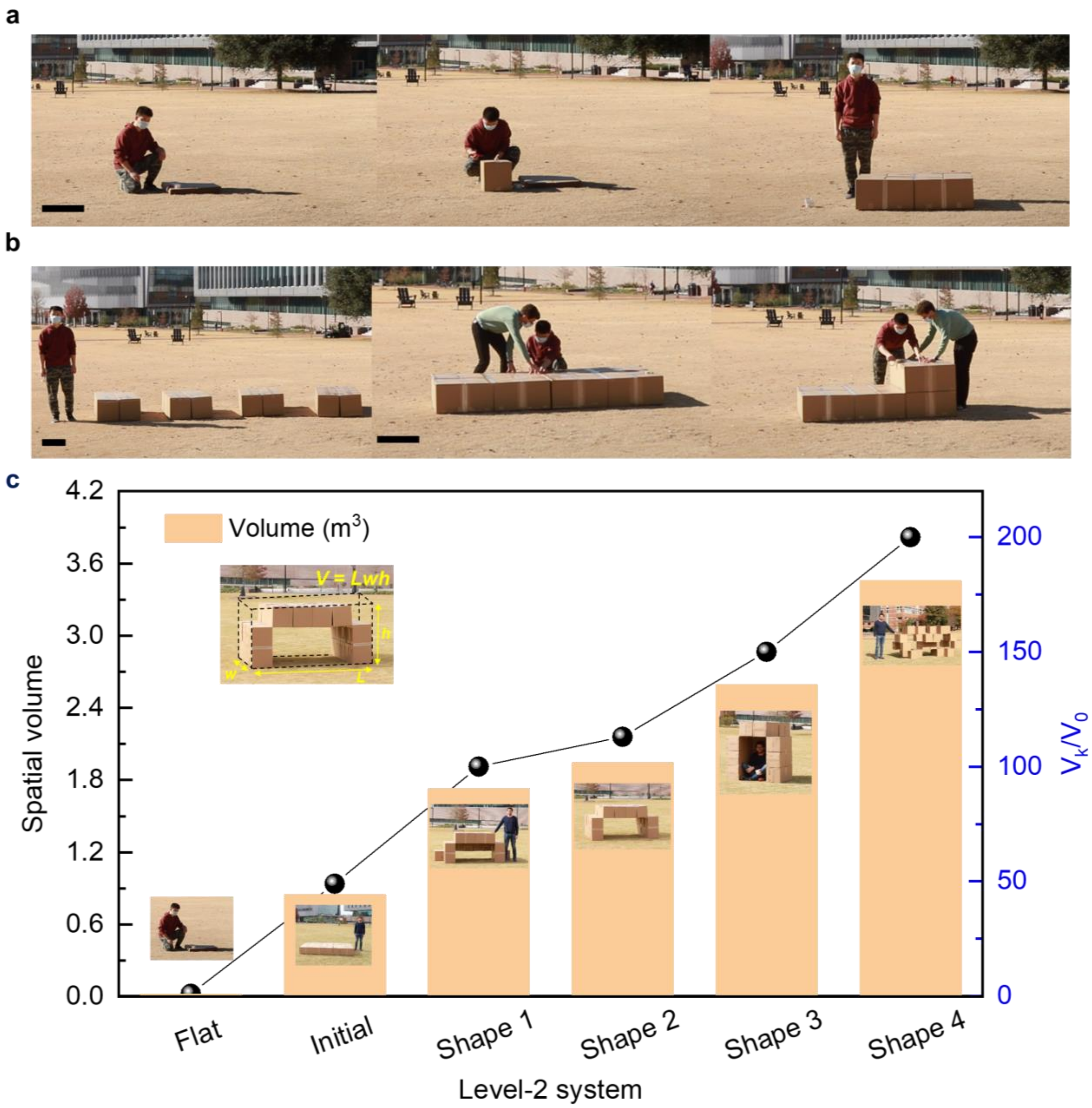

Supplementary Fig. 24. Demonstration of meter-scale samples. a, Fabrication process from flat packaging box (50cm × 50cm × 50cm) of level-1 structure. **b**, Fabrication process of level-2 structure from level-1 structures. **c**, Volume variation of level-2 reconfigured configurations.

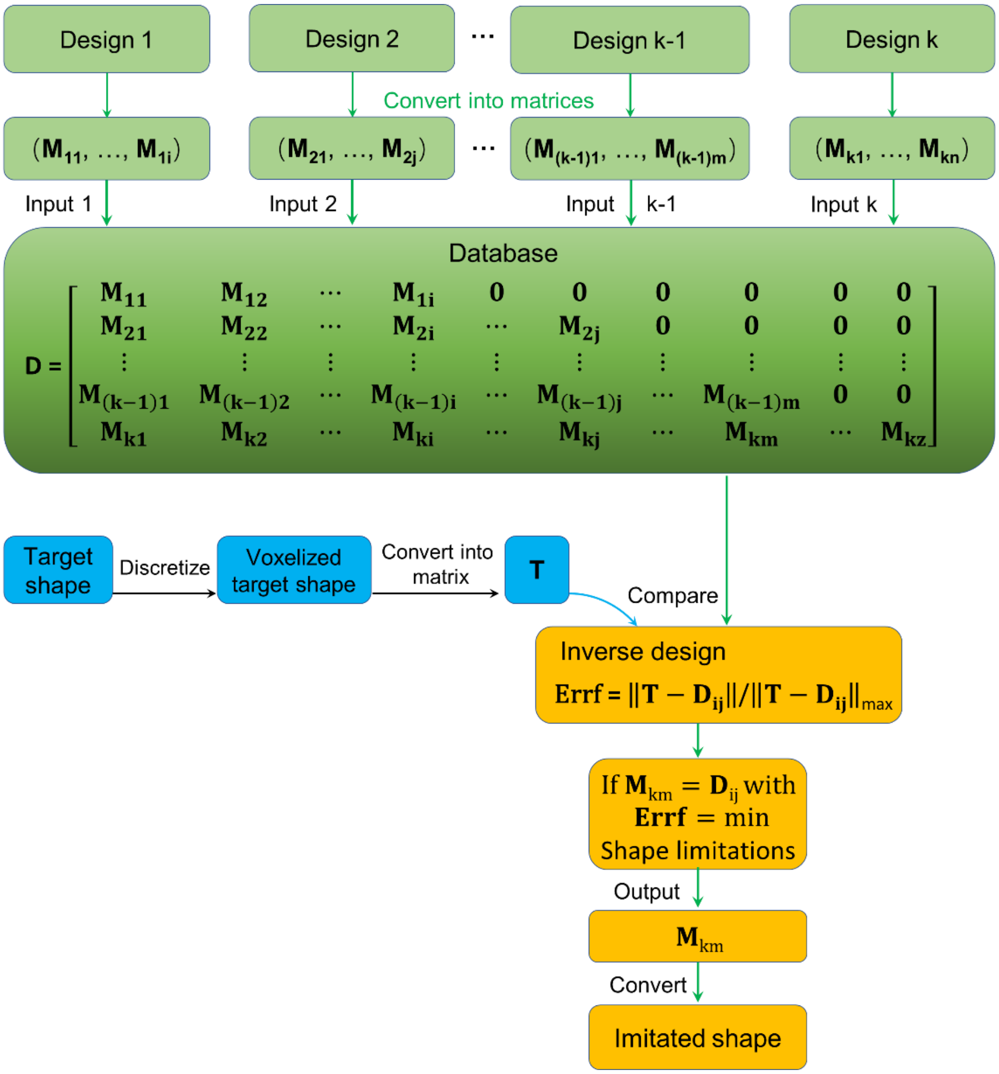

Supplementary Fig. 25. The flow chart for inverse design logic to imitate target shapes.

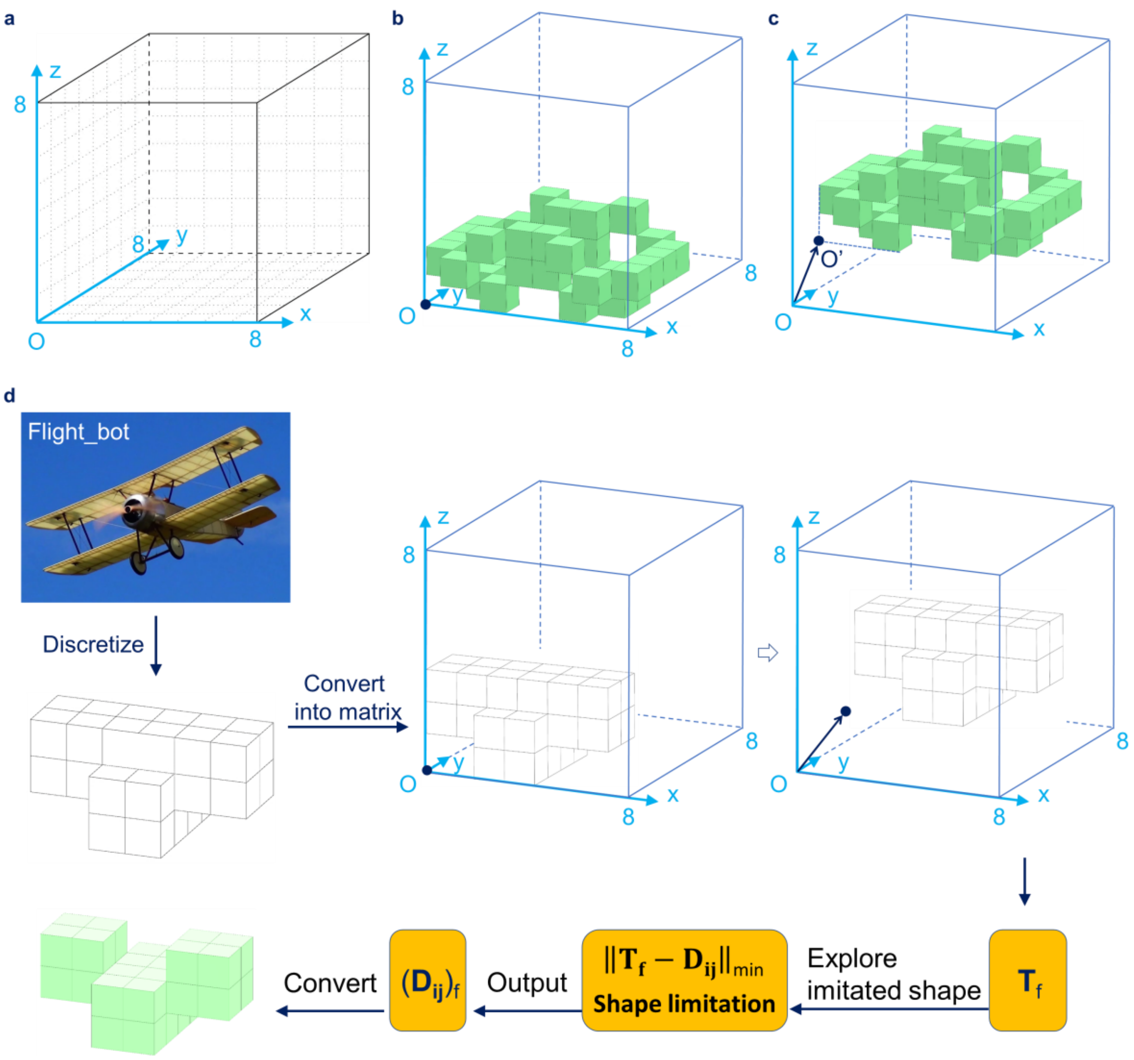

Supplementary Fig. 26. Demonstration of the inverse design to imitate a flight robot shape. **a** to **c**, Schematic illustration of the translation (**b** and **c**) of center of reconfigured shapes to the center of the selected Cartesian coordinate systems (**a**) with $0 \le x, y, z \le 8$. **d**, Inverse design process of imitating the flight shape robot: from discretization to imitation.

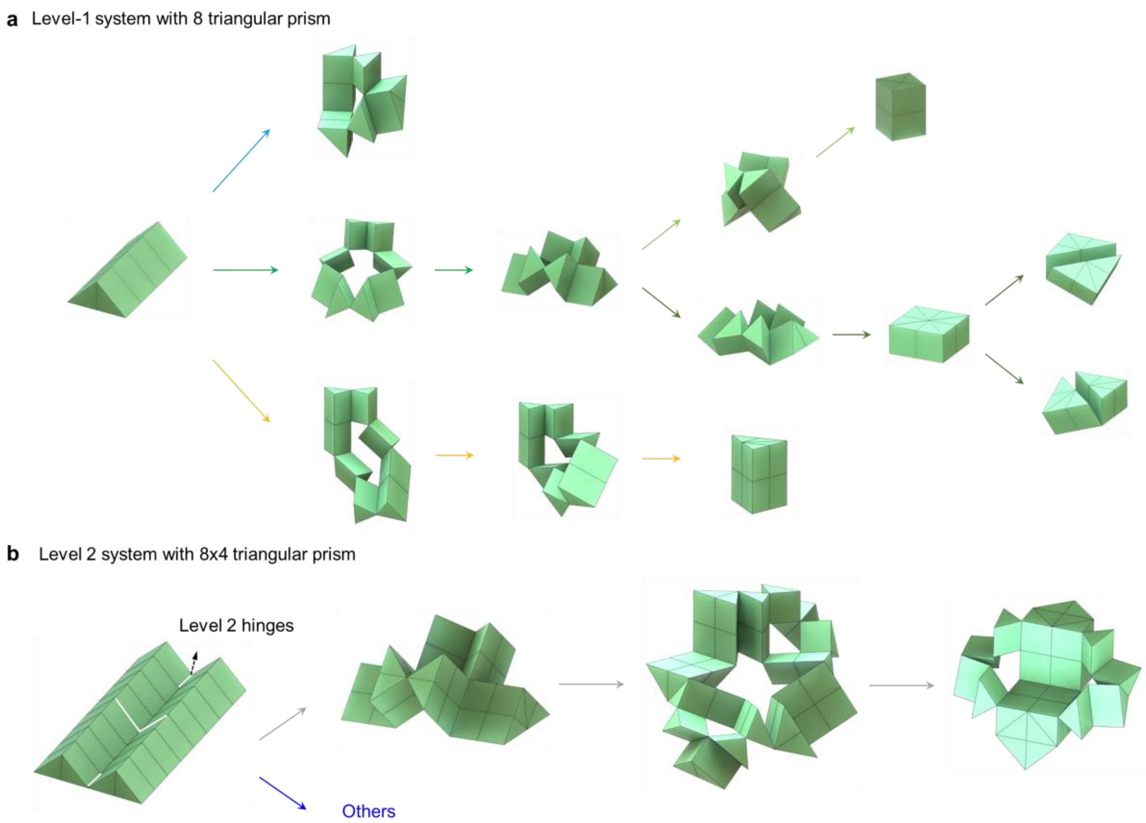

Supplementary Fig. 27. Reconfiguration details of the combinatorically designed level-1 and level-2 structures by changing the shape of structural component from cube to prism with triangular shaped cross section. a, Level-1 system and its reconfigured configurations. **b**, Level-2 structure and its representative reconfigured shapes.

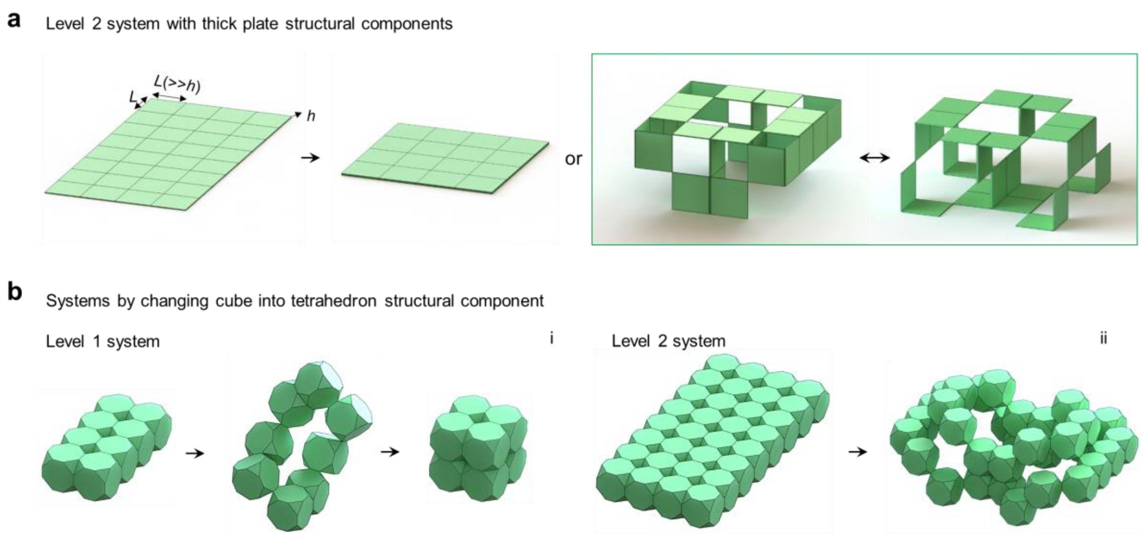

Supplementary Fig. 28. Combinatorial design of <8R, 4R> type of level-2 systems by changing the shapes of structural components: a, thin plate; **b**, tetradecahedron shape.

Captions for Supplementary Movies

Supplementary Movie 1: Shape reconfiguration of level-2 metastructure in Figure 2b

Supplementary Movie 2: Fabrication of cubes and level-2 metastructure in Figure 3a by assembly of 3D multimaterial printed thin plates into cubes and further into building blocks

Supplementary Movie 3: Selected reconfiguration processes of level-2 metastructure in Figure 3a

Supplementary Movie 4: Autonomous reconfigurable robots based on level 1 system actuated by untethered electrical servo-motor

Supplementary Movie 5: Rolling locomotion of level 2 system actuated by untethered electrical servo-motor

Supplementary Movie 6: Autonomous reconfigurable robots based on level 2 system actuated by untethered electrical servo-motor

Supplementary Movie 7: Fabrication and reconfigurations of meter-scale samples

Supplementary Movie 8: Verification of the reconfigurations of the level-2 structures by self-developed control system
