# Supplementary material for: Adaptive hierarchical origami-based metastructures
Source: Nat Commun. 2024 Jul 26;15:6247. doi: 10.1038/s41467-024-50497-5 (PMC11282231; doi:10.1038/s41467-024-50497-5)
Supplement: Supplementary file 3 — Description of Additional Supplementary Files [file 41467_2024_50497_MOESM3_ESM.pdf]

Description of Additional Supplementary Files

File Name: Supplementary Movie 1
Description: Shape reconfiguration of level-2 metastructure in Figure 2b

File Name: Supplementary Movie 2
Description: Fabrication of cubes and level-2 metastructure in Figure 3a by assembly of 3D multimaterial printed thin plates into cubes and further into building blocks

File Name: Supplementary Movie 3
Description: Selected reconfiguration processes of level-2 metastructure in Figure 3a

File Name: Supplementary Movie 4
Description: Autonomous reconfigurable robots based on level 1 system actuated by untethered electrical servo-motor

File Name: Supplementary Movie 5
Description: Rolling locomotion of level 2 system actuated by untethered electrical servo-motor

File Name: Supplementary Movie 6
Description: Autonomous reconfigurable robots based on level 2 system actuated by untethered electrical servo-motor

File Name: Supplementary Movie 7
Description: Fabrication and reconfigurations of meter-scale samples

File Name: Supplementary Movie 8
Description: Verification of the reconfigurations of the level-2 structures by self-developed control system
